# Comparative analysis of the effects of different purification methods on the yield and purity of cow milk extracellular vesicles

**DOI:** 10.1002/jex2.149

**Published:** 2024-04-22

**Authors:** Santeri Kankaanpää, Markus Nurmi, Markus Lampimäki, Heidi Leskinen, Anni Nieminen, Anatoliy Samoylenko, Seppo J. Vainio, Sari Mäkinen, Lauri Ahonen, Juha Kangasluoma, Tuukka Petäjä, Sirja Viitala

**Affiliations:** ^1^ Natural Resources Institute Finland Jokioinen Finland; ^2^ Institute for Atmospheric and Earth System Research (INAR) / Physics University of Helsinki Helsinki Finland; ^3^ Metabolomics Unit, Institute for Molecular Medicine Finland University of Helsinki Helsinki Finland; ^4^ University of Oulu, Kvantum Institute, Infotech Oulu, Faculty of Biochemistry and Molecular Medicine, Disease Networks Research Unit Oulu University Oulu Finland

**Keywords:** bioaerosol, extracellular vesicles, isolation protocol, milk, validation

## Abstract

Isolation of extracellular vesicles (EV) has been developing rapidly in parallel with the interest in EVs. However, commonly utilized protocols may not suit more challenging sample matrixes and could potentially yield suboptimal results. Knowing and assessing the pitfalls of isolation procedure to be used, should be involved to some extent for EV analytics. EVs in cow milk are of great interest due to their abundancy and large‐scale availability as well as their cross‐species bioavailability and possible use as drug carriers. However, the characteristics of milk EVs overlap with those of other milk components. This makes it difficult to isolate and study EVs individually. There exists also a lack of consensus for isolation methods. In this study, we demonstrated the differences between various differential centrifugation‐based approaches for isolation of large quantities of EVs from cow milk. Samples were further purified with gradient centrifugation and size exclusion chromatography (SEC) and differences were analyzed. Quality measurements were conducted on multiple independent platforms. Particle analysis, electron microscopy and RNA analysis were used, to comprehensively characterize the isolated samples and to identify the limitations and possible sources of contamination in the EV isolation protocols. Vesicle concentration to protein ratio and RNA to protein ratios were observed to increase as samples were purified, suggesting co‐isolation with major milk proteins in direct differential centrifugation protocols. We demonstrated a novel size assessment of vesicles using a particle mobility analyzer that matched the sizing using electron microscopy in contrast to commonly utilized nanoparticle tracking analysis. Based on the standards of the International Society for Extracellular Vesicles and the quick checklist of EV‐Track.org for EV isolation, we emphasize the need for complete characterization and validation of the isolation protocol with all EV‐related work to ensure the accuracy of results and allow further analytics and experiments.

## 1 INTRODUCTION

Milk is a complex biofluid, which covers all the energy and nutritional needs of mammalian infants (Andreas et al., 2015; Pereira, 2014). Milk contains nutrients such as carbohydrates, proteins, lipids, minerals and vitamins as well as various biologically active components and cells, that ensure species‐typical post‐natal development (Andreas et al., 2015; Pereira, 2014). Milk is also abundant in extracellular vesicles (EVs), that may originate from multiple cellular sources such as milk‐secreting epithelial cells and immune cells, and even bacteria present in milk (Sanwlani et al., 2020). EVs can be roughly classified into three main subtypes, small EVs including exosomes (sEV; 30–150 nm), microvesicles (100‐1000 nm) and apoptotic bodies (50‐5000 nm), based on their size, biogenesis and origin (Kalra et al., 2016). This classification is however incomplete as these EV subtypes are likely to have many distinct but unknown subpopulations (Willms et al., 2016, 2018). Until we can characterize and phenotype individual vesicles, we study EVs as heterogenic vesicle populations.

Cow milk sEVs have received a lot of attention because dairy products play an important role in the Western diet from childhood to adulthood. Some studies have suggested that milk‐derived sEV can mediate sophisticated signals not only between mother and infant, but also across species (Benmoussa & Provost, 2019; Melnik et al., 2013). Whether these effects are of any importance for human health and welfare is not yet known. However, there is evidence that milk sEVs tolerate dairy processing (Benmoussa et al., 2016, 2017; Pieters et al., 2015), survive mammalian digestive conditions without degradation (Benmoussa et al., 2016; Izumi et al., 2012; Wolf et al., 2015) and can enter the bloodstream from the gut and accumulate in peripheral tissues (Kusuma et al., 2016; Manca et al., 2018; Wolf et al., 2015), and. The durability, potential tissue specificity and low level of immune responses (Admyre et al., 2007; Manca et al., 2018; Samuel et al., 2017) have made milk sEVs also interesting research targets for the development of orally administered and tissue‐specific carriers of biomolecules and drugs (Zhong et al., 2021). In addition, milk sEVs could be suitable as biomarkers for monitoring the health and welfare of cows in modern dairy farms applying automated milking robots and monitoring systems. Understanding the role of milk sEVs and their bioactive components as inter‐organismal mediators of developmental signals from mother to its newborn is an interesting matter itself and may help to improve the composition of milk formulas (van Herwijnen et al., 2016).

In recent years, several methods have been suggested for milk sEV isolation and purification, but they all have different advantages and disadvantages (reviewed by Li et al., 2022). Milk is a complex body fluid containing a mixture of macro‐ and supramolecular components such as milk fat globules (MFGs), casein micelles and whey proteins, as well as immune and epithelial cells and bacteria. MFGs are considered the main contaminant of milk sEV isolation, because their size range in whole milk is partly overlapping with the EVs (0.2 µm–15 µm; Martini et al., 2016). In addition, different processing steps of dairy products, such as separation, pasteurization and homogenization affect the size of MFGs and potentially EVs (Lopez, 2005). MFGs can be distinguished from EVs by their high triacylglycerol (TAG) content (Mather & Keenan, 1998). Another major source of contamination is the milk proteins, from which the caseins are self‐assembled into nanosized colloidal suspension structures in milk called casein micelles (154–230 nm; de Kruif et al., 2012). Caseins represent about 80% of total proteins of cow milk (Uniacke‐Lowe & Fox, 2011). Also, whey proteins are abundant in milk (18% of the total proteins) and although they do not form a micelle structure, they tend to co‐isolate with other milk components and contaminate sEV isolate (Rahman et al., 2019). Milk proteins are well characterized and easily identifiable from mass spectrometric (MS) data. Their properties in milk can be influenced by adjusting pH and ionic properties of the milk sample (Josephson, 1972; Lucey et al., 1996).

Differential ultracentrifugation applied in this study, is the most frequently used method for isolating sEVs from various sources (Li et al., 2022). It is inexpensive and relatively easy to implement if factors affecting separation efficiency such as centrifugal force, rotor parameters and solution viscosity are carefully determined (Cvjetkovic et al., 2014; Izumi et al., 2015). Although the method is considered as the “golden standard” of sEV isolation, it is not effective as such in removing many co‐isolated components of milk (Jeppesen et al., 2019; van Niel et al., 2018). For this reason, the method is often complemented with other methods, such as density gradient centrifugation (DGC) and size exclusion chromatography (SEC) to remove the unwanted impurities (Vaswani et al., 2017). DGC enhances separation as the particles move and settle according to the densities within inert density gradient matrix. Reinhardt et al. (2012) and van Herwijnen et al. (2016) applied successfully sucrose density gradient method to study the proteome of the milk EV fraction. SEC can also be applied to isolate milk EVs without ultracentrifugation or to remove protein contaminants from the sEV isolate (Blans et al., 2017).

The choice of EV isolation method is influenced by the intended use and application of the EV isolates. We are interested in molecular composition of milk sEVs. For this, we aim for EVs of a certain size class with minimal contaminants from the milk matrix. To achieve that, we compared multiple differential centrifugation‐based isolation processes in terms of yield and purity of sEVs and analyzed co‐isolating and contaminating components in different methodological combinations. For that, we used nanoparticle tracking analysis (NTA), transmission electron microscopy (TEM), proteomic and lipidomic analysis, and RNA quantity analysis. We also applied and tested a differential particle mobility sizer (DMPS) as an alternative method for determining particle size distribution of EV isolates, to improve the accuracy of size estimation compared to the commonly used NTA.

## 2 MATERIALS AND METHODS

### 2.1 Isolation of EV fractions

In this study, we used raw dairy milk as a starting material to isolate EVs by differential ultracentrifugation. Milk was obtained from 80 to 100 Nordic Red cows housed in the Jokioinen research barn of the Natural Resources Institute Finland (Luke). Cold tank milk (4°C), pooled together from all cows, was collected, and kept cold prior to vesicle isolation. The milk was processed on the same morning, approximately 1 h after collection from the barn. Excess volume of milk (1‐2 L) was processed in the initial centrifugation steps and 18.5 mL of whey was used in the final centrifugation step to pellet the vesicles. The isolation was done from three biological replicates, that is, milk from three different dates.

**FIGURE 1:**
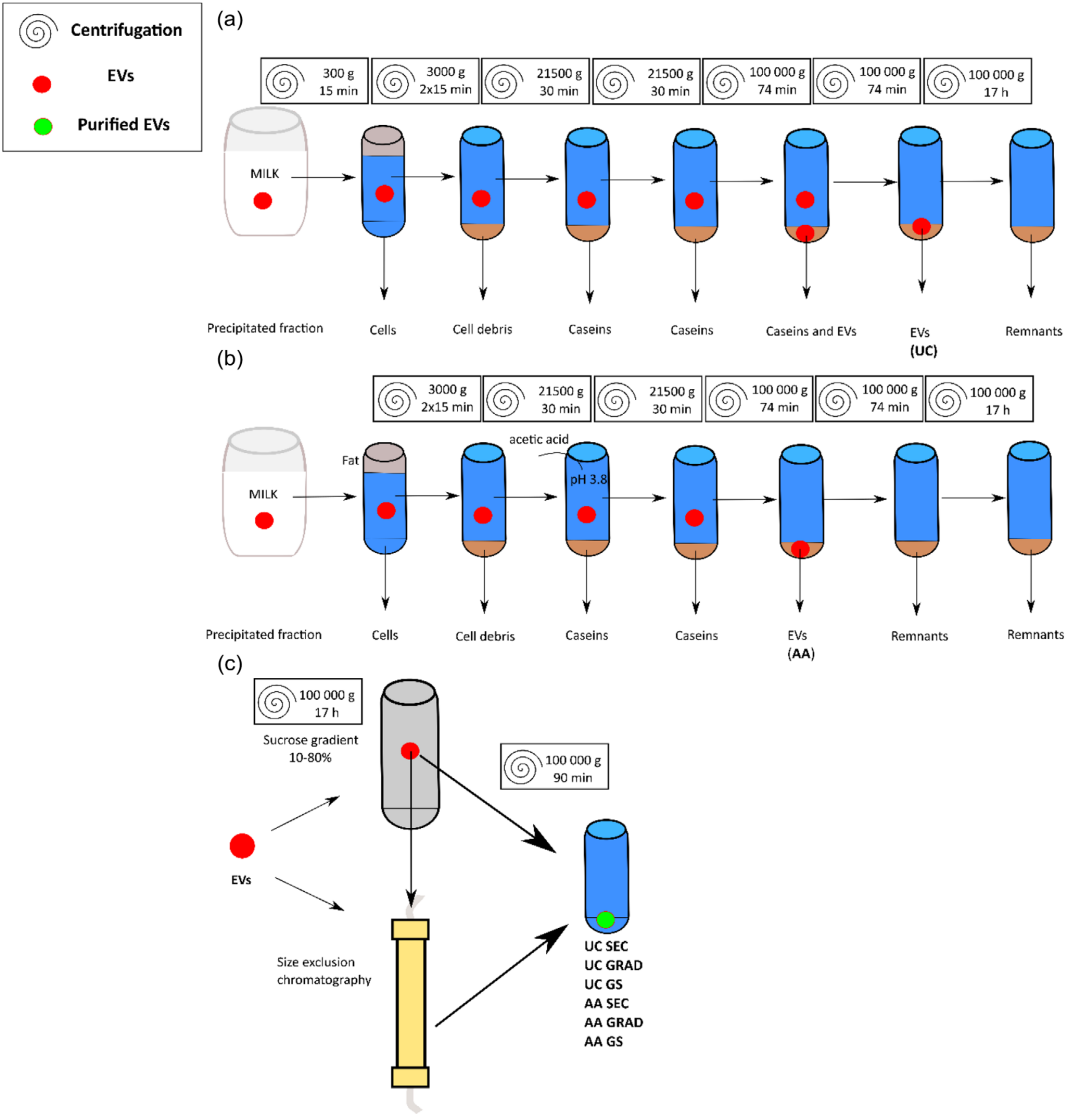
Milk small extracellular vesicles (sEV) isolation by differential centrifugation‐based protocol (sample UC) (a) combined with acetic acid (sample AA) (b) and additional purifications with sucrose density gradient (samples UC GRAD and AA GRAD), size exclusion chromatography (samples UC SEC and AA SEC) or combination of them (UC GS and AA GS) (c).

First, the milk was centrifuged to remove fat and intact cellular content. The defatted milk was either directly used for differential centrifugation followed with ultracentrifugation (resulting in sample UC, Figure 1a) or treated with acetic acid to precipitate the majority of the free proteins, caseins and peptone protein fraction, followed by ultracentrifugation (resulting to sample AA, Figure 1b). Ultracentrifugation‐enriched EVs were used for DGC with sucrose density gradient (resulting to samples UC GRAD and AA GRAD, Figure 1c) to separate EVs from the cell organelles and milk components of different density. For further separation of EVs from the co‐isolating milk proteins and to evaluate the level of free proteins in the samples, SEC was used on the samples after ultracentrifugation, or ultracentrifugation combined with DGC (resulting to samples UC SEC and AA SEC and UC GS and AA GS respectfully, Figure 1c). Samples were collected from each step of the isolation processes (Figure 1) for further analyses. Supplementary Table T1 lists the EV samples, and major control samples and the corresponding analysis performed on each sample.

#### 2.1.1 Protocol for EV isolation with differential centrifugation

Isolation was conducted as follows (Figure 1). Raw whole milk was centrifuged for 15 min at 300 × *g* and at 4°C. Cell pellet and separated, approximately 0.5 cm thick disk of fat on top were discarded and the middle layer was used in all the subsequent steps. The middle layer was further centrifuged for 2 × 15 min, 3000 × *g*, 4°C, to separate remaining fat and cellular debris. After this step, pH of the defatted milk was adjusted to 3.8 with 17.4 M glacial acetic acid (Sigma‐Aldrich) in order to precipitate free proteins and peptone fraction of proteins (AA sample isolation process; Figure 1b). Samples were then centrifuged for 30 min, 21,500 × *g*, 4°C to pellet caseins. Centrifugation was then repeated (30 min, 21,500 × *g*, 4°C) to pellet remaining caseins and cellular debris. Low‐speed centrifugations were conducted with Beckman Coulter rotors JA‐14 (bottles 250 mL, polypropylene bottle with screwcap, Beckman Coulter, 356011) or JA‐17 (bottles 50 mL, polycarbonate bottle, screwcap, 357002 Beckman Coulter) in the 21,500 × *g* steps depending on the sample volume. In the low‐speed centrifugations, excess volume was used to prepare adequate volume for the final ultracentrifugation steps.

The resulting solution (18.5 mL/tube) was ultracentrifuged for 74 min, 100,000 × *g*, 10°C, (Type 50.2 Ti, 26.3 mL polycarbonate bottle with cap assembly, Beckman Coulter 355618) to pellet EVs using an acetic acid precipitated method (AA) method. The resulting supernatant was ultracentrifuged again with the same settings to pellet EV fraction using the UC method. The pellets from the previous two ultracentrifugation steps containing EVs were resuspended to 250 µL of phosphate‐buffered saline (137 mM NaCl, 2.7 mM KCl, 10 mM Na_2_HPO_4_, 1.8 mM KH_2_PO_4_, pH 7.4; (‘Phosphate‐buffered saline (PBS)’, 2006) Cold Spring Harbour Protocols). The supernatant from the second ultracentrifugation was further centrifuged for 17 h, 100,000 × *g*, 10°C (SW 41 Ti, in Ultraclear™ tubes, Beckman Coulter 344085) to pellet the remaining particles from the solution. Centrifugation times and speeds were calculated using an online calculator (http://vesicles.niifhm.ru/index.php?do=1). EV fractions from the centrifugation are indicated as UC (without acetic acid treatment, Figure 1a) and AA (with acetic acid treatment, Figure 1b) with additional purifications described as UC SEC and AA SEC, UC GRAD and AA GRAD (sucrose DGC) or both UC GS and AA GS; (Figure 1c) in the following analytics. All samples were stored at −80°C for further purification and analysis.

#### 2.1.2 Gradient centrifugation

Layered gradient of sucrose was pipetted in 2 mL steps, as described by Luthe (1983), with gradient steps of 20%, 30%, 40%, 60%, and 80% (w/v) of sucrose in PBS in decreasing order in Ultraclear™ tubes (Beckman Coulter) in a SW 41 rotor. Gradient fractions were frozen at −80°C between each step to prevent mixing in gradient preparation. UC and AA samples (PBS suspended vesicle pellet) were pipetted onto the top fraction of the gradient tube and centrifuged for 17 h, 100000 × g, 22°C with SW‐41 rotor. Approximately 1 mL fractions were collected from the tube and refraction of the fractions was measured with a Pocket PAL‐1 refractometer (Atago Co. LTD, Japan). Fractions with a calculated density of 1.05−1.2 g/mL were collected and pooled as the EV fraction and diluted to 20 mL with PBS and pelleted with ultracentrifugation for 90 min, 100000 × *g*, 10°C with Type 50.2 Ti rotor, resulting in UC GRAD and AA GRAD samples. The UC GRAD and AA GRAD were further purified by SEC as described in 2.1.3. The resulting void peak fractions were pelleted with ultracentrifugation resulting in UC GS and AA GS samples. Samples were stored at −80°C for further analysis.

#### 2.1.3 Size exclusion chromatography

Suspended vesicle pellet (UC; 3.68e11 vesicles based on calculations from NTA and AA; 3.13e11 vesicles, 1.08e11 and 1.83e11 for UC GRAD and AA GRAD) was diluted to 600 µL with PBS with to final concentration of 20 mM EDTA and filtered through 0.13 cm 0.45 µm hydrophilic‐PP membrane acrodisc filters (Pall Co.) to remove larger than 0.45 µm protein aggregates and possible precipitates to prevent system clogging. Filtered sample (500 µL) was injected to Äkta Basic chromatography system (GE Healthcare). Sample was purified with two successively connected Superose™ 6 columns (300 × 10 mm i.d.; GE Healthcare) or with one Superdex™ 200 column (300 × 10 mm i.d.; Pharmacia Biotech) with 20 mM EDTA in PBS (Blans et al., 2017). as running buffer with 0.7 mL/min flow rate in room temperature. EVs were recovered from the void volume of the column; peak from 7.5 to 8.5 mL with Superdex™ 200 column and 13–15 mL with two successive Superose™ 6. Resulting fractions were diluted to 20 mL with PBS and pelleted with ultracentrifugation as with GRAD samples, resulting in UC SEC, and AA SEC samples. Samples were stored at −80°C for further purity analysis.

### 2.2 Analysis of small EV isolates

#### 2.2.1 Protein and peptide concentration

Protein concentration was measured in three replicates using a BioRad™ DC™ protein assay kit (BioRad), following a microplate procedure with reagent S in the mixture. Bovine serum albumin (Thermo Fisher Scientific) in PBS was used as a standard. Results are provided in Supplementary and compiled in Table 1. Peptide concentration was measured with Nanodrop One (Thermo Fisher Scientific) prior to MS analysis.

**TABLE 1:** Comparison of protein concentration, particle concentration (NTA), vesicle/protein and RNA/protein ratios for UC, UC SEC, UC GRAD and their AA counterparts.

	UC	AA UC	UC SEC	AA SEC	UC GRAD	AA GRAD
EV sample protein (µg/µL) (per mL of milk)	9.3 (125)	5.9 (79)	0.78 (10.6)	0.63 (8.5)	0.87 (11.7)	1.25 (16.9)
Vesicle concentration (particles/mL of milk, NTA)	1.47E+12	1.25E+12	2.19E+11	3.01E+11	4.08E+11	5.14E+11
Vesicle/µg protein	2.92E+09	4.47E+09	5.21E+09	8.83E+09	8.68E+09	7.61E+09
RNA (pg)/protein (µg)	1.81	4.19	3.29	7.52	7.25	7.76
RNA (ng/µL)	4.22	6.18	0.64	1.18	1.57	2.42

*Note*: RNA concentration based on Qubit four fluorometer microRNA assay.

#### 2.2.2 Electrophoresis and western blot

The proteins of sEV isolates were separated and analyzed using SDS‐PAGE and western blotting (WB). A total of 10 µg of protein per sample were solubilized in Laemmli sample buffer [138 mM Tris‐HCl, pH 6.8 (Sigma‐Aldrich), 6 M urea (Sigma‐Aldrich), 4.3% w/v SDS (Merck), 22% w/v glycerol (Merck), 5% v/v 𝛽‐mercaptoethanol (Sigma‐Aldrich) and bromophenol blue (Merck)], incubated for 30 min at 37°C and then centrifuged for 5 min at 14000 × *g*. Protein separation was performed using 15% polyacrylamide gel [0.192 M Tris‐HCl, pH 8.0 for SDS‐PAGE or pH 8.8 for WB (Sigma‐Aldrich), 3.35 M urea (Sigma‐Aldrich), 0.22% SDS w/v (Merck), 12% w/v acrylamide (19:1 acrylamide/bis‐acrylamide 30% solution, BioRad), 0.04% v/v tetraethyl methyl diamine (TEMED, GE healthcare) and 0.025% w/v ammonium persulfate (Amersham Biosciences)] with running buffer (192 mM glycine, 25 mM Tris, 3.5 mM SDS). Electrophoresis was run with a BioRad Tetra‐cell electrophoresis dock with a current of 15 mA/gel. Separated proteins were transferred to PVDF membrane (BioRad) or stained with Sypro Ruby (BioRad) according to manufacturer's protocols. Proteins were transferred using SemiDry transfer unit (BioRad) with 25 V and up to 1 mA/cm^3^ as transfer conditions. Tris‐Glycine buffer (2.5 mM Tris, 19.2 mM glycine, BioRad) with 20% methanol was used as transfer buffer.

For detection of target proteins, membranes were first blocked using 5 % w/v fish gelatin (Thermo Fisher) in Trizma buffered saline (TBS; 17.5 mM TRIS‐HCl, pH 7.5, 0.5 M NaCl) for 1 h. Then the membranes were incubated with primary antibodies [rabbit anti‐ALIX 1:2500 (cell signalling, (E6P9B) Rabbit mAb #92880), mouse anti‐CD81 1:1000 (Santa Cruz biotechnologies, (B‐11): sc‐166029), rabbit anti‐TSG101 1:2500 (Nordic BioSite, ABB‐709, ABB‐OTVG5L‐100) and mouse anti‐bovine CD63 1:500 (BioRad, MCA2042GA)] overnight on mild rocking at 4°C in 1% Tween‐20 ‐TBS (TTBS) with 1% fish gelatin. Secondary antibodies (Anti‐rabbit, AS 09 602, (Agrisera Sweden) and anti‐mouse Sc‐516102, (Santa Cruz Biotechnology)) were incubated in 1:2500 for CD63, 1:5000 for TSG101 and CD81 and 1:10000 for ALIX dilutions at room temperature on a rocking platform in TBS + 0.1% TWEEN‐20 (TTBS, Sigma‐Aldrich) for 2 h. Membranes were then washed twice with TTBS and once with TBS for 5 min prior to enhanced chemiluminescence reaction (0.1 M Tris‐HCl, pH 8.5, 0.198 mM p‐coumaric acid (Sigma‐Aldrich), 1.25 mM Luminol (Sigma‐Aldrich) and 0.9% hydrogen peroxide (Merck); Membranes and gels were imaged using Chemidoc MP (BioRad).

#### 2.2.3 Particle size measurements

Particle size was measured using NTA with a Nanosight LM14C nanoparticle tracking machine (Malvern Panalytical) for five replicates at the EV‐CORE facility in the University of Helsinki. Results from five 30 s measurements were averaged and mean particle size was calculated. Camera level was set at 14 with detect threshold at 4. TEM was carried out by the EM Unit at the University of Helsinki with JEOL JEM‐1400 according to Puhka et al (Puhka et al., 2017). To determine the physical characteristics of EVs, we performed size classification of aerosolized EVs with differential mobility particle sizer (DMPS; (Aalto et al., 2001) in the size range from 10 to 300 nm in electrical mobility equivalent diameter. EV samples from acetic acid‐treated milk (250 µL) were thawed in 17 mL of Milli‐Q water and further diluted by 1/3 for analysis. The setup for the aerosol generation and size distribution measurement is shown in supplementary Figure S1. In the first stage, an atomizer aerosol generator (TOPAS) was employed to produce EV particles in the aerosol phase. Flow through the atomizer (∼7 L/min) was measured by variable area flow metre (Kytola A‐3BR) and particles were then charged via an ^241^Am bipolar diffusion charger. No additional diffusion or dryer etc. was used. An exhaust for overflow with HEPA filter (TSI, USA) was used before the flow metre to avoid any overpressure building up. Particle number size distribution was classified by electrical mobility with the Vienna‐type Mobility particle size spectrometer (DMA), with a classification length of 30 cm. Linear ramp of the DMA voltage corresponding to particle diameter range from 10 to 300 nm was produced with the LabView controlled 12.5 kV HV source (Fug HCN 7E‐12500). With the current settings, measurement time per scan was 10 min. Sheath flows in and out were monitored by mass flow metres (TSI 4000). Sample flow rate to sheath flow rate of 1:20 was used with ∼20 L/min for the sheath and 1 L/min for the sample. The total concentration of sample particles was measured by a Condensation Particle Counter (CPC; TSI 3010, USA) connected to the laboratory vacuum line and sampling 1 L/min via a critical orifice. The cut‐off size for the CPC was around 10 nm with the current settings (TSI 3010 Specifications, 2019; (Mordas et al., 2008). Particle number size distribution for the size range from 20 to 200 nm determined by DMPS setups is typically within the uncertainty range of ±10% (Wiedensohler et al., 2012).

In order to monitor the instrument performance, reference polystyrene latex spheres (PSL Nanosphere, 100 nm ± 3 nm mean diameter, Thermo Scientific) were measured prior to the experiments. An average size distribution obtained from separate scans over the size distribution was used. AA, AA GS and AA GRAD distributions were averaged from three scans, AA SEC sample from two scans. The flow scheme of the entire setup was isolated from the compressed air and vacuum lines by HEPA filters. AA samples were chosen for analysis based on their improved purity compared to UC counterparts.

#### 2.2.4 RNA isolation and analysis

Total RNA was isolated from final PBS‐dissolved EV pellets (UC, AA, UC SEC and AA SEC and UC GRAD and AA GRAD) with total exosome RNA isolation kit (TERI, Invitrogen) using a slightly modified kit protocol. All reagents were prepared according to manufacturer's guidelines. Sample was mixed with 1:1 denaturing solution and incubated on ice for 5 min. Equal volume of acid‐phenol‐chloroform reagent was added, and the mixture was mixed by inverting the tube for 3 to 5 min. Samples were then vortexed for 1 min and inverted again 5–10 times. Samples were then centrifuged with a tabletop centrifuge (Eppendorf 5425) for 5 min at 13,000 × *g*. Upper aqueous phase was carefully extracted to a new tube and 1.25 × of extracted volume pure ethanol was added and mixed thoroughly. Resulting mixture was added on the filter provided in the kit and centrifuged for 10,000 × *g* for 15 s. This was repeated until the whole sample was passed through the filter. Samples were then washed once with 700 µL of wash buffer one and twice with 500 µL of wash buffer ⅔ with centrifugation in between washes as above. Filters were then spun dry for 1 min at 10,000 × *g* and transferred to clean collection tubes. Purified RNA was eluted from the filters twice with 50 µL of RNAse‐free water. All centrifugations were carried out at room temperature. RNA extraction was not done for GS samples due to limited sample quantity. Resulting RNA was aliquoted and frozen at −80°C until analysis. RNA concentration was measured with Qubit 4 (Thermo Fisher Scientific) with microRNA assay kit (Thermo Fisher Scientific), Nanodrop One (Thermo Fisher Scientific), and purity and peak profile with Bioanalyzer 2100 with RNA 6000 Pico kit according to manufacturer's instructions (Agilent).

#### 2.2.5 Proteome analysis

Samples (*n* = 3) without acetic acid treatment were chosen for proteome analysis, to assess the effects of each of the additional purification protocols.100 µg of protein was mixed with 8 M urea in 10 mM Tris‐HCl (pH 8) buffer, and sample was loaded to the 3 kDa cut‐off centrifugal filter tube (Pall Co.). Sample was centrifuged 10,000 × *g* for 5 min and 100 µL of urea buffer was added to the tube followed by centrifugation at 14,000 × *g* for 30 min (Eppendorf Minispin plus). Proteins were reduced with 10 mM dithiothreitol (DTT) in the urea buffer, followed by centrifugation at 14,000 × *g* for 40 min. 30 mM iodoacetamide (IAA) in the urea buffer was added to the sample and after 15 min incubation in the dark, samples were centrifuged at 14,000 × *g* for 40 min. After IAA, the sample was washed twice with urea buffer followed by centrifugation (14,000 × *g*, 40 min). The centrifugal device was transferred to a clean collection Eppendorf tube and 4 µg of trypsin/lysC protease (Promega) was added to top of the sample. Digestion was done at 37°C for 14 h. Peptides were centrifuged (14,000 × *g*, 30 min.) through the filter and 0.5 M NaCl was added to the filter followed by a second centrifugation (14,000 × *g*, 30 min).

Peptides were purified with Sep‐Pak C18 50 mg vacuum cartridges (WAT054955, Waters Corp.) according to the manufacturer's protocol on the 20‐position extraction manifold (Waters Corp.). In short, peptides were loaded into pre‐activated C18 columns and washed with 0.1% formic acid. Peptides were then eluted with 80% acetonitrile, 0.1% formic acid, dried in vacuum centrifuge (SpeedVac, Thermo Scientific) and stored at −80°C. Before injection to the LC‐MS, dried peptides were suspended to 1% formic acid in milli‐Q water and diluted to achieve suitable peptide concentration for the MS analysis.

The Sep‐Pak treated peptide samples were separated with nano‐UPLC (Acquity UPLC M‐Class, Waters Corp.) equipped with nanoEase M/Z HSS C18 T3 100Å column (Waters Corp.) and nanoE MZ Sym C18 Trap column (20 mm × 180 µm i.d., 5 µm particle size; Waters Corp.) with 0.3 µL/min flow of water/acetonitrile and linear gradient of 99:1 (vol/vol) to 40:60 (vol/vol) for 90 min with 5 min trapping. Column temperature was 35°C. UPLC was coupled to Xevo G2 MS with Zspray nano lockspray (Waters Corp.). The mass spectrometer was operated with MSe acquisition (data independent acquisition with alternating low‐energy CID and high‐energy CID) ramping between 15 and 35 V in positive ion mode. Leucine‐enkephalin (556.2771 *m/z*) was used as a lock mass calibration compound. Three biological replicates and two separate runs were conducted for one sample.

Data was analyzed with Progenesis QI for proteomics software (Waters Corp.). Data search was done by using Swiss‐Prot *Bos taurus* library with search parameters set to two missed cleavages, precursor mass tolerance 10 ppm, fragment mass tolerance 0.02 Da. Fixed modification was set for carbamidomethylation of cysteine and variable modifications were oxidation of methionine and acetylation of protein N‐terminus. False discovery rate was set to 1 %. Three peptides per protein (where one unique) were required for the identification. Label‐free quantification was done by using a between‐subject design (ANOVA) and normalized to all proteins. Relative quantitation using non‐conflicting peptides was utilized. Minimum of three peptide per protein were used and peptides were manually reviewed. Hits were filtered to proteins showing a significant 2‐fold or greater change. Comparison between treatments was done by following changes to UC‐treated samples. Quantification was done according to “Progenesis QI for proteomics user guide version 4,2”—Waters manual.

#### 2.2.6 Lipidome analysis

Acetic acid‐treated EV samples AA, AA GRAD, AA SEC, and AA GS were aliquoted and stored in MQ‐water and analyzed for lipidome as three biological replicates. Acetic acid‐treated samples were chosen for lipidomic analysis based on their outperformance of UC samples in every other analysis. Lipids were extracted with a liquid‐liquid extraction method using methyl tert‐butyl ether (MTBE) and methanol by first adding 75 µL LC‐MS grade water to a 25 µL thawed concentrated nanovesicle sample. Next, 25 µL of labelled internal standard mixture (prepared as per SCIEX LIPIDYZER manual's instructions) was added and allowed to equilibrate with the samples. To each tube, 575 µL MTBE and 160 µL of methanol were added followed by vortexing and shaking in the monophase at room temperature for 30 min. Subsequently, 200 µL water was added and samples were centrifuged 3 min at 14,000 × *g* at room temperature and the upper layer of MTBE was collected in borosilicate glass tubes. Extraction was repeated by adding 300 µL MTBE, 100 µL methanol, and 100 µL water to PCR tubes, and the upper layer was combined with previous MTBE extract and dried under N_2_. Finally, the dried samples were reconstituted with 250 µL of mobile phase [dichloromethane:methanol (50:50, vol/vol) containing 10 mM ammonium acetate] for injection. Lipid separation and quantitation were performed on the SCIEX Lipidyzer™ platform using a SCIEX 5500 QTRAP® MS (SCIEX, Washington, D.C.) with SelexION® Differential ion mobility (DMS) technology, by directly infusing 50 µL of extracted samples with a mobile phase at a flow rate of 70 µL/min. Two acquisition methods, with and without SelexION® technology, were used to cover 13 lipid classes using flow injection analysis. The lipid molecular species were measured using MRM strategy in both positive and negative polarities. Positive ion mode was used for the detection of the following lipid classes: sphingomyelins (SM), diacylglycerols (DAG), cholesteryl esters (CE), ceramides (CER), dihydroceramides (DCER), hexosylceramides (HexCER), lactosylceramides (LacCER), and TAG. Negative ion mode was used for the detection of lysophosphatidylethanolamines (LPE), lysophosphatidylcholines (LPC), phosphatidylcholines (PC), phosphatidylethanolamines (PE), ether‐linked PE (PE‐P, PE‐O), and free fatty acids (FFA). Lipidomics Workfow Manager software was used for acquisition of samples, automated data processing, signal detection and lipid species concentration calculations (Ghorasaini et al., 2021).

## 3 RESULTS

### 3.1 Acetic acid treatment improved yield and purity of EVs

**FIGURE 2:**
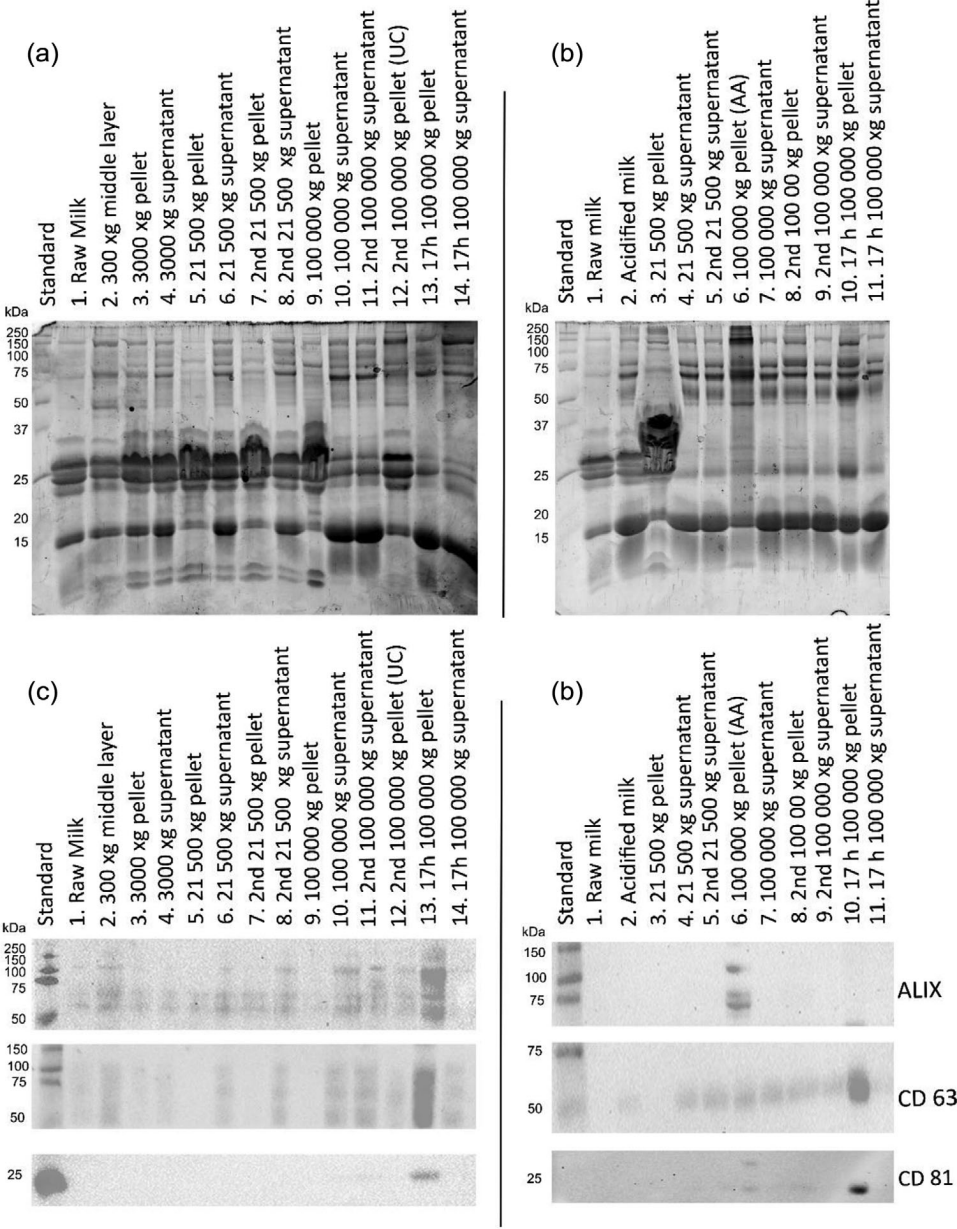
SDS‐PAGE (panel a and b) and western blot (panel c and d) of fractions from EV‐enrichment process. (a) and (c) sEV protein composition throughout centrifugation process, the lane numbers indicated the same samples. (b and d) EV composition throughout ultracentrifugation process with additional acetic acid treatment, the lane numbers indicate the same samples between western blot and SDS‐PAGE.

**FIGURE 3:**
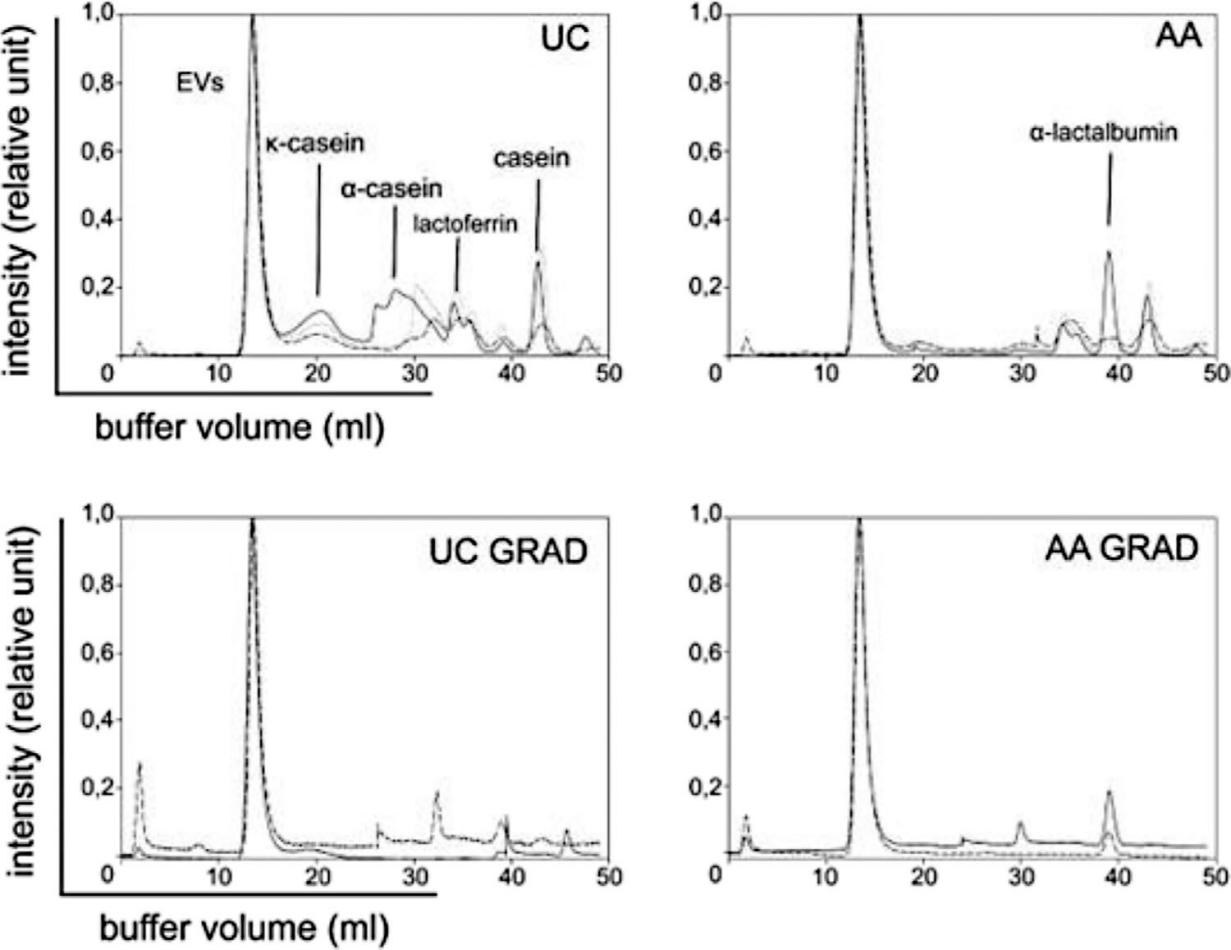
Size exclusion chromatograms of UC, AA, UC GRAD, and AA GRAD separated on two successive Superose™ six columns.

**FIGURE 4:**
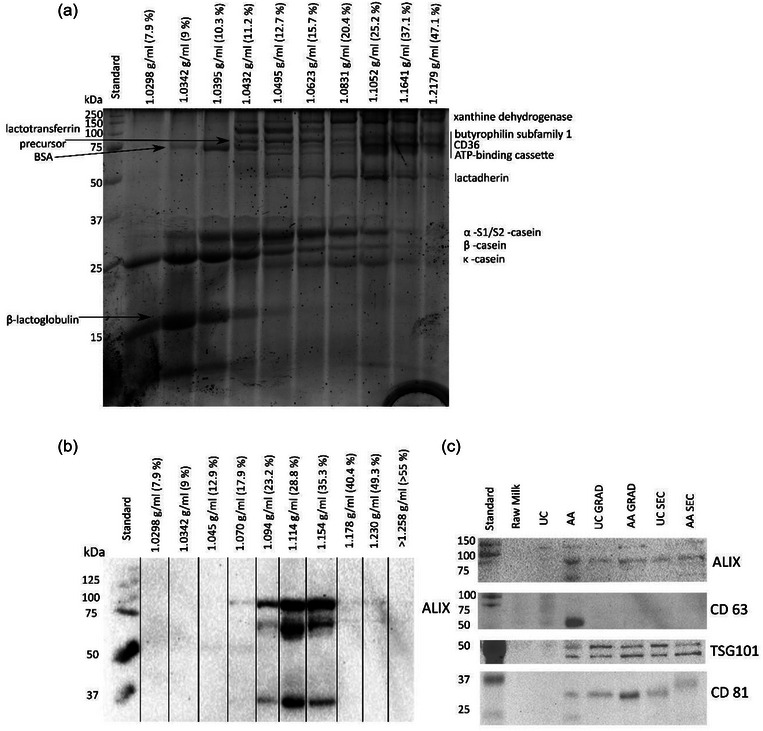
Sucrose density gradient centrifugation impact on the sEV protein composition. (a) SDS‐PAGE of fractions from UC purification, (b) Western blot of fractions from AA purification by anti‐ALIX. (c) Western blot of EV‐associated sEV‐fractions (UC, AA) and purified EV fractions (UC GRAD and AA GRAD and UC SEC and AA SEC).

**FIGURE 5:**
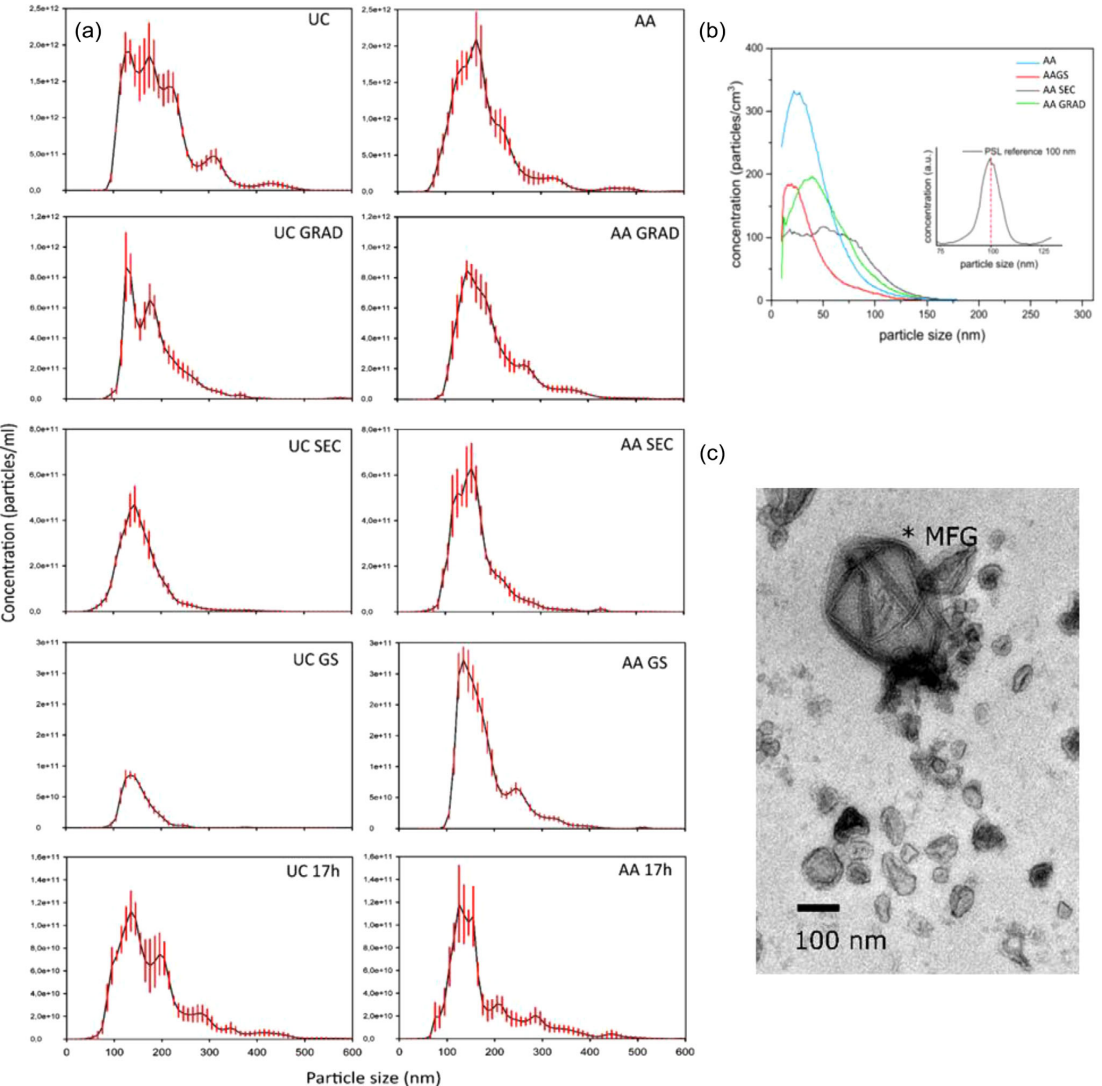
(a) NTA results from EV samples and controls. (b) DMPS results and size distribution of AA‐process samples; 100 nm bead control provided in figure PSL reference). (c) TEM micrograph close‐up of EVs and MFG obtained from UC GRAD sample. Wide angle TEMs are provided in supplemental file S1 Figure S16‐S24 from all EV samples.

According to SDS‐PAGE results, the pH adjustment to 3.8 using acetic acid after removal of cell debris (Figure 2a,b) precipitated large fraction of milk proteins in differential centrifugation protocol (Figure 2b lane 3). Without acetic acid precipitation, caseins with size range of 20−30 kDa, remain as a significant contaminant throughout the isolation (Figures 2a and 3; SEC UC). Acetic acid treatment also showed improved vesicle/protein and RNA/protein ratios, indicating better EV yield and purity (Table 1 and Figures 4c and 5a, supplementary Table T2 and supplementary FiguresS2 and S3). Acetic acid treatment affected antibody affinity (CD63) where sharper bands were observed in WB profiles (Figure 2c,d). No distinctive differences between AA and UC samples were seen in TEMs (Figure 5 and supplementary Figures S16‐S25) but small change in NTA size distribution was seen (Figure 5, UC vs. AA).

### 3.2 SEC improves separation efficiency of sEV from soluble milk proteins

**FIGURE 6:**
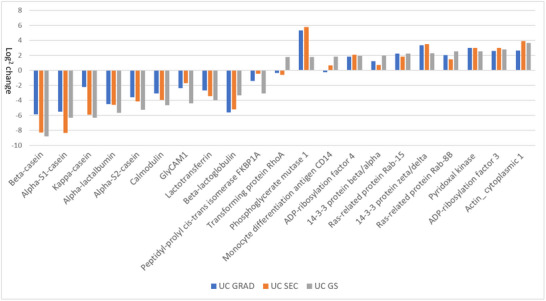
Comparison of the relative protein amounts of the 10 most decreased and the 10 most enriched proteins in LC‐MS proteomics. Comparison is presented between ultracentrifuge isolated (UC, set at 0) sEVs and sEV samples prepared by sucrose gradient centrifugation (UC GRAD), size exclusion chromatography (UC SEC) and successive use of both methods (UC GS). All statistically different proteins are seen in supplementary Figure S6.

**FIGURE 7:**
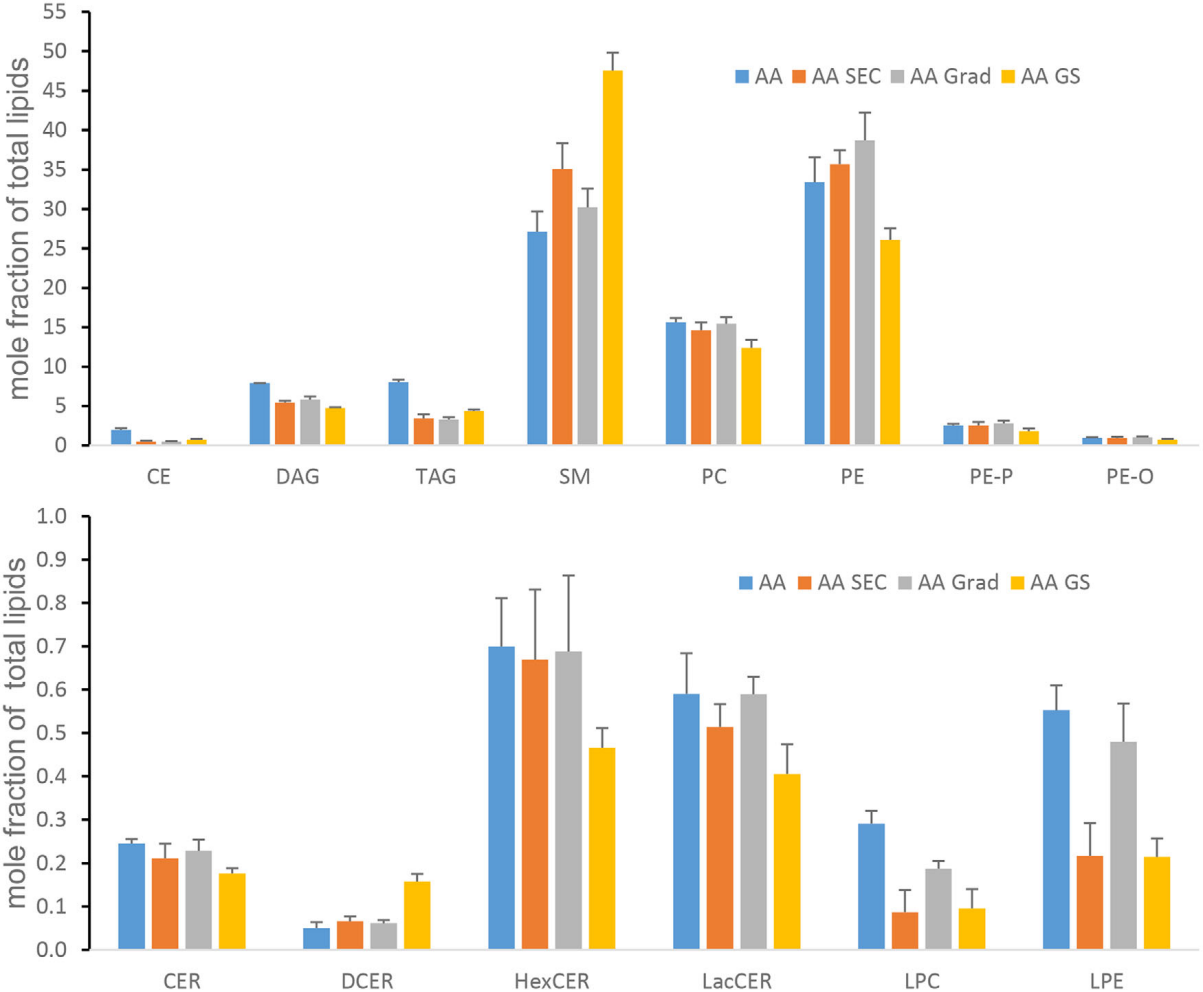
Relative mole fractions of lipid classes (*n*/*n* ± SD) in acetic acid‐treated ultracentrifuged sample (AA) and samples purified with gradient centrifugation (AA GRAD), size exclusion chromatography (AA SEC), and with both methods (AA GS) with three biological replicates. CE, cholesteryl ester; CER, ceramide; DAG, diacylglycerol; DCER, dihydroceramide; FFA, free fatty acid; HexCER, hexosylceramide; LacCER, lactosylceramide; LPC, lysophosphatidylcholine; LPE, lysophosphatidylethanolamine; PC, phosphatidylcholine; PE‐O, ether‐linked PE; and PE‐P vinyl ether‐linked PE; PE, phosphatidylethanolamine; SM, sphingomyelins; TAG, triacylglycerol.

Utilization of commercially available SEC columns provide a quick and easy method to separate particles larger than the columns exclusion limit. Suitable running buffer for SEC was required for separation of EVs from soluble proteins, but the removal of milk caseins requires chelating agent such as EDTA to dissociate the casein micelle structures which otherwise would elute in the same void volume as EVs (data not shown). SEC was effective in separating soluble proteins including dissociated milk caseins from EVs (Figure 3). The observation is supported by the detection of sEV markers (CD81, TSG101 and ALIX; Figure 4c) in WB and the improvement of the vesicle/protein and RNA/protein ‐ratios after SEC (Table 1). The enrichment of EV‐associated proteins and the reduced amount of milk proteins (caseins, whey proteins) was also observed in proteomics data of SEC‐isolated sEVs (Figures 3 and 6). Similarly, as with gradient centrifugation, decrease of TAG was also observed after SEC purification indicating that the SEC or additional ultracentrifugation step separates some MFGs from EVs as well (Figure 7). In this study, SEC was also used to detect the purity of EV isolates after each isolation process. As expected, UC‐isolated EV sample contained a considerable amount of milk proteins. Although the other methods (AA, UC GRAD, AA GRAD) improved the purity of EVs in some measure, still soluble milk proteins were detectable in all isolates (Figure 3, supplementary Figure S5).

### 3.3 Sucrose density gradient alone is not a sufficient method to separate EVs from non‐vesicular particles of milk

DGC improves separation of vesicles from some of the milk proteins, such as β‐lactoglobulin (Figure 3, GRAD samples, Figure 4a). Lactadherin, a membrane‐bound protein also shown to be present in milk sEV proteome as well as in MFG, localized mostly in the density fractions corresponding to the EV density confirming the expected placement of EVs in the density fractions determined by refractometer (1.05‐1.15 g/mL; Figure 4a). WB with anti‐ALIX revealed expected signal in fractions of the same density (Figure 4b). From other investigated EV marker proteins TSG101 and CD81, but not CD63, were identified on UC GRAD and AA GRAD samples (Figure 4c). Increased vesicle/protein ratio, as well as RNA/protein ratio of GRAD samples, indicated improved sample purity compared to UC samples (Table 1). Reduction of milk proteins was also observed in SEC chromatograms (Figure 3, UC GRAD and AA GRAD samples) as well as in proteomics data, where sEV‐associated proteins enriched, and milk proteins decreased compared to UC samples (Figure 6). Decrease of TAGs characteristics for MFGs indicated that DGC reduces also contaminating MFGs in the sample (Figure 7). Localization of EVs in density gradient was visually distinguishable after DGC (Supplementary Figure S4).

### 3.4 Sizing and characteristics of EV samples: DMPS, TEM, and NTA

Size and characteristics of particles/vesicles of final isolates were analyzed for each isolation process and controls with NTA and TEM. In NTA, the majority of the particles were in the size range of 100–200 nm (Figure 5a,c). Particle concentration ranged from 1.47e12 (UC) to 2.19e11 (UC SEC) in 1 mL of raw milk (Table 1). Acetic acid treatment narrowed the particle size distribution in UC samples (UC vs. AA UC, Figure 5a) by decreasing the amount of >200 nm particles (Figure 5a).

TEM reveals vesicles, that correspond to EVs, based on their appearance and size. Most vesicles were less than 100 nm in size, but also smaller ≤50 nm aggregating particles were observed (UC GS; Supplementary Figure S19). In addition, MFGs were identified by their characteristic crystallization of fat in TEM (Figure 5, *MFG). No distinctive visual differences were observed between different isolation processes. Size assessment with DMPS narrows the particle size distribution (<150 nm) compared to NTA, better corresponding observation in TEM.

### 3.5 Biomolecules reveal differences in sEV isolation methods

To study the influence of DGC and SEC techniques separately and combined on the enrichment of EV‐associated proteins, the relative abundance of LS‐MS‐detected proteins from UC GRAD, UC SEC, and UC GS were compared to UC (Figure 6). Using GRAD and SEC or their combination, the proportion of sEV‐associated proteins (e.g., CD14 and Rav‐8B) increased, while the proportion of milk proteins (e.g., β‐lactoglobulin and caseins) decreased. Increasing trend was also observed in several other EV markers and associated proteins such as CD9 and annexin 5, and RAB‐8B, RAB‐15 and CD14 respectively (Supplementary Figure 6). Based on our data, SEC and DGC were efficient in removing milk proteins from the isolated samples. SEC favoured the removal of soluble proteins, such as caseins, and DGC was more efficient in the removal of MFGs based on GlyCAM1 abundance (Figure 6), which is heavily enriched in MFGs, but also secreted in soluble form.

Caseins (β‐casein, α‐caseins, κ‐casein) and whey proteins (lactotransferrin, calmodulin, β‐lactoglobulin, α‐lactalbumin) were successfully removed especially with SEC. Complete list of all proteins identified is found in supplemental file S2.

Lipidomics was used in verifying the purity of the AA GRAD, AA SEC and AA GS isolates compared with AA samples (i.e., only UC‐purified AA‐treated samples). Lipid class analysis showed isolation protocol‐specific changes for the majority of classes. Based on the lipid class analysis, the most purified sample, AA GS, contained the highest proportion (mole fraction) of FFA (50.9 ± 1.90%; data not shown) compared with AA, AA GRAD, and AA SEC (18.2 ± 2.01, 18.8 ± 2.64, and 23.3 ± 5.45%, respectively) when FFA were considered as part of the total lipids studied. However, further examination of the results was conducted without FFA (Figure 7 and supplementary Figure S7 and S8).

Lipidomic results supported data from proteomics and SDS‐PAGE/WB. The lower proportion of CE and TAG as well as DAG in EV isolates AA GRAD, AA SEC, and AA GS indicates lower quantity of MFGs compared with the AA isolate, which was only purified by UC (Figure 7). On the contrary, the mole fractions of SM were higher in the AA GRAD, AA SEC, and AA GS isolates than in AA. The mole fractions of PE, PC, LacCER, and HexCER seemed to be the lowest in AA GS isolates, while the differences in PE‐P and PE‐O were minor between isolates. The structural compositions of some of the lipid classes also changed during the purification process as presented in the Supplementary Figure S7–S15.

**FIGURE 8:**
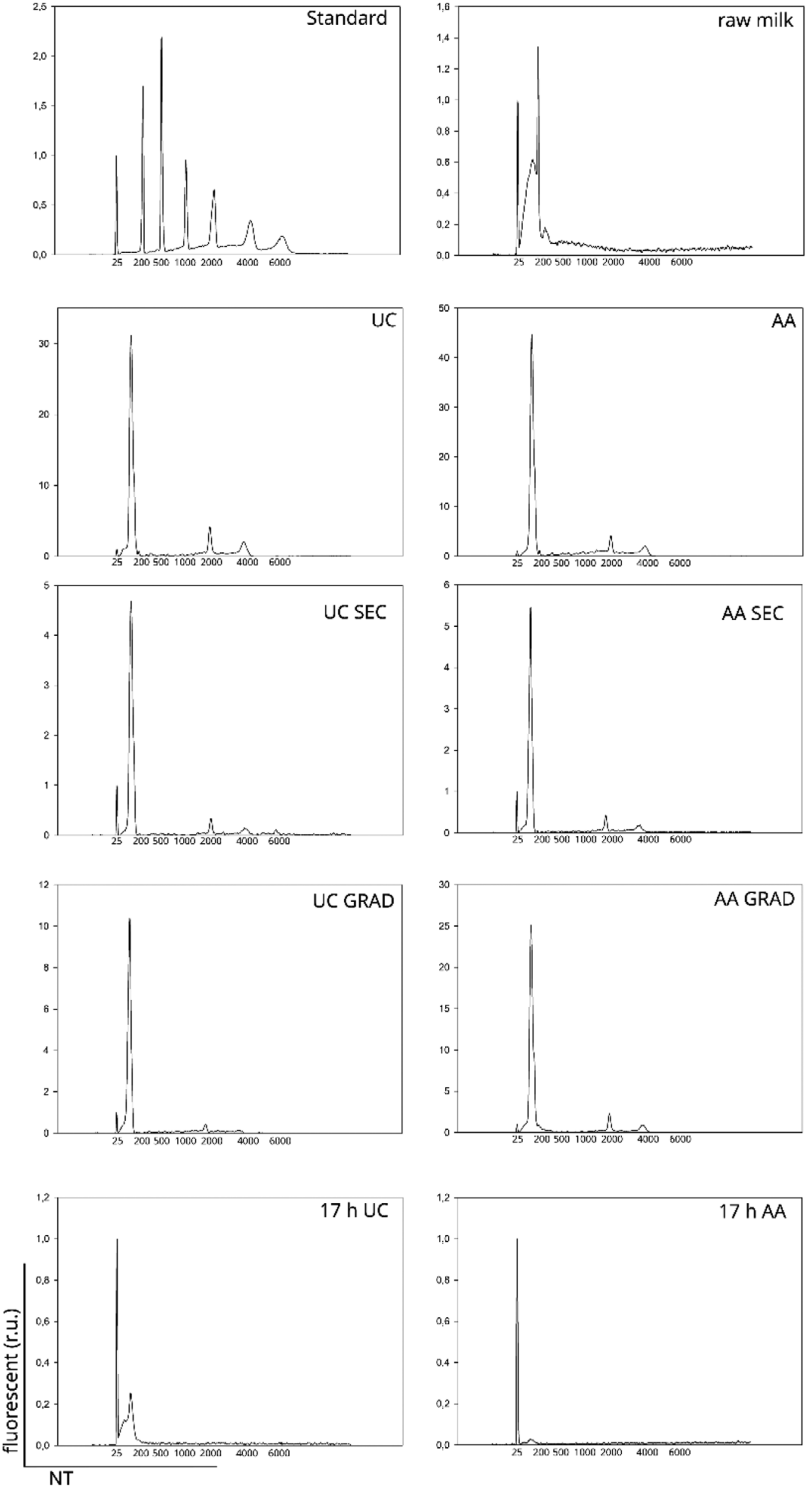
RNA profiles of different EV isolates. EV‐enrichment was conducted with ultracentrifugation without (UC) or with acetic acid (AA) continued either by size exclusion chromatography (UC SEC and AA SEC) clean‐up or sucrose gradient centrifugation (UC GRAD and AA GRAD). 17‐h UC samples were carried out to analyze “left‐over” fraction after the vesicle pellet was removed from ultracentrifugation steps. The size of the RNA fragments calculated by nucleotides (nt) are estimated using size standard. Each sample contains 25 nt internal standards. The internal standard was normalized to fluorescent value 1 in all samples.

RNA profile was utilized as an indicator of EVs in the sample assuming that most of milk RNA was encased in EVs. Bioanalyzer data showed that the majority of the isolated RNA was small RNA (Figure 8) and that the isolation methods had no impact on RNA profile. However, the RNA yield decreased with further isolations with GRAD and SEC (Figure 8, Table 1). Analysis of isolated pellets from 17 h ultracentrifugation revealed little to no RNA, suggesting that the supernatant after sEV isolation (UC and AA samples) did not contain intact EVs.

## 4 DISCUSSION

Extraction of EVs from complex matrices such as milk (D'Alessandro et al., 2011; Singh & Gallier, 2017) requires understanding of the pitfalls of the isolation method to be used. In our experiment, we focused on differential centrifugation‐based protocols to isolate sEVs from milk, and identified and characterized the possible contaminants and assessed the suitable method for the follow‐up analyses. We also implemented novel particle size analysis to better measure the size distribution of isolated EVs. Our data reveals the isolation protocol‐specific changes in proteome, lipidome and RNA yield, which were used to evaluate the purity of the isolates.

### 4.1 Assessment of EV isolation methods

In this study, we showed the importance of extensive validation of the isolation procedures for EVs. The guidelines presented by ISEV (Lötvall et al., 2014; Théry et al., 2018) and easy‐to‐follow 9‐point validation of EV‐TRACK (EV‐TRACK; Consortium et al., 2017) provide a good framework to work with. However, to further improve the isolation procedures, extensive validation with more than one method should generally be incorporated in the studies to characterize the fraction nominated as sEVs. Our results showed that, based on WB analysis only, the most valid milk sEV or exosome‐enriched fraction would be the pellet after 17 h ultracentrifugation. However, RNA yield, electron microscopy images and the calculations of the pelleting efficiency of centrifugations suggest otherwise. The results also indicated that antibody testing alone is not sufficient for identification of the fractions of interest. Based on a single validation method and without the characterization of all the fractions, it could be possible to end up discarding the target of analysis as an unwanted fraction or either use the unintended fraction or overlook the possible contaminants. In the present study, the contaminants are quite easily identified as general milk proteins which are in abundance in milk.

Isolation method based on differential centrifugation is effective to enrich large quantities of EVs from bovine milk. Removal of milk fat and casein micelles is, however, required for improved yield and purity and this can be achieved by acidification (Rahman et al., 2019; Tan et al., 2021; Yamauchi et al., 2019) or micelle disruption by EDTA or sodium citrate (Benmoussa et al., 2016; Vaswani et al., 2017). Our data supports that by adjusting pH of defatted milk to 3.8, the yield and the purity of sEV isolation improves as also previously reported (Li et al., 2022; Morozumi et al., 2021; Tong et al., 2020; Yamauchi et al., 2019). The effect of acidification is based on the isoelectric precipitation of casein micelles (Dalgleish & Corredig, 2012; Yamauchi et al., 2019).

The separation of sEVs from milk proteins can be further improved with SEC, which is effective in separating soluble proteins from the EV fractions. In our experiment, caseins were co‐isolating with EVs when a chelating agent was not present in the running buffer to disrupt the micelle formation similarly as in Blans et al. (2017). However, results with effective removal of caseins with SEC without casein micelle disruption have also been published recently (del Pozo‐Acebo et al., 2021).

Based on our data it is possible to select isolation processes to work with specific fractions of the cow milk sEVs. Extensive purification of milk EVs is not necessarily required for small RNA‐related workflows, since more extensive purifications reduce the yield. If the origin of the small RNA pool is of consideration, additional purification would be required to exclude possible non‐EV sources of RNA, such as of ribosomal origin (Hansen et al., 2022). Sequencing of the different sEV pools from different isolation processes would reveal if these populations differ from one another. However, if the follow‐up analyses are sensitive to protein contamination, additional purifications could be beneficial to remove co‐isolating proteins. Casein removal by adjusting pH or using enzymes could also be suitable provided that the morphology and surface structures of native milk EVs is not required for follow‐up analyses (Rahman et al., 2019; Tong et al., 2020).The effects of acidification remain under discussion, with some contradictory interpretation of data presented whether the acidification results in changes in membrane integrity and morphology (Rahman et al., 2019; Yamauchi et al., 2019).

### 4.2 EV size

The size range of EV isolates in the present study was similar to previously reported data (Benmoussa et al., 2017). When comparing the TEM images to the NTA results, a roughly 30−80 nm shift in the size of the vesicles can be observed. This could originate from the conditions, the settings used in the NTA measurement or from a hydrodynamic size effect. In addition, in the DMPS measurements, the maximum EV concentration was obtained in the size range from ∼20 to 50 nm (samples AA SEC and AA GRAD). This was in agreement with the previous studies reporting sa imilar diameter range for exosome‐like vesicles that were collected from human breast milk by differential centrifugation and analyzed by electron microscopy as well as EVs from bovine milk (Admyre et al., 2007; Samuel et al., 2021). The accurate resolution of the NTA system is limited to 30 nm in protein analytics and 60–70 nm for particles. It is also heavily dependent on the concentration of particles (Bachurski et al., 2019; Filipe et al., 2010; Maas et al., 2015). Vesicles below the analysis threshold will be ignored. NTA is also shown to overestimate particle sizes biological materials (Bachurski et al., 2019). DMPS offers more accurate measurement of sEV size which agrees with size estimation from TEM.

It should also be noted that, in DMPS, each sample in aerosol flow with the given electrical mobility always contains some particles with different sizes (if the sample is not completely monodispersed) and different charges due to multiple charging phenomena (Wiedensohler, 1988). In the differential mobility analyzer (DMA), the particles are classified by their electrical mobility and particles in the lower size range remain mainly singly charged, thus producing correct size values in the measurement. Double charging becomes more significant in particles with a size range ≥200 nm (> 10% charging probability). Even then the doubly charged particles would not be in the maximum concentration size range for EVs (∼20‐50 nm). For the triply charged particles, the charging probability is already less than 2% at <150 nm (Wiedensohler, 1988). We, therefore, conclude that the multiple charging phenomena did not considerably interfere with DMPS measurements in this study.

Hydrodynamic size of EVs obtained, for instance in the NTA analysis, is larger than the geometric size. Previously, it has been observed that geometric size of hydrated and desiccated EVs of ∼40‐70 nm shows corresponding hydrodynamic diameters in the range from ∼120 to 180 nm (Chernyshev et al., 2015). This was explained partly due to macromolecular surface conjugated compounds, which could include proteins, lipids and other compounds present in biological fluids. Similar results have also been obtained in the NTA measurements of current EV samples. NTA analysis for AA SEC samples shows ∼135 nm mean diameter. This also corresponds to the results obtained from DMPS measurements as the particle size distribution measured in aerosol phase closely resembles the EVs’ geometric size.

### 4.3 Proteomics

MS analysis of EV proteome was carried out to evaluate the effect of different isolation methods on the purity of the sample and to identify suitable candidates for EV marker proteins for bovine milk. Overall, the majority of the proteins were associated with EVs. As expected, a relative decrease of non‐EV proteins and an increase of EV‐associated proteins, such as CD14 (Gebraad et al., 2018), was seen when isolates were further purified. The comparison also showed contaminating co‐isolating proteins in UC samples, such as GlyCAM1, which is secreted by epithelial cells to milk (Dowbenko et al., 1993). Enriched proteins from the UCGS sample could potentially be used as EV markers, such as tetraspanin CD9 which associates with CD81 (Charrin et al., 2001) and ras‐related protein RAB‐8B (Gangoda et al., 2017). The origin of EVs analyzed in our experiment remains still a question. CD14‐positive EVs could potentially be originating from immune cells (Gebraad et al., 2018) while as CD9‐positive vesicles could be derived directly from plasma membrane (Mathieu et al., 2021).

In this study, relative amounts of FKBP1 and CD81 decreased along the purification process. This reduction could indicate that these proteins are not associated with the milk EV fraction but are part of the MFG (Hettinga et al., 2011) or belong to the vesicle subpopulation lost during purification. Some EV‐associated proteins, such as CD63, were detected with WB but not with MS. This could be explained by technical reasons: firstly, comparison was only made to proteins with ≥3 peptides identified from each sample and proteins with less than 3 identified peptides from each sample were excluded, and secondly the sensitivity of the MS instrument set limitations to the analysis.

A common problem with the purification is that EVs and MFGs are difficult to separate from each other due to the overlapping size and density (Singh & Gallier, 2017). In the present study a decrease in the relative amounts of known MFG proteins, such as xanthine dehydrogenase/oxidase (Farkye, 2002), were observed in GS‐purified samples, whereas those same proteins were higher when the sample was purified only with SEC or gradient centrifugation. The proteome analyses suggest that DGC followed by SEC results in more purified EV fraction with less MFG contamination.

In the present study, EV proteome was more limited than the first published bovine milk EV proteome of 2107 proteins (Reinhardt et al., 2012). However, more recent studies with more advanced methods and MS instruments reported a more compact EV proteome, suggesting significant technical differences in EV isolation methods and possibly a higher rate of false positives or contaminants in previous analyses (Rahman et al., 2021; Reinhardt et al., 2012; Somiya et al., 2018; Tan et al., 2021). The actual physical limitations of EV size could also limit the proteome size and randomly occurring singular proteins would drop below the limit of detection (Sverdlov, 2012).

### 4.4 Lipidome profile of milk

We used lipidome analysis as an additional method to verify the purification process steps from ultracentrifuged samples onwards and to characterize the lipidome profile of bovine milk EV isolates. Milk contains fat globules of different sizes and changes in the lipidome profile along the purification steps were hypothesized to reflect their presence. Lipidomic results of the present study support data from proteomics and SDS‐PAGE/WB analyses.

The fat in the lipid droplets contains high levels of TAG and CE (Skotland et al., 2019). The lower proportion of CE and TAG in EV isolates AA GRAD, AA SEC, and AA GS compared to AA isolate indicates higher purity of those isolates and the presence of MFGs in the AA isolate. In addition, the fatty acid profile of TAG and DAG changed during the purification process. We were not able to quantify the abundance of cholesterol or phosphatidyl serine, which has been shown to be enriched in EVs (Blans et al., 2017).

EVs are abundant in SM (Skotland et al., 2019). In addition, in a previous report (Blans et al., 2017), SM was reported to be more abundant in bovine milk EV preparations compared with the MFG membrane. The higher proportion of this lipid class in AA GRAD, AA SEC and especially AA GS isolates compared to AA indicates a higher density of EVs in those samples. In contrast, milk processing was shown to slightly decrease the SM percentage in milk EV samples in parallel with increase in TAG:chol ratio, indicating co‐isolation of other lipids due to milk processing (Hansen et al., 2022).

Bovine MFG membrane has been reported to be more enriched in PC than bovine milk EV preparations (Blans et al., 2017). In the present study, the molar proportion of PC decreased moderately in the purification process from AA to AA GS, indicating that the purity of EV isolates also increased. Blans et al. (2017) reported a slightly higher proportion of PE in milk EVs compared with MFG membrane, although the differences were minor. However, in the present study, the molar fraction of PE was the lowest in the most purified AA GS isolate. Contrary to Hansen et al. (2022), who reported similar proportions of PC and PE, and (Grossen et al., 2021), who reported higher proportions of PC than PE in EVs isolated from raw milk, our studies and the results by Blans et al. (2017) showed lower proportions of PC compared to PE.

In previous reports, ether lipids have been shown to be enriched in EVs released from different cell types and they may be important components contributing to membrane stability and fusion of EVs with other membranes (Skotland et al., 2019). However, in the present study, the differences in the molar fractions of PE‐P and PE‐O were rather minor between the isolates. Nevertheless, the differences in the composition of PE‐P species between AA GS and other isolates indicated changes in the membrane PE‐P along the purification process and thus in the EV composition. In addition, EVs are supposed to be rich in glycosphingolipids (HexCER, LacCER) compared with cells (Skotland et al., 2019) but, in the present study, the most purified isolate AA GS seemed to contain the lowest molar fraction of HexCER and LacCER.

The reason for the highest molar proportion of FFA in the most purified EV isolates AA GS could be a result of the extensive and long purification process, which might have affected the lipids in the samples. Nevertheless, this observation as well as the difference in the FFA composition between SEC purified samples (AA SEC and AA GS) compared with AA and AA GRAD remains to be studied. This may have implications for the further utilization of EV isolates.

### 4.5 Final remarks

We emphasize the importance of validating the used EV isolation protocol with more than one metric regardless of the isolation protocol. Based solely on a single analysis method, we would have discarded the majority of our sample and considered the 17‐h fractions as the most promising ones based on WB analytics.

We observed increased purity of sample with additional purification steps. This was observed across all analyses conducted. EVs showed limited protein and RNA profiles. Thus, the EV population is quite homologous in nature. Proteome was changed with additional purifications that removed co‐isolating proteins. The lipidome profiles indicated the lowest purity for the AA isolate and the highest purity for AA GS samples, although the FFA content for AA GS was the highest. Overall, there were differences between isolates within lipid classes, but AA GS was notably different from other EV isolates. This demonstrates the efficiency of our isolation protocol, producing reproducible results and high‐quality data to be used in further studies. We also described the limitations of the isolation procedure and characterized the impurities in detail, which can be difficult to differentiate from broad data.

## AUTHOR CONTRIBUTIONS

**Santeri Johannes Kankaanpää**: Conceptualization (equal); formal analysis (lead); methodology (lead); writing—original draft (lead); writing—review and editing (equal). **Markus Nurmi**: Formal analysis (equal); methodology (equal); supervision (equal); writing—original draft (equal); writing—review and editing (supporting). **Markus Lampimäki**: Data curation (supporting); formal analysis (supporting); methodology (supporting); writing—original draft (supporting). **Heidi Leskinen**: Formal analysis (equal); methodology (equal); writing—original draft (equal); writing—review and editing (equal). **Anatoliy Samoylenko**: Methodology (supporting); resources (supporting). **Sari Mäkinen**: Writing—original draft (supporting). **Juha Kangasluoma**: Methodology (supporting); resources (supporting). **Lauri Ahonen**: Methodology (supporting); resources (supporting). **Tuukka Petäjä**: Conceptualization (equal); funding acquisition (equal); methodology (supporting); resources (supporting). **Sirja Viitala**: Conceptualization (lead); funding acquisition (lead); project administration (lead); resources (equal); supervision (lead); writing—original draft (equal); writing—review and editing (equal).

## CONFLICT OF INTEREST STATEMENT

The authors declare no conflicts of interest.

## Supporting information

Supporting Information

Supporting Information

## Data Availability

The data that support the findings of this study are available from the corresponding author upon reasonable request.

## References

[jex2149-bib-0001] Aalto, P. , Hämeri, K. , Becker, E. , Weber, R. , Salm, J. , Mäkelä, J. M. , Hoell, C. , O'dowd, C. D. , Karlsson, H. , Hansson, H.‐C. , Väkevä, M. , Koponen, I. K. , Buzorius, G. , & Kulmala, M. (2001). Physical characterization of aerosol particles during nucleation events. Tellus B: Chemical and Physical Meteorology, 53(4), 344. 10.3402/tellusb.v53i4.17127

[jex2149-bib-0002] Admyre, C. , Johansson, S. M. , Qazi, K. R. , Filén, J.‐J. , Lahesmaa, R. , Norman, M. , Neve, E. P. A. , Scheynius, A. , & Gabrielsson, S. (2007). Exosomes with immune modulatory features are present in human breast milk. The Journal of Immunology, 179(3), 1969–1978. 10.4049/jimmunol.179.3.1969 17641064

[jex2149-bib-0003] Andreas, N. J. , Kampmann, B. , & Mehring Le‐Doare, K. (2015). Human breast milk: A review on its composition and bioactivity. Early Human Development, 91(11), 629–635. 10.1016/j.earlhumdev.2015.08.013 26375355

[jex2149-bib-0004] Bachurski, D. , Schuldner, M. , Nguyen, P. , Malz, A. , Reiners, K. S. , Grenzi, P. C. , Babatz, F. , Schauss, A. C. , Hansen, H. P. , Hallek, M. , & von Strandmann, E. P. (2019). Extracellular vesicle measurements with nanoparticle tracking analysis—An accuracy and repeatability comparison between NanoSight NS300 and ZetaView. Journal of Extracellular Vesicles, 8(1), 1596016. 10.1080/20013078.2019.1596016 30988894 PMC6450530

[jex2149-bib-0005] Benmoussa, A. , Lee, C. H. C. , Laffont, B. , Savard, P. , Laugier, J. , Boilard, E. , Gilbert, C. , Fliss, I. , & Provost, P. (2016). Commercial dairy cow milk microRNAs resist digestion under simulated gastrointestinal tract conditions. The Journal of Nutrition, 146(11), 2206–2215. 10.3945/jn.116.237651 27708120

[jex2149-bib-0006] Benmoussa, A. , Ly, S. , Shan, S. T. , Laugier, J. , Boilard, E. , Gilbert, C. , & Provost, P. (2017). A subset of extracellular vesicles carries the bulk of microRNAs in commercial dairy cow's milk. Journal of Extracellular Vesicles, 6(1), 1401897. 10.1080/20013078.2017.1401897 29904572 PMC5994974

[jex2149-bib-0007] Benmoussa, A. , & Provost, P. (2019). Milk MicroRNAs in health and disease. Comprehensive Reviews in Food Science and Food Safety, 18(3), 703–722. 10.1111/1541-4337.12424 33336926

[jex2149-bib-0008] Blans, K. , Hansen, M. S. , Sørensen, L. V. , Hvam, M. L. , Howard, K. A. , Möller, A. , Wiking, L. , Larsen, L. B. , & Rasmussen, J. T. (2017). Pellet‐free isolation of human and bovine milk extracellular vesicles by size‐exclusion chromatography. Journal of Extracellular Vesicles, 6(1), 1294340. 10.1080/20013078.2017.1294340 28386391 PMC5373680

[jex2149-bib-0009] Charrin, S. , Le Naour, F. , Oualid, M. , Billard, M. , Faure, G. , Hanash, S. M. , Boucheix, C. , & Rubinstein, E. (2001). The major CD9 and CD81 molecular partner. Journal of Biological Chemistry, 276(17), 14329–14337. 10.1074/jbc.m011297200 11278880

[jex2149-bib-0010] Chernyshev, V. S. , Rachamadugu, R. , Tseng, Y. H. , Belnap, D. M. , Jia, Y. , Branch, K. J. , Butterfield, A. E. , Pease, L. F. , Bernard, P. S. , & Skliar, M. (2015). Size and shape characterization of hydrated and desiccated exosomes. Analytical and Bioanalytical Chemistry, 407(12), 3285–3301. 10.1007/s00216-015-8535-3 25821114

[jex2149-bib-0011] Cvjetkovic, A. , Lötvall, J. , & Lässer, C. (2014). The influence of rotor type and centrifugation time on the yield and purity of extracellular vesicles. Journal of Extracellular Vesicles, 3(1), 23111. 10.3402/jev.v3.23111 PMC3967015 24678386

[jex2149-bib-0012] D'Alessandro, A. , Zolla, L. , & Scaloni, A. (2011). The bovine milk proteome: cherishing, nourishing and fostering molecular complexity. An interactomics and functional overview. Molecular BioSystems, 7(3), 579–597. 10.1039/c0mb00027b 20877905

[jex2149-bib-0013] Dalgleish, D. G. , & Corredig, M. (2012). The structure of the casein micelle of milk and its changes during processing. Annual Review of Food Science and Technology, 3(1), 449–467. 10.1146/annurev-food-022811-101214 22385169

[jex2149-bib-0014] de Kruif, C. G. , Huppertz, T. , Urban, V. S. , & Petukhov, A. V. (2012). Casein micelles and their internal structure. Advances in Colloid and Interface Science, 171–172, 36–52. 10.1016/j.cis.2012.01.002 22381008

[jex2149-bib-0015] del Pozo‐Acebo, L. , López de las Hazas, M.‐C. , Tomé‐Carneiro, J. , Gil‐Cabrerizo, P. , San‐Cristobal, R. , Busto, R. , García‐Ruiz, A. , & Dávalos, A. (2021). Bovine milk‐derived exosomes as a drug delivery vehicle for miRNA‐based therapy. International Journal of Molecular Sciences, 22(3), 1105. 10.3390/ijms22031105 33499350 PMC7865385

[jex2149-bib-0016] Dowbenko, D. , Kikuta, A. , Fennie, C. , Gillett, N. , & Lasky, L. A. (1993). Glycosylation‐dependent cell adhesion molecule 1 (GlyCAM 1) mucin is expressed by lactating mammary gland epithelial cells and is present in milk. Journal of Clinical Investigation, 92(2), 952–960. 10.1172/jci116671 8349827 PMC294935

[jex2149-bib-0017] EV‐TRACK Consortium . Van Deun, J. , Mestdagh, P. , Agostinis, P. , Akay, Ö. , Anand, S. , Anckaert, J. , Martinez, Z. A. , Baetens, T. , Beghein, E. , Bertier, L. , Berx, G. , Boere, J. , Boukouris, S. , Bremer, M. , Buschmann, D. , Byrd, J. B. , Casert, C. , Cheng, L. , Cmoch, A. , … Hendrix, A. . (2017). EV‐TRACK: Transparent reporting and centralizing knowledge in extracellular vesicle research. Nature Methods, 14(3), 228–232. 10.1038/nmeth.4185 28245209

[jex2149-bib-0018] Farkye, N. Y. (2002). ENZYMES INDIGENOUS TO MILK | Xanthine Oxidase.’, in Encyclopedia of dairy sciences. Elsevier,. Available at: 10.1016/B0-12-227235-8/00156-5

[jex2149-bib-0019] Filipe, V. , Hawe, A. , & Jiskoot, W. (2010). Critical evaluation of nanoparticle tracking analysis (NTA) by NanoSight for the measurement of nanoparticles and protein aggregates. Pharmaceutical Research, 27(5), 796–810. 10.1007/s11095-010-0073-2 20204471 PMC2852530

[jex2149-bib-0020] Gangoda, L. , Liem, M. , Ang, C. , Keerthikumar, S. , Adda, C. G. , Parker, B. S. , & Mathivanan, S. (2017). Proteomic profiling of exosomes secreted by breast cancer cells with varying metastatic potential. Proteomics, 17(23–24),. 10.1002/pmic.201600370 29115712

[jex2149-bib-0021] Gebraad, A. , Kornilov, R. , Kaur, S. , Miettinen, S. , Haimi, S. , Peltoniemi, H. , Mannerström, B. , & Seppänen‐Kaijansinkko, R. (2018). Monocyte‐derived extracellular vesicles stimulate cytokine secretion and gene expression of matrix metalloproteinases by mesenchymal stem/stromal cells. The FEBS Journal, 285(12), 2337–2359. 10.1111/febs.14485 29732732

[jex2149-bib-0022] Ghorasaini, M. , Mohammed, Y. , Adamski, J. , Bettcher, L. , Bowden, J. A. , Cabruja, M. , Contrepois, K. , Ellenberger, M. , Gajera, B. , Haid, M. , Hornburg, D. , Hunter, C. , Jones, C. M. , Klein, T. , Mayboroda, O. , Mirzaian, M. , Moaddel, R. , Ferrucci, L. , Lovett, J. , … Giera, M. (2021). Cross‐laboratory standardization of preclinical lipidomics using differential mobility spectrometry and multiple reaction monitoring. Analytical Chemistry, 93(49), 16369–16378. 10.1021/acs.analchem.1c02826 34859676 PMC8674878

[jex2149-bib-0023] Grossen, P. , Portmann, M. , Koller, E. , Duschmalé, M. , Minz, T. , Sewing, S. , Pandya, N. J. , van Geijtenbeek, S. K. , Ducret, A. , Kusznir, E.‐A. , Huber, S. , Berrera, M. , Lauer, M. E. , Ringler, P. , Nordbo, B. , Jensen, M. L. , Sladojevich, F. , Jagasia, R. , Alex, R. , … Keller, M. (2021). Evaluation of bovine milk extracellular vesicles for the delivery of locked nucleic acid antisense oligonucleotides. European Journal of Pharmaceutics and Biopharmaceutics, 158, 198–210. 10.1016/j.ejpb.2020.11.012 33248268

[jex2149-bib-0024] Hansen, M. S. , Gregersen, S. B. , & Rasmussen, J. T. (2022). Bovine milk processing impacts characteristics of extracellular vesicle isolates obtained by size‐exclusion chromatography. International Dairy Journal, 127, 105212. 10.1016/j.idairyj.2021.105212

[jex2149-bib-0025] Hettinga, K. , van Valenberg, H. , de Vries, S. , Boeren, S. , van Hooijdonk, T. , van Arendonk, J. , & Vervoort, J. (2011). The host defense proteome of human and bovine milk. PLoS ONE, 6(4), e19433. 10.1371/journal.pone.0019433 21556375 PMC3083434

[jex2149-bib-0026] Izumi, H. , Kosaka, N. , Shimizu, T. , Sekine, K. , Ochiya, T. , & Takase, M. (2012). Bovine milk contains microRNA and messenger RNA that are stable under degradative conditions. Journal of Dairy Science, 95(9), 4831–4841. 10.3168/jds.2012-5489 22916887

[jex2149-bib-0027] Izumi, H. , Tsuda, M. , Sato, Y. , Kosaka, N. , Ochiya, T. , Iwamoto, H. , Namba, K. , & Takeda, Y. (2015). Bovine milk exosomes contain microRNA and mRNA and are taken up by human macrophages. Journal of Dairy Science, 98(5), 2920–2933. 10.3168/jds.2014-9076 25726110

[jex2149-bib-0028] Jeppesen, D. K. , Fenix, A. M. , Franklin, J. L. , Higginbotham, J. N. , Zhang, Q. , Zimmerman, L. J. , Liebler, D. C. , Ping, J. , Liu, Q. , Evans, R. , Fissell, W. H. , Patton, J. G. , Rome, L. H. , Burnette, D. T. , & Coffey, R. J. (2019). Reassessment of exosome composition. Cell, 177(2), 428–445.e18. 10.1016/j.cell.2019.02.029 30951670 PMC6664447

[jex2149-bib-0029] Josephson, R. V. (1972). Isoelectric focusing of bovine milk caseins. Journal of Dairy Science, 55(11), 1535–1543. 10.3168/jds.s0022-0302(72)85716-3 4635507

[jex2149-bib-0030] Kalra, H. , Drummen, G. , & Mathivanan, S. (2016). Focus on extracellular vesicles: Introducing the next small big thing. International Journal of Molecular Sciences, 17(2), 170. 10.3390/ijms17020170 26861301 PMC4783904

[jex2149-bib-0031] Kusuma, R. J. , Manca, S. , Friemel, T. , Sukreet, S. , Nguyen, C. , & Zempleni, J. (2016). Human vascular endothelial cells transport foreign exosomes from cow's milk by endocytosis. American Journal of Physiology‐Cell Physiology, 310(10), C800–C807. 10.1152/ajpcell.00169.2015 26984735 PMC4895447

[jex2149-bib-0032] Li, X. , Su, L. , Zhang, X. , Chen, Q. , Wang, Y. , Shen, Z. , Zhong, T. , Wang, L. , Xiao, Y. , Feng, X. , & Yu, X. (2022). Recent advances on the function and purification of milk exosomes: A review. Frontiers in Nutrition, 9, 871346. 10.3389/fnut.2022.871346 35757254 PMC9219579

[jex2149-bib-0033] Lopez, C. (2005). Focus on the supramolecular structure of milk fat in dairy products. Reproduction Nutrition Development, 45(4), 497–511. 10.1051/rnd:2005034 16045897

[jex2149-bib-0034] Lötvall, J. , Hill, A. F. , Hochberg, F. , Buzás, E. I. , Di Vizio, D. , Gardiner, C. , Gho, Y. S. , Kurochkin, I. V. , Mathivanan, S. , Quesenberry, P. , Sahoo, S. , Tahara, H. , Wauben, M. H. , Witwer, K. W. , & Théry, C. (2014). Minimal experimental requirements for definition of extracellular vesicles and their functions: A position statement from the International Society for Extracellular Vesicles. Journal of Extracellular Vesicles, 3(1), 26913. 10.3402/jev.v3.26913 25536934 PMC4275645

[jex2149-bib-0035] Lucey, J. A. , Gorry, C. , O'Kennedy, B. , Kalab, M. , Tan‐Kinita, R. , & Fox, P. F. (1996). Effect of acidification and neutralization of milk on some physico‐chemical properties of casein micelles. International Dairy Journal, 6(3), 257–272. 10.1016/0958-6946(95)00014-3

[jex2149-bib-0036] Luthe, D. S. (1983). A simple technique for the preparation and storage of sucrose gradients. Analytical Biochemistry, 135(1), 230–232. 10.1016/0003-2697(83)90755-8 6670744

[jex2149-bib-0037] Maas, S. L. N. , de Vrij, J. , van der Vlist, E. J. , Geragousian, B. , van Bloois, L. , Mastrobattista, E. , Schiffelers, R. M. , Wauben, M. H. M. , Broekman, M. L. D. , & Nolte‐’t Hoen, E. N. M. (2015). Possibilities and limitations of current technologies for quantification of biological extracellular vesicles and synthetic mimics. Journal of Controlled Release, 200, 87–96. 10.1016/j.jconrel.2014.12.041 25555362 PMC4324667

[jex2149-bib-0038] Manca, S. , Upadhyaya, B. , Mutai, E. , Desaulniers, A. T. , Cederberg, R. A. , White, B. R. , & Zempleni, J. (2018). Milk exosomes are bioavailable and distinct microRNA cargos have unique tissue distribution patterns. Scientific Reports, 8(1), 11321. 10.1038/s41598-018-29780-1 30054561 PMC6063888

[jex2149-bib-0039] Martini, M. , Salari, F. , & Altomonte, I. (2016). The macrostructure of milk lipids: The fat globules. Critical Reviews in Food Science and Nutrition, 56(7), 1209–1221. 10.1080/10408398.2012.758626 24915408

[jex2149-bib-0040] Mather, I. H. , & Keenan, T. W. (1998). Origin and secretion of milk lipids. Journal of Mammary Gland Biology and Neoplasia, 3(3), 259–273. 10.1023/A:1018711410270 10819513

[jex2149-bib-0041] Mathieu, M. , Névo, N. , Jouve, M. , Valenzuela, J. I. , Maurin, M. , Verweij, F. J. , Palmulli, R. , Lankar, D. , Dingli, F. , Loew, D. , Rubinstein, E. , Boncompain, G. , Perez, F. , & Théry, C. (2021). Specificities of exosome versus small ectosome secretion revealed by live intracellular tracking of CD63 and CD9. Nature Communications, 12(1), 4389. 10.1038/s41467-021-24384-2 PMC8289845 34282141

[jex2149-bib-0042] Melnik, B. C. , John, S. M. , & Schmitz, G. (2013). Milk is not just food but most likely a genetic transfection system activating mTORC1 signaling for postnatal growth. Nutrition Journal, 12(1), 103. 10.1186/1475-2891-12-103 23883112 PMC3725179

[jex2149-bib-0043] Mordas, G. , Manninen, H. E. , Petäjä, T. , Aalto, P. P. , Hämeri, K. , & Kulmala, M. (2008). On operation of the ultra‐fine water‐based CPC TSI 3786 and comparison with other TSI models (TSI 3776, TSI 3772, TSI 3025, TSI 3010, TSI 3007). Aerosol Science and Technology, 42(2), 152–158. 10.1080/02786820701846252

[jex2149-bib-0044] Morozumi, M. , Izumi, H. , Shimizu, T. , & Takeda, Y. (2021). Comparison of isolation methods using commercially available kits for obtaining extracellular vesicles from cow milk. Journal of Dairy Science, 104(6), 6463–6471. 10.3168/jds.2020-19849 33714584

[jex2149-bib-0045] Pereira, P. C. (2014). Milk nutritional composition and its role in human health. Nutrition, 30(6), 619–627. 10.1016/j.nut.2013.10.011 24800664

[jex2149-bib-0046] Phosphate‐buffered saline (PBS) . (2006). Cold Spring Harbor Protocols. 10.1101/pdb.rec8247

[jex2149-bib-0047] Pieters, B. C. H. , Arntz, O. J. , Bennink, M. B. , Broeren, M. G. A. , van Caam, A. P. M. , Koenders, M. I. , van Lent, P. L. E. M. , van den Berg, W. B. , de Vries, M. , van der Kraan, P. M. , & van de Loo, F. A. J. (2015). Commercial cow milk contains physically stable extracellular vesicles expressing immunoregulatory TGF‐β. PLOS ONE, 10(3), e0121123. 10.1371/journal.pone.0121123 25822997 PMC4379073

[jex2149-bib-0048] Puhka, M. , Nordberg, M.‐E. , Valkonen, S. , Rannikko, A. , Kallioniemi, O. , Siljander, P. , & af Hällström, T. M. (2017). KeepEX, a simple dilution protocol for improving extracellular vesicle yields from urine. European Journal of Pharmaceutical Sciences, 98, 30–39. 10.1016/j.ejps.2016.10.021 27771514

[jex2149-bib-0049] Rahman, Md. M. , Shimizu, K. , Yamauchi, M. , Takase, H. , Ugawa, S. , Okada, A. , & Inoshima, Y. (2019). Acidification effects on isolation of extracellular vesicles from bovine milk. PLOS ONE, 14(9), e0222613. 10.1371/journal.pone.0222613 31525238 PMC6746375

[jex2149-bib-0050] Rahman, Md. M. , Takashima, S. , Kamatari, Y. O. , Badr, Y. , Kitamura, Y. , Shimizu, K. , Okada, A. , & Inoshima, Y. (2021). Proteomic profiling of milk small extracellular vesicles from bovine leukemia virus‐infected cattle. Scientific Reports, 11(1), 2951. 10.1038/s41598-021-82598-2 33536533 PMC7858626

[jex2149-bib-0051] Reinhardt, T. A. , Lippolis, J. D. , Nonnecke, B. J. , & Sacco, R. E. (2012). Bovine milk exosome proteome. Journal of Proteomics, 75(5), 1486–1492. 10.1016/j.jprot.2011.11.017 22129587

[jex2149-bib-0052] Samuel, M. , Chisanga, D. , Liem, M. , Keerthikumar, S. , Anand, S. , Ang, C.‐S. , Adda, C. G. , Versteegen, E. , Jois, M. , & Mathivanan, S. (2017). Bovine milk‐derived exosomes from colostrum are enriched with proteins implicated in immune response and growth. Scientific Reports, 7(1), 5933. 10.1038/s41598-017-06288-8 28725021 PMC5517456

[jex2149-bib-0053] Samuel, M. , Fonseka, P. , Sanwlani, R. , Gangoda, L. , Chee, S. H. , Keerthikumar, S. , Spurling, A. , Chitti, S. V. , Zanker, D. , Ang, C.‐S. , Atukorala, I. , Kang, T. , Shahi, S. , Marzan, A. L. , Nedeva, C. , Vennin, C. , Lucas, M. C. , Cheng, L. , Herrmann, D. , … Mathivanan, S. (2021). Oral administration of bovine milk‐derived extracellular vesicles induces senescence in the primary tumor but accelerates cancer metastasis. Nature Communications, 12(1), 3950. 10.1038/s41467-021-24273-8 PMC8225634 34168137

[jex2149-bib-0054] Sanwlani, R. , Fonseka, P. , Chitti, S. V. , & Mathivanan, S. (2020). Milk‐derived extracellular vesicles in inter‐organism, cross‐species communication and drug delivery. Proteomes, 8(2), 11. 10.3390/proteomes8020011 32414045 PMC7356197

[jex2149-bib-0055] Singh, H. , & Gallier, S. (2017). Nature's complex emulsion: The fat globules of milk. Food Hydrocolloids, 68, 81–89. 10.1016/j.foodhyd.2016.10.011

[jex2149-bib-0073] Skotland, T. , Hessvik, N. P. , Sandvig, K. , & Llorente, A. (2019). Exosomal lipid composition and the role of ether lipids and phosphoinositides in exosome biology. Journal of Lipid Research, 60(1), 9–18. 10.1194/jlr.r084343 30076207 PMC6314266

[jex2149-bib-0056] Somiya, M. , Yoshioka, Y. , & Ochiya, T. (2018). Biocompatibility of highly purified bovine milk‐derived extracellular vesicles. Journal of Extracellular Vesicles, 7(1), 1440132. 10.1080/20013078.2018.1440132 29511463 PMC5827637

[jex2149-bib-0057] Sverdlov, E. D. (2012). Amedeo Avogadro's cry: What is 1 µg of exosomes? BioEssays, 34(10), 873–875. 10.1002/bies.201200045 22815202

[jex2149-bib-0058] Tan, X.‐H. , Fang, D. , Xu, Y.‐D. , Nan, T.‐G. , Song, W.‐P. , Gu, Y.‐Y. , Gu, S.‐J. , Yuan, Y.‐M. , Xin, Z.‐C. , Zhou, L.‐Q. , Guan, R.‐L. , & Li, X.‐S. (2021). Skimmed bovine milk‐derived extracellular vesicles isolated via “Salting‐Out”: Characterizations and potential functions as nanocarriers. Frontiers in Nutrition, 8, 769223. 10.3389/fnut.2021.769223 34778348 PMC8582325

[jex2149-bib-0059] Théry, C. , Witwer, K. W. , Aikawa, E. , Alcaraz, M. J. , Anderson, J. D. , Andriantsitohaina, R. , Antoniou, A. , Arab, T. , Archer, F. , Atkin‐Smith, G. K. , Ayre, D. C. , Bach, J. M. , Bachurski, D. , Baharvand, H. , Balaj, L. , Baldacchino, S. , Bauer, N. N. , Baxter, A. A. , Bebawy, M. , … Zuba‐Surma, E. K. (2018). Minimal information for studies of extracellular vesicles 2018 (MISEV2018): A position statement of the International Society for Extracellular Vesicles and update of the MISEV2014 guidelines. Journal of Extracellular Vesicles, 7(1), 1535750. 10.1080/20013078.2018.1535750 30637094 PMC6322352

[jex2149-bib-0060] Tong, L. , Hao, H. , Zhang, X. , Zhang, Z. , Lv, Y. , Zhang, L. , & Yi, H. (2020). Oral administration of bovine milk‐derived extracellular vesicles alters the gut microbiota and enhances intestinal immunity in mice. Molecular Nutrition & Food Research, 64(8), e1901251. 10.1002/mnfr.201901251 32180343

[jex2149-bib-0061] Uniacke‐Lowe, T. , & Fox, P. F. (2011). Milk | Equid Milk. In Encyclopedia of dairy sciences. Elsevier, pp. 518–529. 10.1016/B978-0-12-374407-4.00318-6

[jex2149-bib-0062] van Herwijnen, M. J. C. , Zonneveld, M. I. , Goerdayal, S. , Nolte – ’t Hoen, E. N. M. , Garssen, J. , Stahl, B. , Maarten Altelaar, A. F. , Redegeld, F. A. , & Wauben, M. H. M. (2016). Comprehensive proteomic analysis of human milk‐derived extracellular vesicles unveils a novel functional proteome distinct from other milk components. Molecular & Cellular Proteomics, 15(11), 3412–3423. 10.1074/mcp.m116.060426 27601599 PMC5098039

[jex2149-bib-0063] van Niel, G. , D'Angelo, G. , & Raposo, G. (2018). Shedding light on the cell biology of extracellular vesicles. Nature Reviews Molecular Cell Biology, 19(4), 213–228. 10.1038/nrm.2017.125 29339798

[jex2149-bib-0064] Vaswani, K. , Koh, Y. Q. , Almughlliq, F. B. , Peiris, H. N. , & Mitchell, M. D. (2017). A method for the isolation and enrichment of purified bovine milk exosomes. Reproductive Biology, 17(4), 341–348. 10.1016/j.repbio.2017.09.007 29030127

[jex2149-bib-0065] Wiedensohler, A. (1988). An approximation of the bipolar charge distribution for particles in the submicron size range. Journal of Aerosol Science, 19(3), 387–389. 10.1016/0021-8502(88)90278-9

[jex2149-bib-0066] Wiedensohler, A. , Birmili, W. , Nowak, A. , Sonntag, A. , Weinhold, K. , Merkel, M. , Wehner, B. , Tuch, T. , Pfeifer, S. , Fiebig, M. , Fjäraa, A. M. , Asmi, E. , Sellegri, K. , Depuy, R. , Venzac, H. , Villani, P. , Laj, P. , Aalto, P. , Ogren, J. A. , … Bastian, S. (2012). Mobility particle size spectrometers: Harmonization of technical standards and data structure to facilitate high quality long‐term observations of atmospheric particle number size distributions. Atmospheric Measurement Techniques, 5(3), 657–685. 10.5194/amt-5-657-2012

[jex2149-bib-0067] Willms, E. , Johansson, H. J. , Mäger, I. , Lee, Y. , Blomberg, K. E. , Sadik, M. , Alaarg, A. , Smith, C. I. , Lehtiö, J. , El Andaloussi, S. , Wood, M. J. , & Vader, P. (2016). Cells release subpopulations of exosomes with distinct molecular and biological properties. Scientific Reports, 6, 22519. 10.1038/srep22519 26931825 PMC4773763

[jex2149-bib-0068] Willms, E. , Cabañas, C. , Mäger, I. , Wood, M. J. A. , & Vader, P. (2018). Extracellular vesicle heterogeneity: subpopulations, isolation techniques, and diverse functions in cancer progression. Frontiers in Immunology, 9, 738. 10.3389/fimmu.2018.00738 29760691 PMC5936763

[jex2149-bib-0069] Wolf, T. , Baier, S. R. , & Zempleni, J. (2015). The intestinal transport of bovine milk exosomes is mediated by endocytosis in human colon carcinoma Caco‐2 cells and rat small intestinal IEC‐6 cells. The Journal of Nutrition, 145(10), 2201–2206. 10.3945/jn.115.218586 26269243 PMC4580964

[jex2149-bib-0070] Yamauchi, M. , Shimizu, K. , Rahman, M. , Ishikawa, H. , Takase, H. , Ugawa, S. , Okada, A. , & Inoshima, Y. (2019). Efficient method for isolation of exosomes from raw bovine milk. Drug Development and Industrial Pharmacy, 45(3), 359–364. 10.1080/03639045.2018.1539743 30366501

[jex2149-bib-0071] Zhong, J. , Xia, B. , Shan, S. , Zheng, A. , Zhang, S. , Chen, J. , & Liang, X. J. (2021). High‐quality milk exosomes as oral drug delivery system. Biomaterials, 277, 121126. 10.1016/j.biomaterials.2021.121126 34544033

