# Supplementary material for: Comparative analysis of the effects of different purification methods on the yield and purity of cow milk extracellular vesicles
Source: J Extracell Biol. 2024 Apr 22;3(4):e149. doi: 10.1002/jex2.149 (PMC11080921; doi:10.1002/jex2.149)
Supplement: Supplementary file 1 — Supporting Information [file JEX2-3-e149-s001.docx]

**Supplementary table T1.** The most important control and EV samples and the corresponding analysis performed on each sample.

| **Analysis/ Sample** | **SEC** | **NTA** | **DMPS** | **TEM** | **WB** | **RNA extraction/ Bioanalyzer** | **Proteomics** | **Lipidomics** |
| --- | --- | --- | --- | --- | --- | --- | --- | --- |
| **Milk** | **X** | **X** |  |  | **X** | **X** |  |  |
| **UC** | **X** | **X** |  | **X** | **X** | **X** | **X** |  |
| **UC SEC** | **X** | **X** |  | **X** | **X** | **X** | **X** |  |
| **UC GRAD** | **X** | **X** |  | **X** | **X** | **X** | **X** |  |
| **UC GS** | **X** | **X** |  | **X** |  |  | **X** |  |
| **17h UC pellet** |  | **X** |  | **X** | **X** | **X** |  |  |
| **AA** | **X** | **X** | **X** | **X** | **X** | **X** | **X** | **X** |
| **AA SEC** | **X** | **X** | **X** | **X** | **X** | **X** | **X** | **X** |
| **AA GRAD** | **X** | **X** | **X** | **X** | **X** | **X** | **X** | **X** |
| **AA GS** | **X** | **X** | **X** | **X** |  |  | **X** | **X** |
| **17h AA pellet** |  | **X** |  | **X** | **X** | **X** |  |  |

**Supplementary table T2.** RNA concentration from TERI-isolated samples along with protein concentration from DC assay (Biorad). EV fractions from the centrifugation are UC (without acetic acid treatment) and AA (with acetic acid treatment) with additional purifications described as UC SEC and AA SEC (size exclusion chromatography) or UC/AA GRAD (sucrose density gradient centrifugation). 17h UC and 17h AA are the pellets derived from 17h ultracentrifugation of the supernatant after EV pelleting for samples UC and AA.

|  | **RNA ng/µl Nanodrop** | | | **RNA ng/µl Qubit** | | | **Protein µg/µl** | | |
| --- | --- | --- | --- | --- | --- | --- | --- | --- | --- |
| **Sample/Isolation** | **1** | **2** | **3** | **1** | **2** | **3** | **1** | **2** | **3** |
| **UC** | 13. 8 | 17.1 | 10.1 | 3.74 | 5.80 | 3.12 | 9.80 | 11.3 | 6.75 |
| **UC SEC** | 4.90 | 4.10 | 1.20 | 1.16 | 0.51 | 0.26 | 0.88 | 1.12 | 0.35 |
| **UC GRAD** | 13.3 | 9.70 | 8.40 | 1.45 | 2.46 | 0.82 | 1.46 | 0.68 | 0.48 |
| **AA** | 12.5 | 18.4 | 13.6 | 3.68 | 10.8 | 4.06 | 6.30 | 6.20 | 5.19 |
| **AA SEC** | 9.80 | 3.00 | 5.60 | 2.66 | 0.41 | 0.48 | 1.26 | 0.29 | 0.33 |
| **AA GRAD** | 4.90 | 8.40 | 14.3 | 1.23 | 3.12 | 2.92 | 1.03 | 1.02 | 1.69 |
| **Controls** | **RNA ng/µl Nanodrop** | **RNA ng/µl Qubit** | **Protein µg/µl** |  |  |  |  |  |  |
| Milk | 4.9 | 0.88 | 59.33 |  |  |  |  |  |  |
| 17h UC | 1.7 | 0.23 | 11.69 |  |  |  |  |  |  |
| 17h AA | 0.5 | 0.13 | 7.00 |  |  |  |  |  |  |


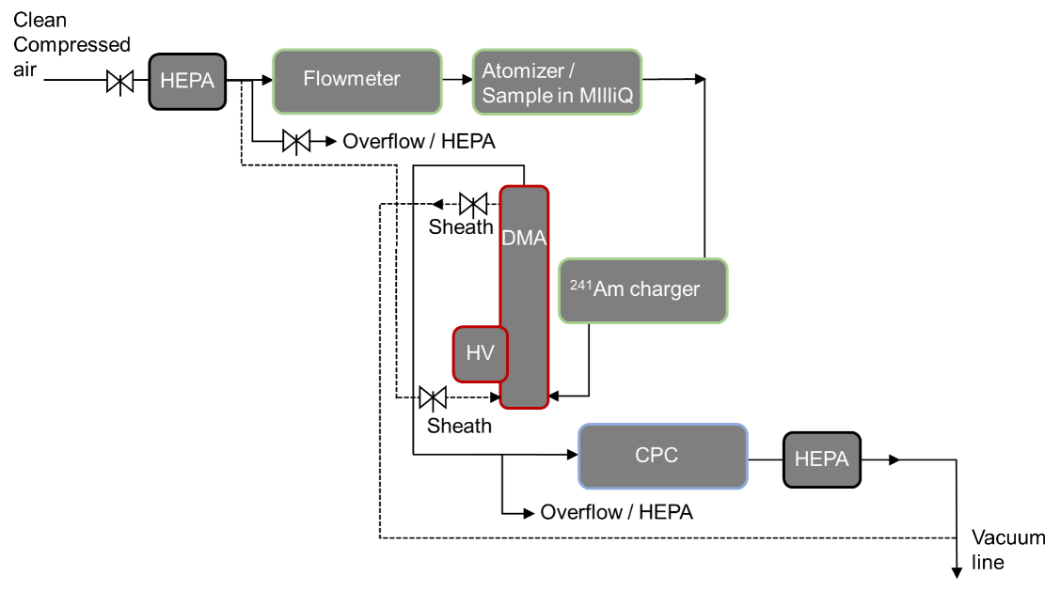


**Supplementary figure S1** Aerosol measurement setup for the EV size-distribution studies.

**
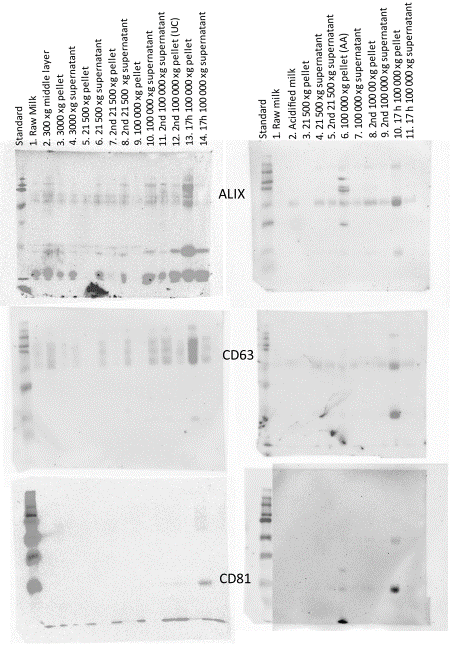
**

**Supplementary figure S2**  Complete images of western blot membranes from isolation process. Left panels were samples taken from EV-enrichment process. Right panels were samples from additional acetic acid treatment of EV-enrichment process. Primary antiobdies were rabbit anti-ALIX 1:2500 (cell signaling, (E6P9B) Rabbit mAb #92880), mouse anti-CD81 1:1000 (Santa Cruz biotechnologies, (B-11): sc-166029), and mouse anti-bovine CD63 1:500 (BioRad, MCA2042GA) Western C (BioRad) was used as standard. CD-81 blot of AA-samples had the standard measured separately to prevent overexposure.


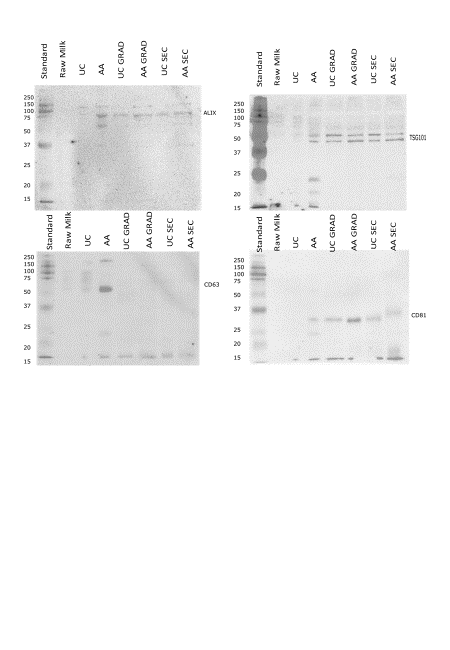


**Supplementary figure S3** Complete images of western blot membranes from EV-fractions; ultracentrifugation isolated EVs (UC), UC coupled with sucrose density gradient centrifugation (UC GRAD), UC coupled with size exclusion chromatograpgy (UC SEC) and their acetic acid treated counterparts (AA, AA GRAD and AA SEC). Primary antiobdies were rabbit anti-ALIX 1:2500 (Cell Signaling, (E6P9B) Rabbit mAb #92880), mouse anti-CD81 1:1000 (Santa Cruz biotechnologies, (B-11): sc-166029), mouse anti-bovine CD63 1:500 (BioRad, MCA2042GA) and rabbit anti-TSG101 1:2500 (Nordic BioSite, ABB-709, ABB-OTVG5L-100).


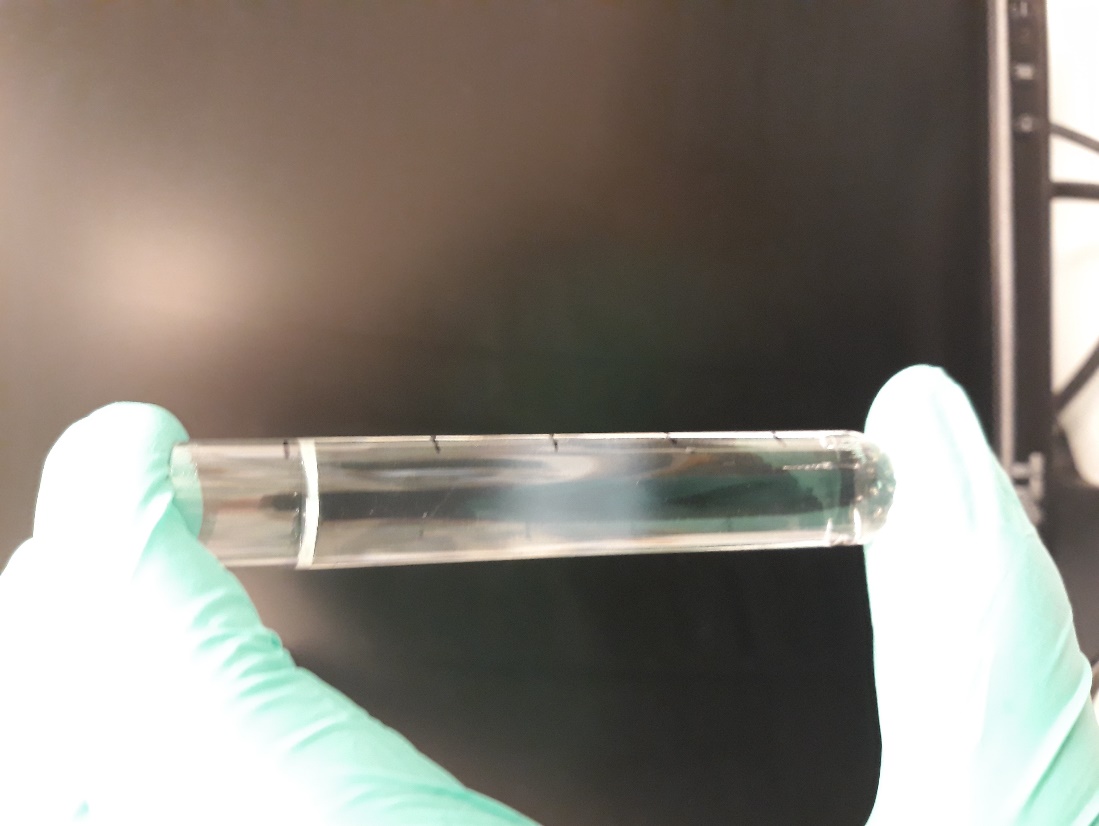


**Supplementary figure S4** Milk EV-fractions visible as white cloudy layer in sucrose gradient.


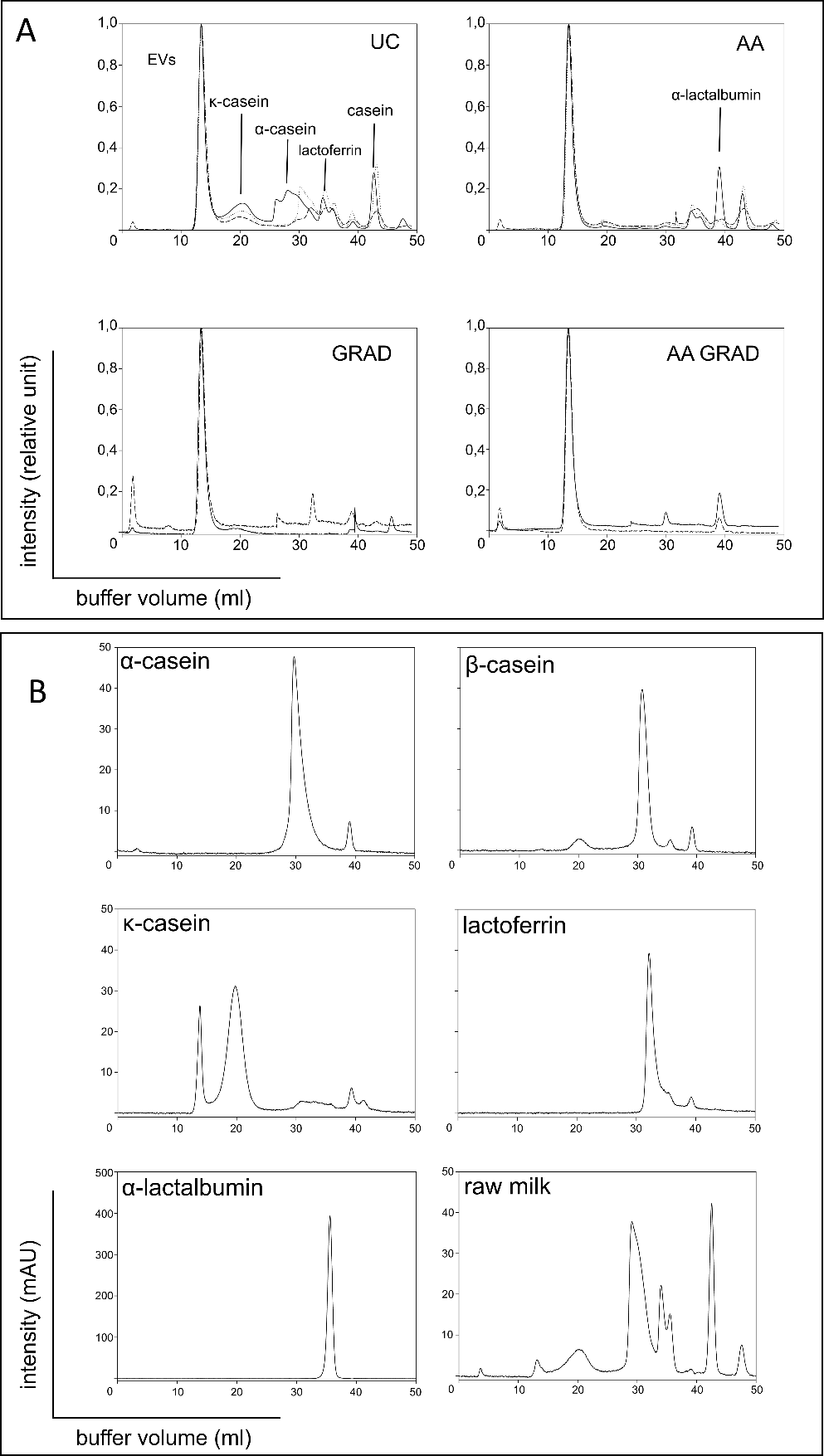


**Supplementary figure S5** Size exclusion chromatograms from EV samples: (A) Ultracentrifugation-isolated EVs (UC), UC coupled with sucrose density gradient centrifugation (UC GRAD) and their acetic acid treated counterparts (AA and AA GRAD). EVs are eluting in column void volume peak 13-15 ml. Protein standards (B) were analyzed to identify separated proteins from EV-samples. (α-casein 24 kDa, β-casein 25 kDa, κ-casein 21 kDa, lactoferrin 78 kDa, α-lactalbumin 16 kDa).

**
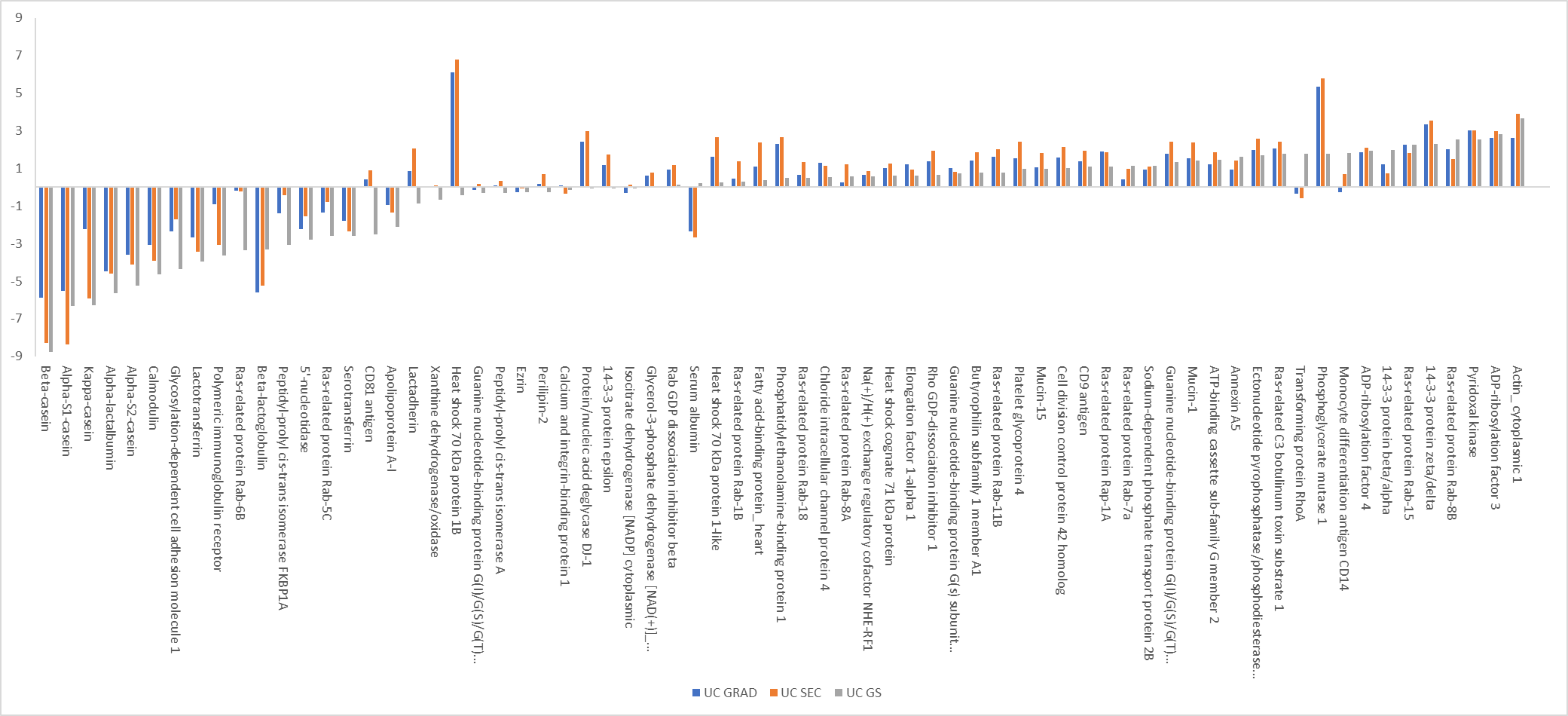
 Supplementary figure S6** Statistically different proteins from UC-isolation samples compared to UC-sample set at 0 abundancy. UC-sample was further purified with gradient centrifugation (UC GRAD), size exclusion chromatography (UC SEC) or combination of both methods (UC GS).


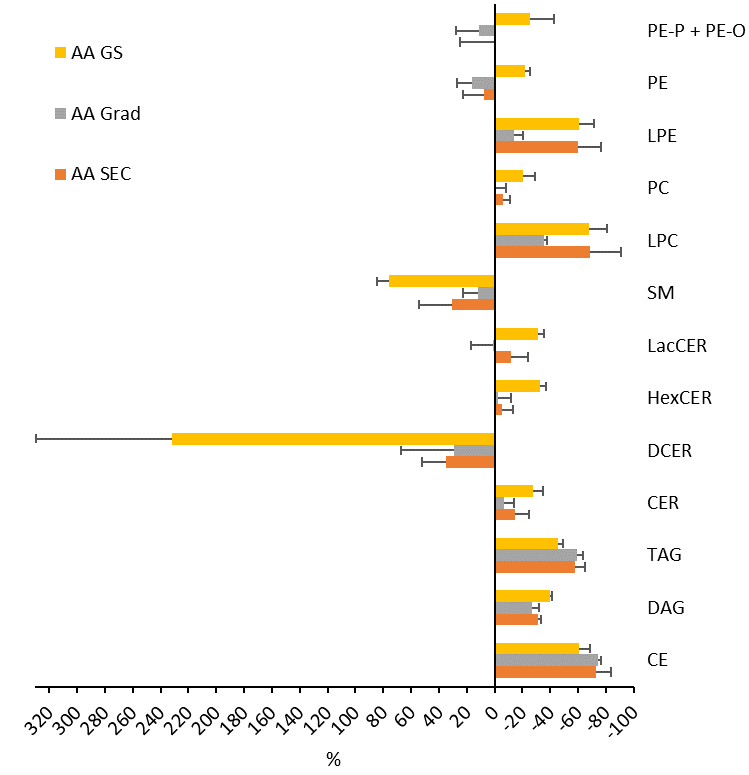


**Supplementary figure S7.** Relative compositional differences in mole fractions (*n*/*n* ± SD) of lipid classes between acetic acid-treated ultracentrifuged sample (AA) and samples purified with size-exclusion chromatography (AA SEC), gradient centrifugation (AA GRAD), and with both methods (AA GS) with three biological replicates. AA sample is set at 0 abundancy. CE, cholesteryl ester; CER, ceramide; DAG, diacylglycerol; DCER, dihydroceramide; FFA, free fatty acid; HexCER, hexosylceramide; LacCER, lactosylceramide; LPC, lysophosphatidylcholine; LPE, lysophosphatidylethanolamine; PC, phosphatidylcholine; PE-O & PE-P, ether-linked PE; PE, phosphatidylethanolamine; SM, sphingomyelins; TAG, triacylglycerol.


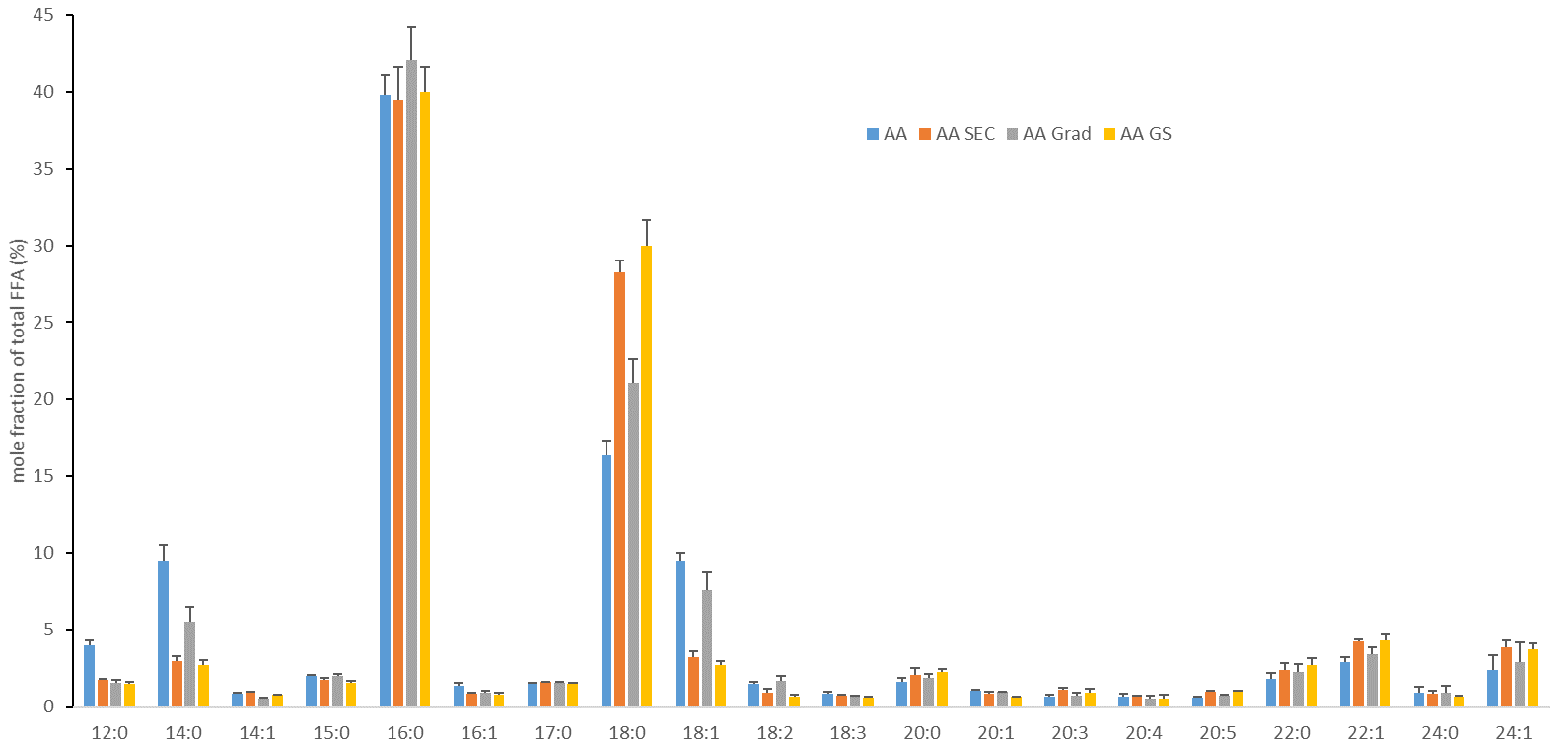


**Supplementary figure S8.** Relative mole fractions of free fatty acids (FFA; ± SD) in acetic acid-treated ultracentrifuged sample (AA) and samples purified with size-exclusion chromatography (AA SEC), gradient centrifugation (AA GRAD), and with both methods (AA GS) with three biological replicates.


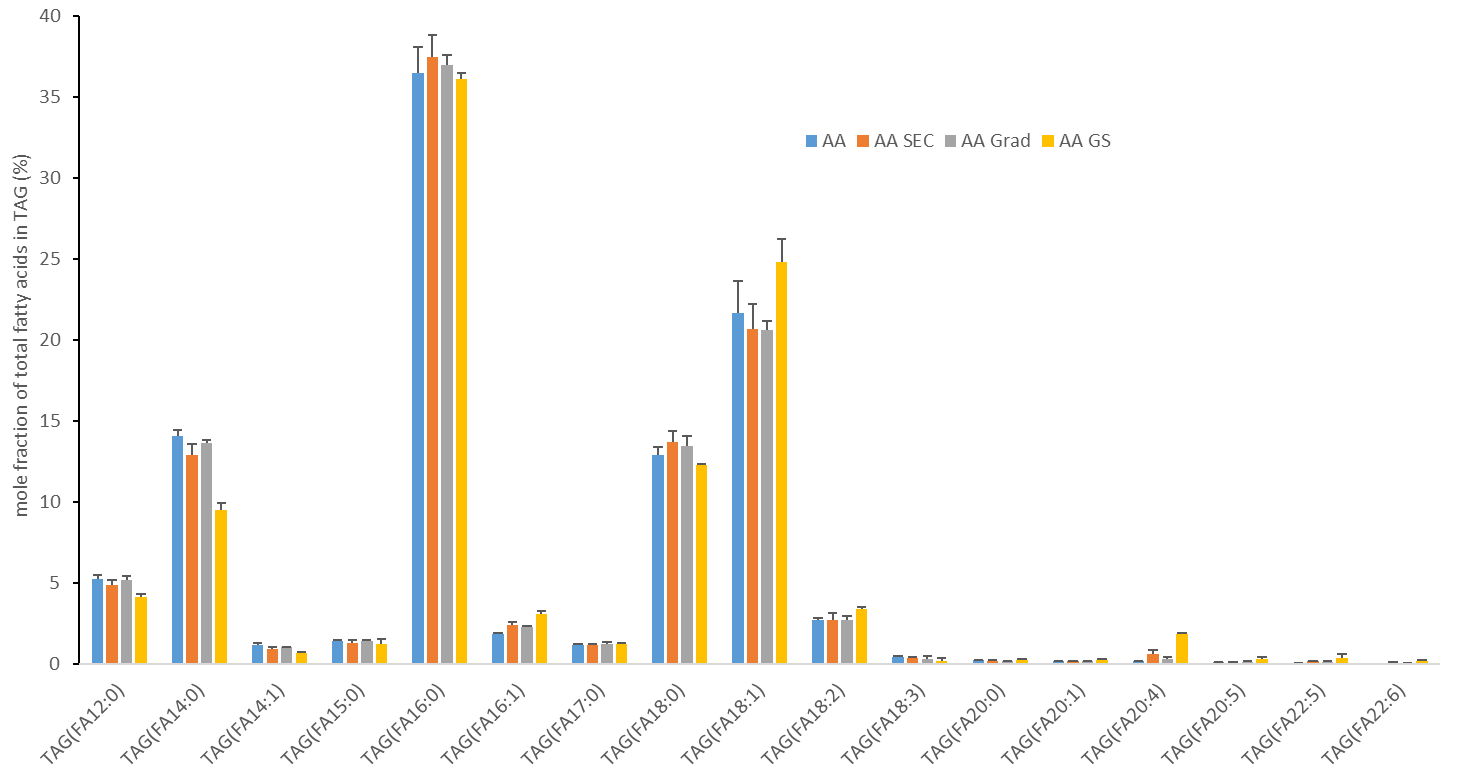


**Supplementary figure S9.** Relative mole fractions of total fatty acids in triacylglycerols (TAG; ± SD) in acetic acid-treated ultracentrifuged sample (AA) and samples purified with size-exclusion chromatography (AA SEC), gradient centrifugation (AA GRAD), and with both methods (AA GS) with three biological replicates.


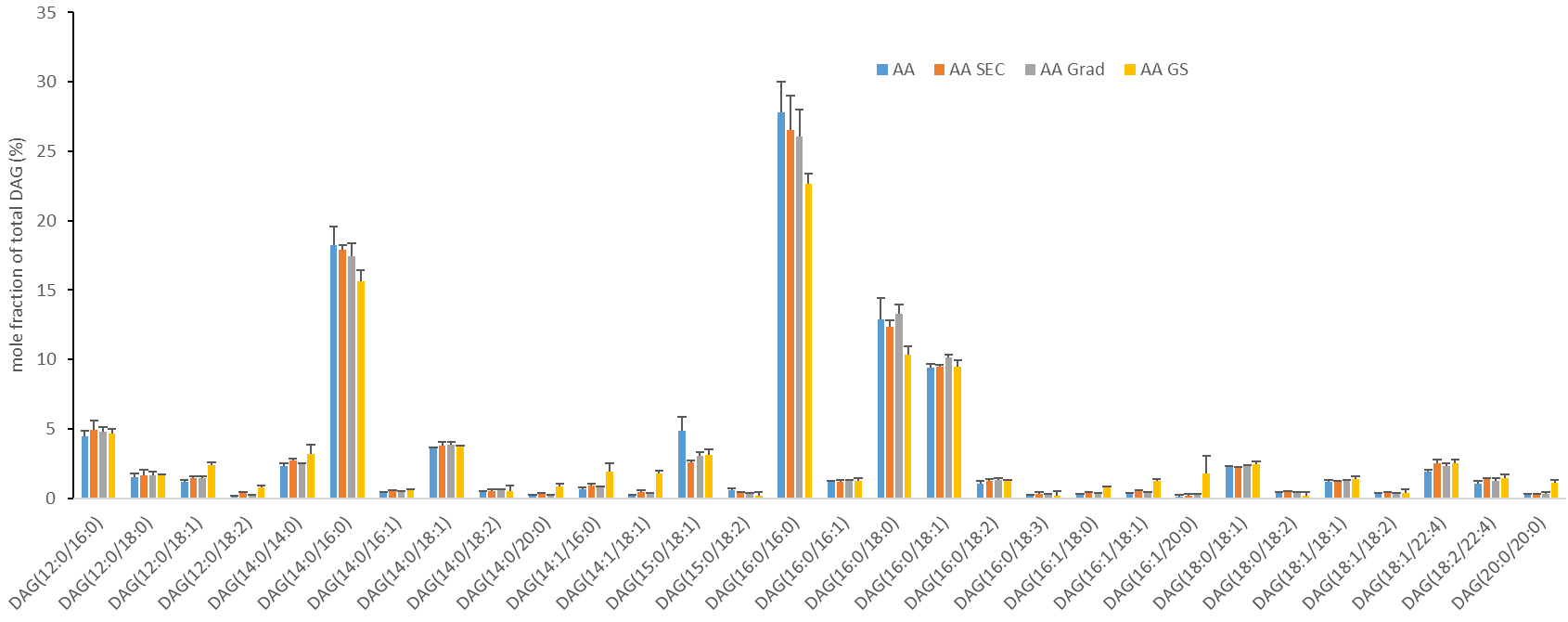


**Supplementary figure S10.** Relative mole fractions of diacylglycerols (DAG) species (± SD) in acetic acid-treated ultracentrifuged sample (AA) and samples purified with size-exclusion chromatography (AA SEC), gradient centrifugation (AA GRAD), and with both methods (AA GS) with three biological replicates.


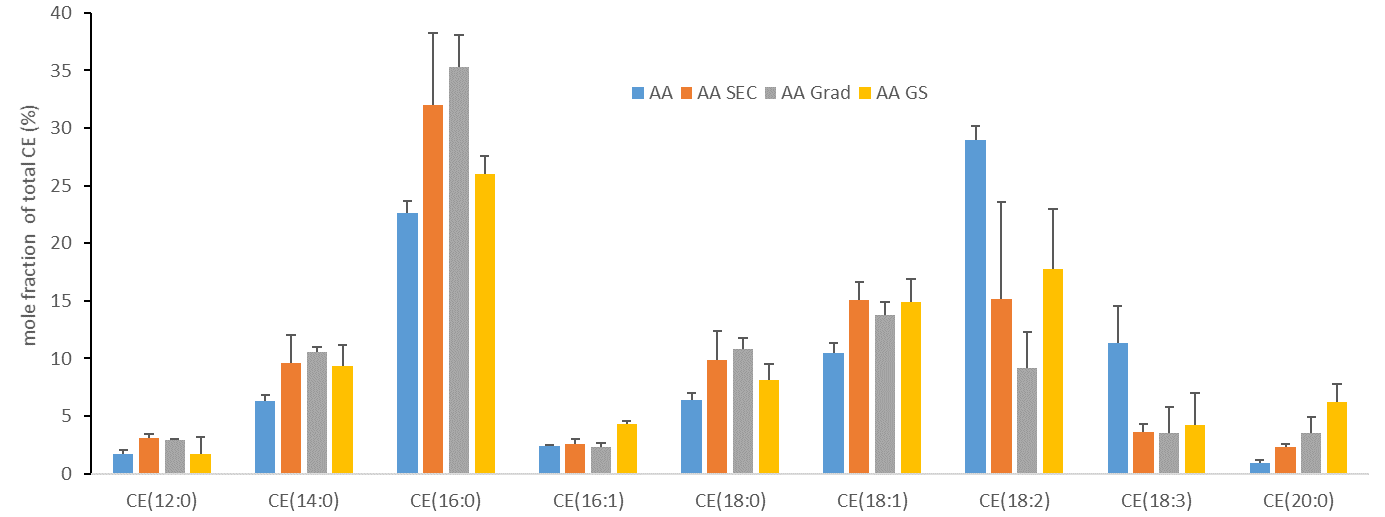


**Supplementary figure S11.** Relative mole fractions of cholesterol ester (CE) species (± SD) in acetic acid-treated ultracentrifuged sample (AA) and samples purified with size-exclusion chromatography (AA SEC), gradient centrifugation (AA GRAD), and with both methods (AA GS) with three biological replicates.

**
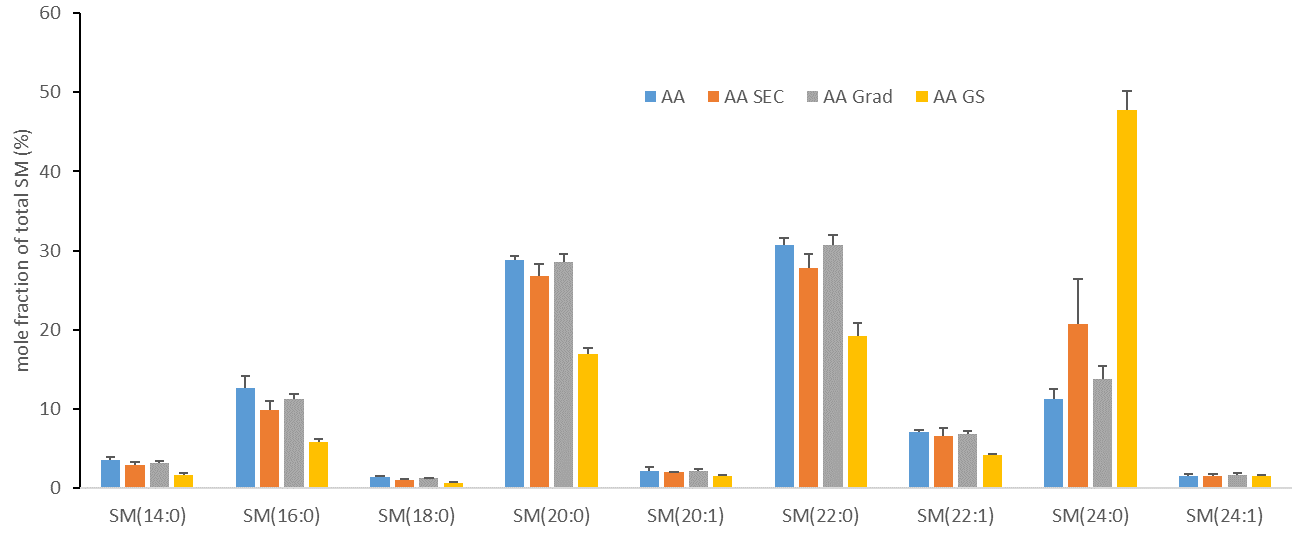
**

**Supplementary figure S12.** Relative mole fractions of sphingomyelin (SM) species (± SD) in acetic acid-treated ultracentrifuged sample (AA) and samples purified with size-exclusion chromatography (AA SEC), gradient centrifugation (AA GRAD), and with both methods (AA GS) with three biological replicates.


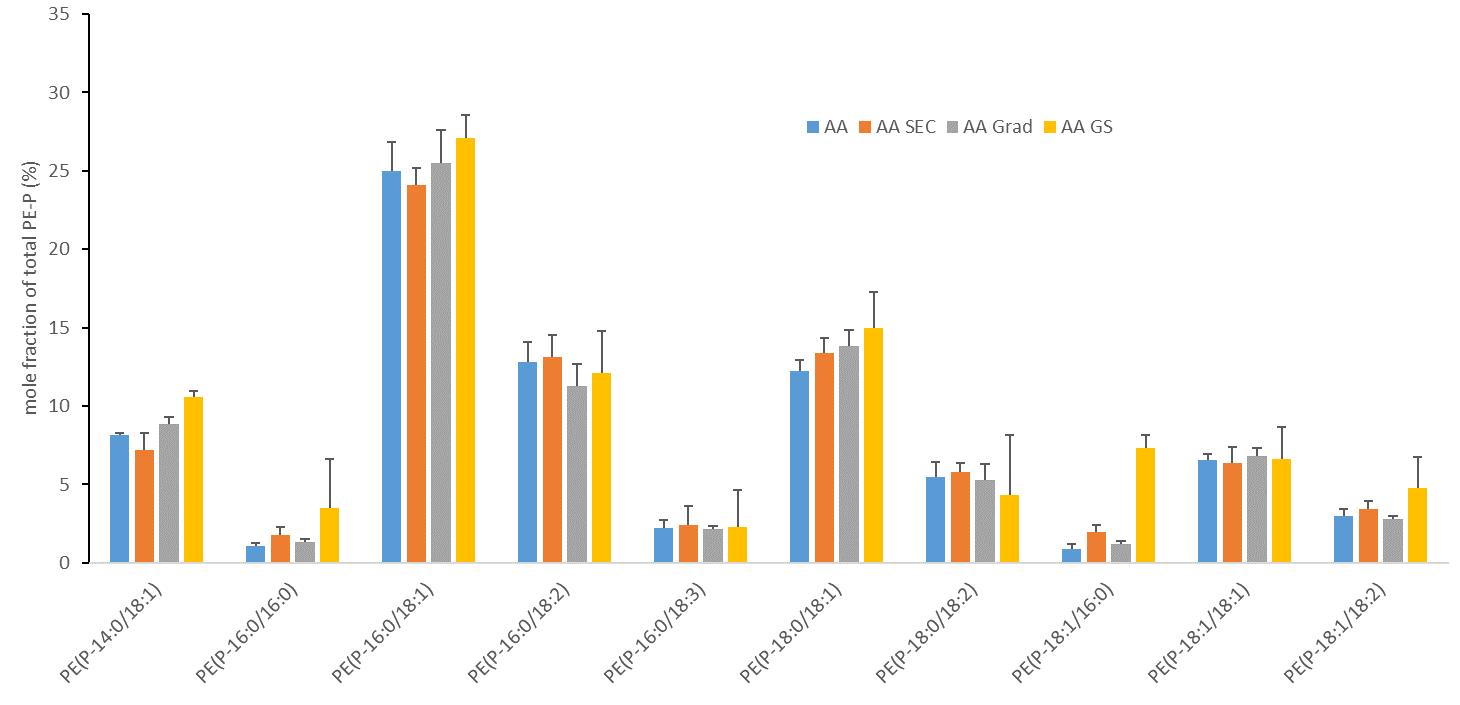


**Supplementary figure S13.** Relative mole fractions of ether-linked phosphatidylethanolamine (PE-P) species (± SD) in acetic acid-treated ultracentrifuged sample (AA) and samples purified with size-exclusion chromatography (AA SEC), gradient centrifugation (AA GRAD), and with both methods (AA GS) with three biological replicates.


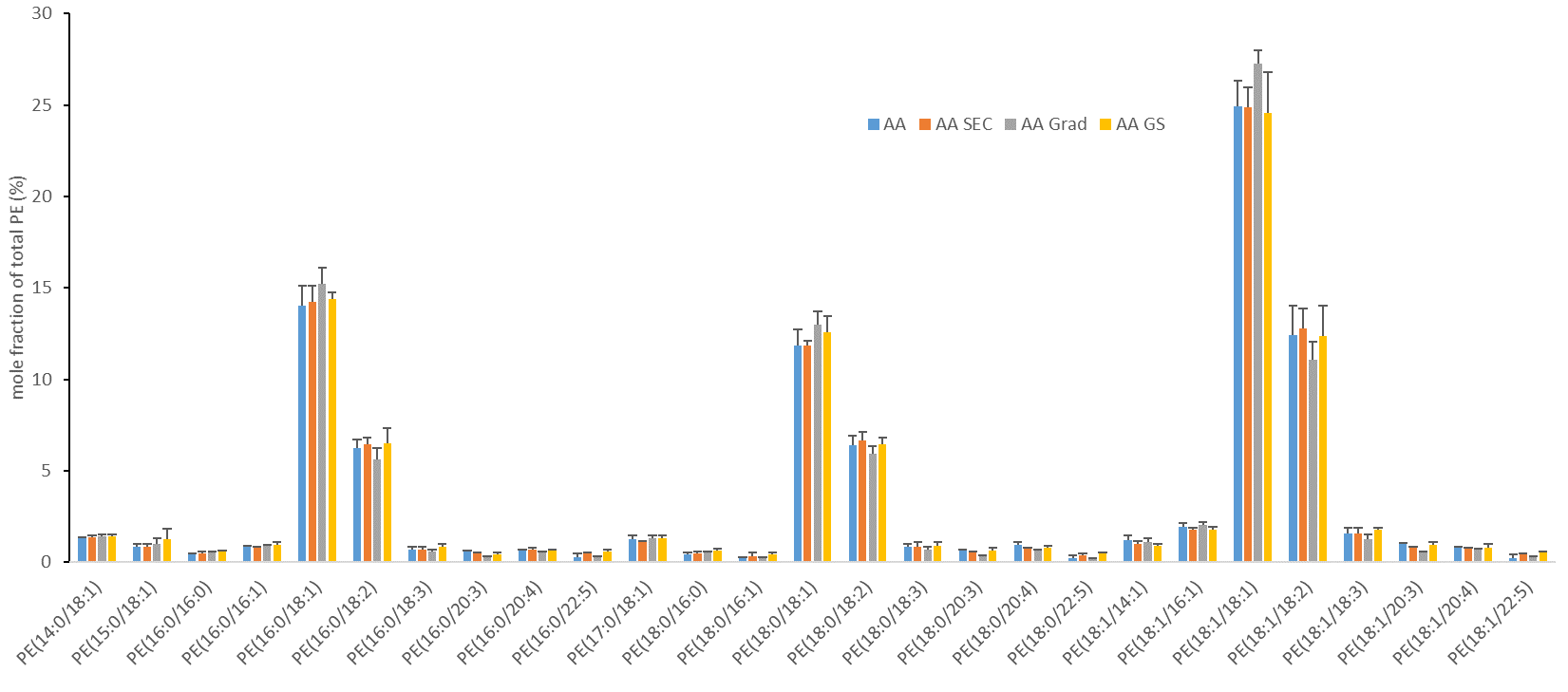


**Supplementary figure S14.** Relative mole fractions of phosphatidylethanolamine (PE) species (± SD) in acetic acid-treated ultracentrifuged sample (AA) and samples purified with size-exclusion chromatography (AA SEC), gradient centrifugation (AA GRAD), and with both methods (AA GS) with three biological replicates.


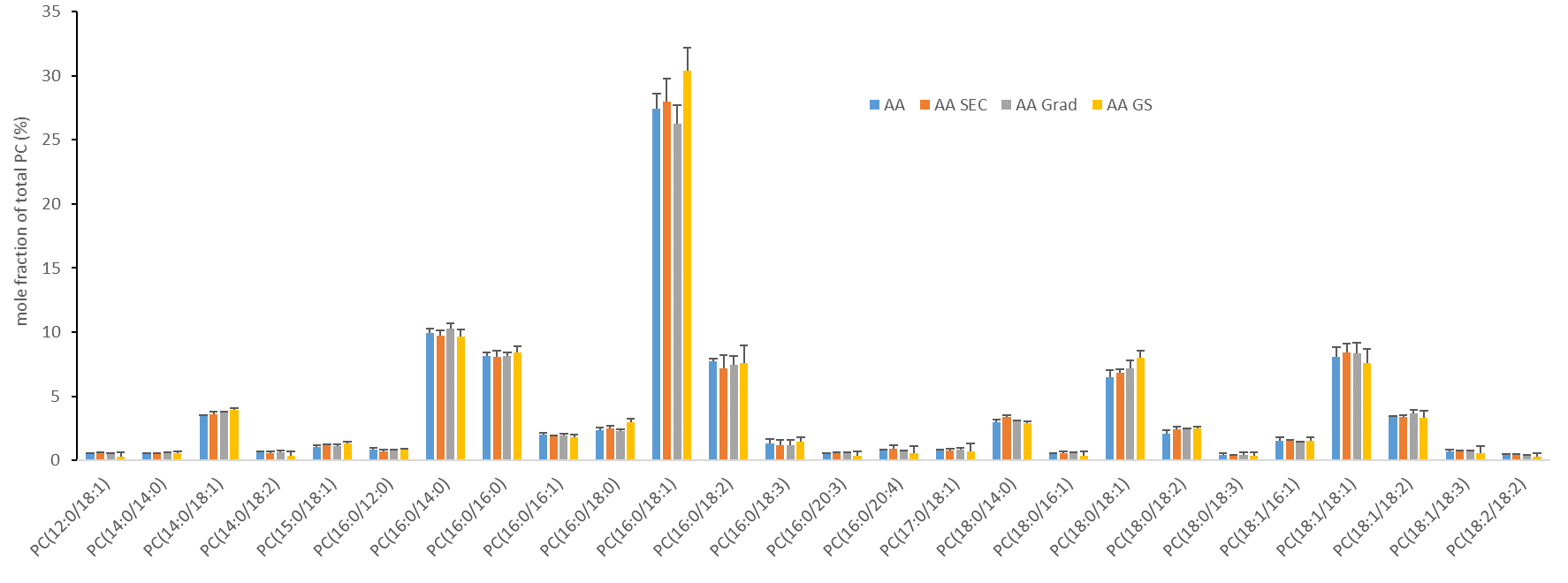


**Supplementary figure S15.** Relative mole fractions of phosphatidylcholine (PC) species (± SD) in acetic acid-treated ultracentrifuged sample (AA) and samples purified with size-exclusion chromatography (AA SEC), gradient centrifugation (AA GRAD), and with both methods (AA GS) with three biological replicates.





**Supplementary figure S16** TEM image of EV-sample UC isolated with ultracentrifugation.





**Supplementary figure S17** TEM image of EV sample UC GRAD, UC further purified with sucrose density gradient centrifugation.





**Supplementary figure S18** TEM image of EV sample UC SEC, UC further purified with size exclusion chromatography.





**Supplementary figure S19** TEM image of EV sample UC GS, UC further purified with sucrose gradient centrifugation and size exclusion chromatography.





**Supplementary figure S20** TEM of pellet from 17h ultracentrifugation of UC-supernatant.





**Supplementary figure S21** TEM image of EV-sample AA, isolated with acetic acid precipitation coupled ultracentrifugation.





**Supplementary figure S22** TEM image of EV sample AA GRAD, AA further purified with sucrose density gradient centrifugation.





**Supplementary figure S23**

TEM image of EV sample AA SEC, AA further purified with size exclusion chromatography.





**Supplementary figure S24**

TEM image of EV sample AA GS, AA further purified with sucrose gradient centrifugation and size exclusion chromatography.





**Supplementary figure S25** TEM of pellet from 17h ultracentrifugation of AA-supernatant.
