# Supplementary material for: Comparative analysis of the effects of different purification methods on the yield and purity of cow milk extracellular vesicles
Source: J Extracell Biol. 2024 Apr 22;3(4):e149. doi: 10.1002/jex2.149 (PMC11080921; doi:10.1002/jex2.149)
Supplement: Supplementary file 2 — Supporting Information [file JEX2-3-e149-s002.docx]

| ID | NAME | Function in bovine | Function in human | Identity |
| --- | --- | --- | --- | --- |
| A0JNI8 | LIM/homeobox protein Lhx9 | *Involved in gonadal development | Involved in gonadal development; repression will lead to decrease in Testosterone production in Leydig cells(1) | 98,2 |
| A2VDP0 | Ashwin | *Embryonic morphogenesis | May participate in RNA repair as a part of ligation complexes. (2) | 89,2 |
| A4FV54 | Ras-related protein Rab-8A | *Key regulators of intracellular trafficking. See entries Q3T0F5 and Q2HJH2, takes part in intracellular membrane trafficking, member of RAS oncogene family. | Interacts with myosin proteins to facilitate intracellular trafficking. Functions in the exocytic pathway.  Related to control of autophagy. (3-5) | 99,5 |
| A4FV72 | Peptidyl-prolyl cis-trans isomerase... | *Involved in pre-mRNA processing as part of the spliceosome. | Involved in spliceosomes with pre-mRNA processing and binding of mRNA. | 98,0 |
| A4IF97 | Myosin regulatory light chain 12B | *Regulatory subunit of myosin plays an important part in smooth muscle and nonmuscle cell contractions via its phosphorylation. Binds calcium. | Manages the stability of myosin 2, that plays a central role in cell adhesion, migration and division. (6) | 97,7 |
| A4IFU8 | Aurora kinase A and ninein-interacting protein | *DNA binding protein that accumulates at double-strand breaks at DNA. Promotes DNA resection and homologous recombination. Recruits RBBP8 and CtIP to DSBs. | Binds to double-strand breaks in DNA; recruits RBBP8 and CtIP with contacting them. Guides the DNA repair to homologous recombination. (7) | 68,0 |
| A5PJU8 | Protein LBH | *Trascriptional activator | Activates the activator protein-1 and serum response element derived signaling pathway MAPK (8) | 93,3 |
| A5PKE4 | Transcription elongation factor A N... | *Transcription; elongation of DNA-templated transcription | Prenatal mercury concentration influences the DNA methylation at TCEANC2 genes. Differences in expression were seen associated with diabetes in mice models. (9, 10) | 91,3 |
| A6H7C9 | Centrosomal protein of 19 kDa | *Protein required for ciliation. Interacts with RABL2B and recruits ciliary vesicles. | Interaction with RABL2B greatly enhances the rate of ciliation. Initiates the intraflagellar transport (11) | 93,9 |
| A6QLK6 | GRB2-related adapter protein | *Couples signals from receptor and cytoplasmic tyrosine kinases to the Ras signaling pathway. | Mutation of GRAP leads to deafness in humans most likely via defects in GRAP interactions. (12) | 91,2 |
| A6QPE1 | RNA-binding protein 48 | *Binds RNA | *Binds RNA, Defects could be associated with renal ciliopathies. (13) | 75,7 |
| A6QQ68 | Protein FAM228B | *One of 7 annotated and reviewed FAM288 proteins (2 in humans, 2 in bovine, 2 in mouse and 1 in rat, checked 21.04.2020) |  | 77,6 |
| A6QR46 | Ras-related protein Rab-6B | *Appears to take part in retrograde membrane traffic at Golgi complex. | One of the factors in retrograde transport in neuronal cells with interaction with bicaudal-D1; transport with dynein/dynactin complexes. (14) | 100,0 |
| O18963 | Cytochrome P450 2E1 | *Involved in metabolism of fatty acids; transfer of molecular oxygen to the substrate to reduce another oxygen to water. | Metabolism of fatty acids; highest affinity for lauric acids. Has affinity for unsaturated fatty acids. (15) | 78,1 |
| O46414 | Ferritin heavy chain | *Stores iron as soluble and non-toxic form. Has ferroxidase activity. | *Similar function as bovine homolog. | 91,2 |
| O77834 | Peroxiredoxin-6 | Functions as thiol-specific peroxidase. Can function without 2+ ions. (16, 17) | Thiol-specific peroxidase; has one conserved cysteine in reactive site. (Sang Won Kang, Ivan C. Baines et al. 1998) | 95,1 |
| P00711 | Alpha-lactalbumin | *Regulatory subunit of lactose synthase. | *Regulatory subunit of lactose synthase. | 73,9 |
| P01190 | Pro-opiomelanocortin | *Growth factor with multiple effects. Specific enzymatic cleavages yield different active peptides. | *Similar function as with bovine homolog. | 80,3 |
| P02070 | Hemoglobin subunit beta | *Involved in oxygen transport.  Has inhibitory activity against enkephalin-degrading enzymes.(18) | *Involved in oxygen transport | 84,8 |
| P02638 | Protein S100-B | Calsium and zinc binding and modulating interacting proteins by said ion binding. (19, 20) | *Member of Ca^2+^ binding EF-hand protein family. Can modulate multiple proteins in interactions, such as p53 and mitochondrial ATAD3A. | 96,7 |
| P02662 | Alpha-S1-casein | *Major protein component of bovine milk. | *Similar function as bovine homolog. Low similarity. | 33,0 |
| P02663 | Alpha-S2-casein | *Major protein component of bovine milk. | Not present in humans. |  |
| P02666 | Beta-casein | *Major protein component of bovine milk. Important role in the determination of casein micelle surface properties. | *Similar function as bovine homolog. | 56,4 |
| P02668 | Kappa-casein | *Major protein component of bovine milk. Stabilizes milk casein micelles. Has multiple bioactive peptides when proteolytically cleaved. | *Similar function as bovine homolog. | 53,1 |
| P02690 | Myelin P2 protein | *May play a role in lipid transport in Schwann cells. May bind cholesterol. | *May play a role in lipid transport in Schwann cells. May bind cholesterol (21) | 92,4 |
| P02721 | ATP synthase-coupling factor 6, mit... | Component of ATP synthase complex. Located in mitochondria. (22) | *Mitochondrional membrane ATP synthase. | 80,6 |
| P02754 | Beta-lactoglobulin | *Allergenic properties in humans, binds retinol. | *No homolog found in humans; closest similarity protein is glycodelin. | 44,4 |
| P02769 | Serum albumin | *Most abundant serum protein with great transport capability for ions, hormones, bilirubin and drug molecules. | *As with bovine, transport capability for majority of Zn in plasma. (23, 24) | 76,5 |
| P03518 | Envelopment polyprotein | *Viral protein from Rift valley fever virus. Virus can infect, bovine, goats, sheep and humans. |  |  |
| P04272 | Annexin A2 | *Phospholipid binding protein regulated by calcium. | Regulates endogenous LDL receptor levels, and can therefore induce the reduced intake of LDL. (25) | 98,5 |
| P04695 | Guanine nucleotide-binding protein ... | Functions as signal transducer for Rho photoreceptor in rod cells.(26-28) | Defect in GNAT1 can cause stationary night blindness. (Naeem, Chavali et al. 2012) | 99,1 |
| P04696 | Guanine nucleotide-binding protein ... | Signal transducer from G-protein transducing in from rhodopsin in rod cells. (29) | *Tranducin is an amplifier and one of transducers of the visual impulse that performs the coupling between rhodopsin and cGMP-phosphodiesterase. | 96,6 |
| P04972 | Retinal rod rhodopsin-sensitive cGM... | *Transmission and amplification of visual signal | *Transmission and amplification of visual signal | 97,7 |
| P07107 | Acyl-CoA-binding protein | Binding of fatty acyl –CoA and therefore participation in fatty acid metabolism. (30) | Can bind to GABA receptor and act as an inhibitor along with fatty acid metabolism. (31) | 93,1 |
| P07514 | NADH-cytochrome b5 reductase 3 | *Desaturation and elongation of fatty acids, cholesterol biosynthesis, drug metabolism, and, in erythrocyte, methemoglobin reduction. | Desaturation and elongation of fatty acids, cholesterol biosynthesis, drug metabolism, and, in erythrocyte, methemoglobin reduction. Defects can cause methemoglobinemia.(32, 33)Expressed on ER and mitochondrion membrane, has an membrane anchor. Catalyses the reduction of Fe(III)-cytochrome *b*_5_+ to Fe(II)-cytochrome *b*_5_ with NADH. (Takesue, Omura 1970) | 93,0 |
| P07857 | Non-specific lipid-transfer protein | *Transfers all common phospholipids, cholesterol and gangliosides between membranes *in vitro*. May take part in regulation of steroidogenesis. | Transfer of sterols and phosphatidylcholine. (34) | 86,9 |
| P08239 | Guanine nucleotide-binding protein ... | *G-protein coupled protein with unclear function | *As bovine. Several neurological disorders are associated to mutations in GNAO1 | 99,4 |
| P0C7Q4 | Guanine nucleotide-binding protein ... | *G-protein coupled protein associated in tasting of umami. Highly expressed in sperm cells as chemotaxis receptors. Part of chemosensory in bos Taurus airways. (35-37)) | *Tasting of bitter and sweet in association with G-protein coupled receptors.  Expressed in human intestines, may modulate GLP-1 secretion and can be associated to obesity. (Hyeung-Jin Jang, Zaza Kokrashvili et al. 2007). | 96,3 |
| P0C914 | Overexpressed in colon carcinoma 1 ... | *Homolog to human protein overexpressed in colon carcinoma | Protein expressed in differentiation of menenchymal stem cells. Different expression levels in different tissues (38) | 93,7 |
| P0CB32 | Heat shock 70 kDa protein 1-like | *Molecular chaperone protein protecting proteome from various stress inducers. | Member of HSP-family of intra- and extracellular protein chaperones. (39) | 96,3 |
| P0CG53 | Polyubiquitin-B | *Free or connected to proteins as a mono- or polymer, effects depend on the connection location and level of uibiquitylation. | Exists as free or anchored to proteins. Effects on the targeted protein depend on the linkage of the ubiquitin to the protein. (40) | 99,7 |
| P0CH28 | Polyubiquitin-C | See entry P0CG53 |  | 99,7 |
| P10790 | Fatty acid-binding protein, heart | *Intracellular transport of long-chain fatty acids. Inhibition of mammary carcinoma cells. | FABP are thought to play a role in the intracellular transport of long-chain fatty acids and their acyl-CoA esters.(41) | 88,7 |
| P10948 | Ras-related protein Rab-3B | *Protein and vesicular transport | *Protein and vesicular transport | 97,7 |
| P10949 | Ras-related protein Rab-3C | *Protein and vesicular transport | *Protein and vesicular transport | 99,6 |
| P11017 | Guanine nucleotide-binding protein ... | *Transduction of G-protein coupled receptor induced responses | Signal transduction from G-protein coupled receptor (42) | 100,0 |
| P11023 | Ras-related protein Rab-3A | *Small GTP-binding protein that plays a role in exocytosis and secretion | Exocytosis and vesicle tethering. | 98,636 |
| P12932 | Ankyrin repeat domain-containing pr... | Cowpox derived protein |  |  |
| P13696 | Phosphatidylethanolamine-binding pr... | *Binding of ATP, opioids and phosphatidylethanolamine. Inhibitor of kinase activity of RAF1 | Part of highly conserved PEBP family. Can bind variety of lipids by the phosphate head groups therefore adhering to membranes (43) | 94,1 |
| P15396 | Ectonucleotide pyrophosphatase/phos... | *Hydrolase that metabolizes extracellular nucleotides. Prevents apoptosis in intestines by hydrolyzation of ATP. | Modulates purinergic signaling by degradation of extracellular nucleotides. (44) | 81,8 |
| P15497 | Apolipoprotein A-I | *Participates in reverse transport of cholesterol from tissues to the liver for excretion. | Can be used as a marker for coronary artery disease when measured from serum. Activates CDC42 along with protein kinases in human fibroblasts. (45) | 78,7 |
| P16602 | A-type inclusion protein A25 homolo... | Protein from cowpox virus |  |  |
| P18203 | Peptidyl-prolyl cis-trans isomerase... | *Proposed to have many functions; TGF-beta receptor modulation, RYR1 calcium channel modulation, folding of proteins. | *Multiple functions. Modulation of TGFbeta-1 signaling. (46) | 97,2 |
| P18517 | Non-structural protein 2a | Bovine coronavirus protein |  |  |
| P18892 | Butyrophilin subfamily 1 member A1 | *Can take part in secretion of milk-fat-droplets. Has inhibitory capability against CD4 and CD8 T-cells. | Controls size of lipid droplets in milk^.^ Possible effect on multiple sclerosis (47, 48) | 84,0 |
| P19120 | Heat shock cognate 71 kDa protein | *Multitude of functions in cells. Major influence in the support of re-folding of misfolded proteins. | * Facilitates the proper folding of proteins.  Membrane located protein interacts with VGF-derived bioactive peptide TLQP-21 HSPA8 recognize KFERQ-like motif and triggers unfolding and transport of this proteins to lysosome (49, 50) | 99,4 |
| P19803 | Rho GDP-dissociation inhibitor 1 | Inhibits rho-proteins by inhibition of GDP dissociation. Targets Cdc42. (51-53) | *Control RHO protein homeostasis. Retains rho proteins such as cdc42 rac1 and rhoa in an inactive cytosolic pool.  Meditiates extraction from membranes. Overexpression protects cancer cells from drug-induced apoptosis. (Boulter, Garcia-Mata et al. 2010, Ming, Guo et al. 2014) | 97,1 |
| P19858 | L-lactate dehydrogenase A chain | *Involved in pathway to catalyse (S)-lactate from pyruvate | *As bovine  Is ISGylated as determined by anti-ISG precipitation analysis. (54) | 93,7 |
| P21856 | Rab GDP dissociation inhibitor alph... | Functions in vesicular membrane transport to recycle Rab GTPases. (55-57) Can bind geranylgeranyl in a hydrophobic pocket. | Recycling of Rab GTPases, removal of them from membranes in their inactive forms. | 98,4 |
| P22046 | Matrix protein | Viral protein from rinderpest virus. |  |  |
| P24627 | Lactotransferrin | *Iron binding protein that can bind two Fe^3+^ ions when paired with a suitable anion. Can produce antimicrobial peptides. Serine protease activity  Protease activity similar to trypsin, only with lower rate. Is inhibited by serine protease inhibitors. (58) | *Similar function as with bovine homolog. | 69,7 |
| P25417 | Cystatin-B | *Intracellular thiol protease inhibitor. | Protease inhibitor present in various bodily fluids. (59) | 78,6 |
| P26201 | Platelet glycoprotein 4 | *CD36, multifunctional glycoprotein that can bind multitude of ligands acting as a receptor. | *Similar functions as with bovine homolog.  Possibly participating in free fatty acid uptake in bloodstream and transport of said fatty acids to cardiac tissue. | 83,0 |
| P30086 | Phosphatidylethanolamine-binding pr... | *Binds ATP, opioids and phosphatidylethanolamine. | Acts as a kinase inhibitor for Raf-1-kinase. (60) | 100,0 |
| P30932 | CD9 antigen | *Integral membrane protein associated with integrins. Involved in cell adhesion, cell motility and tumor metastasis. | Suppresses cell motility and metastasis with CHO-cells. (61) | 83,8 |
| P31976 | Ezrin | *Involved in connections of major cytoskeletal structures to the plasma membrane. Required for formation of microvilli and normal macropinosytosis. | Required for requirement of specific GEF to rearrange apical cytoskeleton. (62, 63)  Signal transduction of various signals by connecting receptors from cell membrane to cytoskeleton. Prior of signaling requires activation from PKC-iota. (Wald, Oriolo et al. 2008) | 94,4 |
| P34933 | Heat shock-related 70 kDa protein 2 | *See entry P19120. Molecular chaperone included in variety of processes. | *Similar function as with bovine homolog. | 98,6 |
| P35246 | Pulmonary surfactant-associated pro... | *Defence against inhaled micro-organisms, antigens and toxins. Consists of 90 % lipid and 10 % protein. | *Defence protein against pathogens in lungs.  Decreased expression in adipose tissue with obese and T2D patients. (64) | 72,7 |
| P38409 | Guanine nucleotide-binding protein ... | *See entries P0C7Q4 and P08239 | Transduces the signal from Free fatty acid receptor 4. (65) | 98,6 |
| P48035 | Fatty acid-binding protein, adipocy... | *Lipid transport protein in adipocytes. Transports bound lipids to cognate receptors in nucleus. | Results by similarity to mouse FABP4; activation of transcription of PPAR associated genes. (66) | 84,1 |
| P50397 | Rab GDP dissociation inhibitor beta | *Regulation of GDP/GTP exchange reaction. | *Regulation of GDP/GTP exchange reaction. | 97,1 |
| P55859 | Purine nucleoside phosphorylase | Catalysis of reversible phosphorolysis of deoxyribo nucleosides. (67, 68) | *Catalyze the phosphorolytic cleavage of N-glycosidic bond in the beta-(deoxy)ribonuleoside molecules. | 88,4 |
| P60712 | Actin, cytoplasmic 1 | *Highly conserved protein forming cytoplasmic cytoskeleton. | *Polymerizes to form filaments that form cross-linking cytoskeleton. | 100,0 |
| P61223 | Ras-related protein Rap-1b | *GTPase activity, role in the establishment of basal endothelial barrier function. | Meditates cAMP induced tightening of endothelial junctions. Activation of Rap1 by cAMP induces junctional actin to further tighten cell-cell connections.(69) | 100,0 |
| P61585 | Transforming protein RhoA | *Organization of cytoskeleton. Regulates variety of effector proteins to respond cellular messages. | *Mainly associated with cytoskeleton organization, in active state binds to a variety of effector proteins to regulate cellular responses such cytoskeletal dynamics, cell migration and cell cycle by interaction between receptor proteins and cytoskeleton. (70) | 100,0 |
| P61823 | Ribonuclease pancreatic | *Catalyzes the cleavage of single and double stranded RNA from 3’ end. (71, 72) | Cleavage of single and double stranded RNA from 3’ end. | 74,0 |
| P62157 | Calmodulin | *Binds 4 Ca^2+^ Ions, controls variety of enzymes and ion channels by Ca^2+^ concentrations. | In co-operation with CP110 controls the centrosome formation with centrin. (73) | 100,0 |
| P62261 | 14-3-3 protein epsilon | Part of 14-3-3- adapter protein family.  Adapter protein implicated to be involved in multiple signaling pathways. Can activate protein kinase C in brain. (74) | Involved in heat shock factor regulation. (75) | 100,0 |
| P62739 | Actin, aortic smooth muscle | See entry P60712 |  | 100,0 |
| P62833 | Ras-related protein Rap-1A | *Morphological reversion of cell lines transformed by Ras oncogene. Regulation of KRIT1 localization. Role in NGF-induced growth. | Regulation of Krit1 interactions with microtubules and membranes. (76) | 100,0 |
| P62871 | Guanine nucleotide-binding protein ... | *Signal transduction in various transmembrane signaling systems. | *As with bovine. Defects in expression of said protein leads in variety of neuronal disabilities. | 100,0 |
| P62935 | Peptidyl-prolyl cis-trans isomerase... | *May assist in protein folding by catalyzing the isomerization of prolines. | PPIases accelerate the folding of proteins. It catalyzes the cis-trans isomerization of proline imidic peptide bonds in oligopeptides. (77)  *Targets of anti-rejection drugs in organ transplants | 98,8 |
| P62992 | Ubiquitin-40S ribosomal protein S27... | *Either as free unit or covalently anchored to a protein, of which it can activate or target to variety of events. Component of the 40S subunit of ribosome. | *Similar function as with bovine homolog. | 100,0 |
| P62998 | Ras-related C3 botulinum toxin subs... | *Small GTPase that has variety of functions; phagocytosis of apoptic cells, epithelial cell polarization. Takes part in multiple chemokine triggered events. | Plasma membrane associated small GTPase that has multiple functions; mediation of pinocytosis and related membrane ruffling, in interaction between actin stress fibers, response to growth factors. (78) | 100,0 |
| P63048 | Ubiquitin-60S ribosomal protein L40 | *See entry P62992. Structural part of ribosome 60S subunit |  | 100,0 |
| P63097 | Guanine nucleotide-binding protein ... | *Signal transduction, decreases intracellular cAMP levels by inhibition of adenylate cyclase. | Signal transduction from GPCRs, is modulated by regulator of G protein signaling. (79) | 100,0 |
| P63103 | 14-3-3 protein zeta/delta | Part of 14-3-3- adapter protein family. | Regulation of cytoskeletal rearrangements. (80) | 100,0 |
| P63258 | Actin, cytoplasmic 2 | See entry P60712 |  | 100,0 |
| P68103 | Elongation factor 1-alpha 1 | *Involved in protein biosynthesis, involved in Th1 cytokine production. | Interaction with TXk regulates interferon-gamma gene transcription Th1. (81) | 100,0 |
| P68138 | Actin, alpha skeletal muscle | See entry P60712 |  | 100,0 |
| P68250 | 14-3-3 protein beta/alpha | Part of 14-3-3- adapter protein family. | Involved in bone formation and osteoblast differentiation. (82) | 99,6 |
| P68252 | 14-3-3 protein gamma | Part of 14-3-3- adapter protein family. | Can activate p53 from signals Chk1. (83) | 99,6 |
| P68509 | 14-3-3 protein eta | Part of 14-3-3- adapter protein family. | Negatively regulates 3-phosphoinositide-dependent protein kinase 1. (84) | 99,2 |
| P80025 | Lactoperoxidase | *Enzyme secreted to milk that produces hypothiocyanous acid from thiocyanate and hydrogen peroxide; an antimicrobial acid that has inhibitory properties against various bacteria. | *Similar function as with the bovine homolog. Part of human airway pathogen defense. (85) | 83,0 |
| P80177 | Macrophage migration inhibitory fac... | *Pro-inflammatory cytokine involved in innate immune response to bacterial pathogens. | *Similar function as with bovine homolog. Higher levels in blood of patients with severe sepsis. (86) | 92,2 |
| P80195 | Glycosylation-dependent cell adhesi... | *Binds to identical glycam molecules. | No homolog present in humans. |  |
| P80457 | Xanthine dehydrogenase/oxidase | *Catalyzes the oxidation of hypoxanthine to xanthine, therefore modulating the purine degradation.  Functions as O-type oxidase, or as NAD^+^ dependant, D-type, dehydrogenase. (87) | Participates in purine metabolism. High expression of XDH gene on lung tumors lowers survival of the patients.  (88) | 89,1 |
| P80724 | Brain acid soluble protein 1 | *Function known only as by GO-annotation by molecular similarity. Can bind specific protein domain and function as transcription modulator. | *Similar function as with bovine homolog. Takes part in multiple tissue development and differentiation by GO-annotation. | 72,3 |
| P81125 | Alpha-soluble NSF attachment protei... | Required for vesicular transport between ER and golgi. (89) | Localizes CDH5 to the plasma membrane in co-operation with GNA12. (90) | 97,6 |
| P81265 | Polymeric immunoglobulin receptor | *Binds to polymeric IgA and IgM on epithelial cells. | Binding of IgA and IgM and participating in the transport of the formed complex. Functions as a immunoglobulin receptor. | 66,7 |
| P81287 | Annexin A5 | *Anticoagulant protein, direct inhibitor of thromboplastin-specific complex. | Administration of Annexin A5 prevented the formation of tumor microenvironment by binding of exposed phophatidylserines on apoptic bodies induced by chemotherapy. (91) | 95,9 |
| P82669 | 28S ribosomal protein S25, mitochon... | *Ribosome structural component in mitochondrial ribosomes. | * Similar function as with bovine homolog. | 88,4 |
| P84080 | ADP-ribosylation factor 1 | *Involved in protein trafficking between cellular compartments. Modulates vesicle budding in Golgi. | GTP-binding protein involved in protein trafficking among different compartments. (92) | 100,0 |
| P84081 | ADP-ribosylation factor 2 | *Allostreric activator of cholera toxin catalytic subunit. | *See entry P84080 | 95,6 |
| Q02375 | NADH dehydrogenase [ubiquinone] iro... | *Accessory subunit of the mitochondrial membrane NADH dehydrogenase. (93) | Subunit of the NADH dehydrogenase complex 1. (94) | 90,9 |
| Q04467 | Isocitrate dehydrogenase [NADP], mi... | *Takes part in metabolism and energy production. | *Similar function as with bovine homolog | 96,5 |
| Q05927 | 5'-nucleotidase | *Hydrolysation of extracellular nucleotides to nucleosides. Subunits are held together by non-covalent bonds. (95) | *Similar function as with bovine homolog | 89,9 |
| Q05B83 | Replication factor C subunit 2 | *Elongation of primed DNA templates. | *Similar function as with bovine homolog | 93,5 |
| Q0II59 | Pyridoxal kinase | *Synthesis of pyridoxal-5-phosphate from B6 | Required for synthesis of pyridoxal-5-phosphate from vitamin B6. | 86,9 |
| Q0IID2 | TLD domain-containing protein 2 | *No function defined for this protein |  | 83,4 |
| Q0IIG7 | Ras-related protein Rab-5A | Regulation of endocytosis and passage to early endosomes. Protein HAP40 in huntingtons disease interacts with Rab-5A modulating this process. (96) | Required for homotypic endosome fusion. (97) | 98,6 |
| Q0IIG8 | Ras-related protein Rab-18 | *Participates in apical endocytosis and recycling. Transport between plasma membrane and early endosomes. | Regulates membrane trafficking in organelles and transport vesicles. Rab-18 regulates lipid droplet size. Rab-18 deficient cells produce large, over-sized lipid droplets. (98) | 99,5 |
| Q0P5H5 | Regulator of G-protein signaling 2 | *Regulation of GPCR signaling cascades. Inhibition of signal by increasing the GTPase activity of alpha-subunits. | Negative regulation of Galfa11 mediated inositol-lipid signaling. (99) | 94,3 |
| Q0V7M7-2 | Spindle and kinetochore-associated ... | *Essential for chromosome segregation. | Forms a complex with Ska2 in anaphase to separate chromosomes. (100) | 87,0 |
| Q0VBZ9 | MARCKS-related protein | *Controls cell movement by regulation of actin cytoskeleton. | Phosphorylation of MARCKSL1 is mandatory for the stability of actin filaments in cancer cells and neurons. (101) | 92,5 |
| Q0VC36 | 14-3-3 protein sigma | Part of 14-3-3- adapter protein family.  *When bound to KRT17 regulates protein synthesis and epithelial cell growth. | May activate p53 by degradation of MDM2 by ubiquitination. (102) | 97,6 |
| Q0VC71 | Probable tubulin polyglutamylase TT... | *Catalytic subunit of neuronal tubulin polyglutamylase complex. | *Similar function as with the bovine homolog. | 96,9 |
| Q17QB7 | Ras-related protein Rab-30 | Processes like other Rab-proteins on the list. *Required for structural integrity of Golgi. | Required for the structural integrity of Golgi, transport of vesicles. (103) | 100 |
| Q17QE0 | Uncharacterized protein FAM241A | *Uncharacterized single pass membrane protein. | *As with the bovine homolog. | 81,88 |
| Q17QE5 | Calcium and integrin-binding protei... | *Regulator of variety of cellular processes such as, cell differentiation, division, migration and proliferation, apoptosis, angiogenesis and thrombosis. Involved in interactions with variety of secondary proteins. | Promotes tumor progression by regulating angiogenesis. (104) | 94,2 |
| Q17QU4 | Ras-related protein Rab-39B | Process similar to other Rab-proteins in list. *Involved in autophagy and intracellular membrane trafficking. | Involved in autophagy and vesicle tethering and fusion processes. (105) | 99,5 |
| Q1LZC5 | Ankyrin repeat domain-containing pr... | *Regulation of intracellular signaling associated with erythroid differentiation. | *Similar function as with bovine homolog. | 93,1 |
| Q1LZC9 | DPH3 homolog | *Synthesizes the first step of post-translational modification of histidine to diphthamide, which occurs in elongation factor 2. | Is a target for bacterial ADP-ribosylating toxins. (106) | 96,3 |
| Q1RMJ6 | Rho-related GTP-binding protein Rho... | *Regulates signal transduction pathway linking plasma membrane receptors to actin cell stress fibers and focal adhesions. | Regulator of cytokinesis, mediates the formation and location of the cytokinesis inducing contractile ring via microtubule-dependent signal. (107) | 100 |
| Q1RMR4 | Ras-related protein Rab-15 | *May act with RAB3A in regulating aspects of synaptic vesicle membrane flow. | *Similar function as with bovine homolog. | 97,6 |
| Q27960 | Sodium-dependent phosphate transpor... | *Can be involved in active transport of phosphate by co-transporting Na^+^ ions. | May be involved in actively transporting phosphate in cells. Tumor-associated antigen. (108) | 76,7 |
| Q27965 | Heat shock 70 kDa protein 1B | See entries for other heat shock proteins. |  | 98,9 |
| Q27975 | Heat shock 70 kDa protein 1A | See entries for other heat shock proteins. |  | 98,9 |
| Q28024 | Guanine nucleotide-binding protein ... | See entries for other guanine binding proteins. |  | 94,4 |
| Q28115 | Glial fibrillary acidic protein | *Class-III intermediate filament; a cell specific marker that separates astrocytes from glial cells during development of CNS. | *Similar function as with bovine homolog | 95,4 |
| Q28181 | Cyclic nucleotide-gated cation chan... | *Subunit of a cyclic nucleotide gated channel. These channels play important roles in visual and olfactory signal transduction. | *Similar function as with bovine homolog. | 84,7 |
| Q28203 | Tumor necrosis factor receptor supe... | *CD40; receptor for TNFSF5, signals activate ERK in macrophages and B cells | Capable of binding integrin a5b1 and modulating CD40 signaling. (1) | 67,1 |
| Q28824 | Myosin light chain kinase, smooth m... | *Takes part in smooth muscle contraction.(109, 110) | *Multiple functions. Participates in cell migration and tumor metastasis among others. (Zhou, Liu et al. 2008) | 92,7 |
| Q29443 | Serotransferrin | *Part of transferrin family. Transport of iron ions with association of an anion. | *Similar function as with bovine homolog. | 68,6 |
| Q29RR0 | Ras-related protein Rab-26 | *Participates in exocrine secretion. | Similar to other Rab proteins. Takes part in exocrine secretion. (111) | 87,5 |
| Q2HJ38 | Calponin-1 | *Associated with thin filaments, implicated to regulation of smooth muscle contraction | *Capable of binding actin, calmodulin, troponin C and tropomysin. | 98,7 |
| Q2HJ49 | Moesin | *Part of Ezrin-radixin-moesin family that connects the actin cytoskeleton to the plasma membrane. | T558 is critical for control of actin binding; possible defects could diminish or activate Moesin. (112) | 98,6 |
| Q2HJB9 | Transmembrane protein 98 | *Negative regulator of MYRF in oligodendrocyte differentiation. Secreted form promotes differentiation of Th1 cells. | Secreted TM98 promotes differentiation of Th1 cells. (113) | 98,2 |
| Q2HJH2 | Ras-related protein Rab-1B | *Takes part in initial events of autophagic vacuole development. | Able to recruit to membranes different set of downstream effectors; vesicle formation, movement, tethering and fusion. (114) | 99,5 |
| Q2HJI8 | Ras-related protein Rab-8B | *May play a role in polarized vesicular trafficking and neurotransmitter release. | *May participate in cell junction dynamics in Sertoli cells. | 98,1 |
| Q2KHV5 | Protein arginine methyltransferase ... | *Involved in the assembly or stability of mitochondial NADH:ubiquinone oxidoreductase complex. | Methylates arginine 85 in NDUFS2 that is required in formation or stability of complex 1. (115) | 83,7 |
| Q2KIC6 | Neuritin | *Promotes neurite outgrowth and especially branching of neuritic processes in primary hippocampal and cortical cells | *Similar function as with bovine homolog. | 97,9 |
| Q2KID9 | 28S ribosomal protein S5, mitochond... | *Structural component of ribosome, binds RNA | *Similar function as with bovine homolog | 82,1 |
| Q2KIE2 | HCLS1-associated protein X-1 | *Involved in clathrin-mediated endocytosis pathway. May inhibit Casp9 and Casp3. | Protects myocytes from apoptosis if overexpressed. Regulates carcinoma cell migration by clathrin mediated endocytosis of integrin avbeta6. | 85,7 |
| Q2KIJ1 | Shieldin complex subunit 1 | *Part of shieldin complex which takes part in repair of DNA double-stranded breaks. | Regulator of non-homologous end joinment in DNA double stranded breaks. (116) | 74,6 |
| Q2KJ32 | Methanethiol oxidase | *Oxidation of methanethiol; an organosulfur compound produced by gut bacteria. Binds selenium and can be involved in sensing of xenobiotics.  Can be involved in protein transport in golgi. (117) | *Similar function as with bovine counterpart.  Mutations cause extraoral halitosis.(118) | 91,1 |
| Q2KJ93 | Cell division control protein 42 ho... | *Multiple functions in cells, has GTPase activity and is controlled by GEFs. | Works in metaphase by regulation of bi-orient attachment of spindle microtubules to kineotochores. Regulation of cell migration. (119, 120) | 100 |
| Q2KJD3 | Spliceosome-associated protein CWC1... | *Pre-mRNA processing as part of spliceosome. | *Similar function as with bovine homolog. (121) | 99,1 |
| Q2NKU0 | Centromere protein X | *DNA-binding in the Fanconi anemia complex. | Upholds genomic stability, participates in Fanconi Anemia pathway. (122, 123) | 80,3 |
| Q2TA29 | Ras-related protein Rab-11A | *Regulates endocytic recycling and one factor involved in exocytosis. | Required for completion of cytokinesis. Involved in transporting endosomes to the cleavage furrow | 99,5 |
| Q2TBH7 | Ras-related protein Rab-4A | *Protein transport, may partake in vesicular traffic. | In association of vascular endothelial growth factor receptor-2 signaling regulates endosomal trafficking. | 99,1 |
| Q2TBN1 | SWI/SNF-related matrix-associated a... | *Involved in transcriptional regulation by chromatin remodeling. May be a link between chromatin remodeling complex and D-vitamin receptor. | Chromatin remodeling by formation of SWI/SNF complexes. (124, 125) | 98,8 |
| Q2TBW7 | Sorting nexin-2 | *Involved in multiple intracellular trafficking pathways. | Involved in retromer-mediated transport; endosome-to trans golgi network.(126) | 97,3 |
| Q2YDG0 | G-protein coupled receptor family C... | *Retinoic acid-inducible G-protein coupled receptor. | *Similar function as bovine homolog. | 86,7 |
| Q32LE3 | Centrin-1 | *Fundamental role in microtubule-organizing center structure. | *Similar function as bovine homolog. (127) | 89,5 |
| Q32LI3 | Uncharacterized protein C12orf71 ho... | *Uncharacterized protein that is found in across eukaryota. |  | 51,0 |
| Q32LL9 | Centromere protein N | *Assembly of kinetochore proteins. | Part of centromere protein complex.(128) | 85,6 |
| Q32LP2 | Radixin | *Probably plays a part in binding of barbed ends of actin filaments to plasma membrane. | *Similar function as with bovine homolog. | 99,14 |
| Q32PA8 | Mth938 domain-containing protein | *May participate in preadipocyte differentiation and adipogenesis. | *Similar function as with bovine homolog.  Induces adipogenesis in 3T3-L1 mouse cell model. (129) | 89,3 |
| Q32PH8 | Elongation factor 1-alpha 2 | *Promotes GTP-dependent binding of aminoacyl-tRNA to A-site of ribosomes. | *Similar function as with bovine homolog. | 100 |
| Q3C2I0 | Bcl-2-related protein A1 | *Retards apoptosis induced by IL-3 deprivation. | *Similar function as with bovine homolog.  In mice cell model prevents serum starvation induced apoptosis by directly interacting with mINGh and Bcl-2 pathway. (130) | 78,2 |
| Q3MHP2 | Ras-related protein Rab-11B | *See other Rab-proteins in the list. Participates in various transmembrane transport processes. | Participates in exocytosis. (131) | 100 |
| Q3MHX6 | Protein OS-9 | *Lectin that functions in ER by regulating the secretion and degradation of misfolded glycoproteins. | Lectin that functions in ER by preventing the secretion of misfolded glycoproteins and secondly as a promoter of protein disposal from ER lumen. Is overexpressed in tumors. Regulates the ubiquitination of TRPV4. (132, 133) | 90,1 |
| Q3SZ62 | Phosphoglycerate mutase 1 | *Interconversion of 3-and 2-phosphoglycerate. | *Similar function as bovine homolog. | 99,2 |
| Q3SZ86 | 28S ribosomal protein S26, mitochon... | *Structural part of mitochondrial ribosomes. | *As with bovine homolog. | 78,1 |
| Q3SZ97 | Small integral membrane protein 7 | *Integral membrane protein with no known function. | *Similar as bovine homolog. | 98,7 |
| Q3SZF2 | ADP-ribosylation factor 4 | *GTP binding protein, that functions as an allosteric activator of the cholera toxin catalytic subunit. May modulate vesicle budding and uncoating within Golgi. | *Similar function as bovine homolog. Mediates epidermal growth factor receptor -dependent phospholipase D2 activation. (134) | 96,7 |
| Q3SZI4 | 14-3-3 protein theta | *Part of 14-3-3- adapter protein family. | Regulation of 3-phosphoinositide-dependent protein kinase-1. (84) | 100 |
| Q3SZK8 | Na(+)/H(+) exchange regulatory cofa... | *Scaffold protein that links plasma membrane proteins to cytoskeleton complex of erzin/moesin/radixin family. Part of beta-2 adrenergic receptor binding pathway. | Functions in interaction with neurofibromatosis 2 tumor suppressor merlin. Is inhibited by cAMP -dependent protein kinases E3KARP and NHERF. Participates in beta adrenergic receptor signaling. (135-137) | 85,6 |
| Q3SZP2 | Microtubule-associated protein RP/E... | *May be involved in microtubule polymerization | *Similar function as bovine homolog. | 97,6 |
| Q3T000 | Synaptobrevin homolog YKT6 | *v-SNARE mediating vesicle docking and fusion to a specific cellular compartment. Part of ER-Golgi transport. Part of early/recycling endosome transport. | Prenylated v-SNARE required in ER-Golgi transport. Participation in early/recycling endosome transport TGN. (138, 139) | 92,4 |
| Q3T0D7 | GTP-binding protein SAR1a | *Involved in transport from ER to Golgi. | Interacts with Sec16 which is required for secretion of cargo in ER. | 99,0 |
| Q3T0F5 | Ras-related protein Rab-7a | *Key regulator in endo-lysosomal trafficking. Required for the exosomal release of SDCBP, CD63 and syndecan. | Required for exosomal release. Key regulator in endo-lysosomal transport. (140, 141) | 98,1 |
| Q3T0N1 | Multivesicular body subunit 12A | *Component of ESCRT-I complex. | Interacts with CFBP and can be part of regulation of EGF-receptor. (142) | 88,3 |
| Q3T0Q4 | Nucleoside diphosphate kinase B | *Synthesis of nucleosides other than ATP | *Similar function as bovine homolog. May act as oncogene suppressor. (143) | 93,4 |
| Q3T145 | Malate dehydrogenase, cytoplasmic | *Dehydrogenates S-malate to oxaloacetate. | *Similar function as with bovine homolog. | 95,5 |
| Q3ZBE5 | Pre-mRNA-splicing factor SLU7 | *Required part of spliceasome, a pre-mRNA splicing complex. | Required part of spliceasome, If removed splicing will not occur. (144) | 97,6 |
| Q3ZBR9 | Ermin | *Rearrangement of cytoskeleton in late wrapping phases of myelinogenesis. | *Similar function as with bovine homolog. | 73,1 |
| Q3ZBW5 | Rho-related GTP-binding protein Rho... | *Mediates apoptosis in neoplastically transformed cells after DNA damage. | Targets PRK1 to endosomes. Regulates DNA induced cell death. (145, 146) | 100 |
| Q3ZBZ1 | 45 kDa calcium-binding protein | *May regulate calcium-dependent activities in the ER lumen or post-ER compartment. | *Similar function as bovine homolog. | 84,5 |
| Q3ZCD0 | CD81 antigen | *Structural component of tetraspanin enriched microdomains. | Binds cholesterol in a hydrophobic pocket. Effects CD19 on B-lymphocytes. Deficiencies in CD81 expression, will lead in antibody deficiency. (147-149) | 94,5 |
| Q49BZ4 | C-type lectin domain family 7 membe... | *Recognition of beta-1,3-linked and beta-1,6 linked glucans. | Single pass transmembrane protein that binds specific beta-glucans and T-lymphocyte specific binding sites. (150) | 73,7 |
| Q4GZT4 | Broad substrate specificity ATP-bin... | *Transport of variety of toxins, xenobiotics from cells. | *Transport of variety of substances from cells. Overexpression or mutations can cause to chemotherapy resistance in cancer cells. | 84,5 |
| Q58DM4 | DNA oxidative demethylase ALKBH2 | *Repaires damaged DNA and RNA containing 1-methyladenine and 3-methylcytosine. | DNA repair by removing methylation from 1-methyladenine and 3-methylcytosine. | 84,3 |
| Q58DS5 | Ras-related protein Rab-13 | *See other Rab-proteins on the list. Regulators of intracellular membrane trafficking. Participates in endosomal recycling. | Endosomal recycling, formation of tight junctions. (78, 151) | 97,5 |
| Q58DS9 | Ras-related protein Rab-5C | *See other Rab-proteins on the list. | *Similar function as with bovine homolog. | 99,1 |
| Q5E946 | Protein/nucleic acid deglycase DJ-1 | *Multifunctional protein with deglycase activity by similarity | *Multifunctional protein with function still under further analysis | 96,3 |
| Q5E947 | Peroxiredoxin-1 | *Thiol specific peroxidase that reduces hydrogen peroxidase and organic hydroperoxidases to water and alcohols. | Signal transduction by regulating the HOOH-levels induced by growth factors and TNF-alfa. Homolog protein controls neuronal differentiation in chickens. (152, 153) | 97,0 |
| Q5E9B1 | L-lactate dehydrogenase B chain | *Catalysis of lactate oxidation to pyruvate. | *Similar function as with bovine homolog. | 98,2 |
| Q5E9B5 | Actin, gamma-enteric smooth muscle | *Component of cytoskeleton. | *Similar function as with bovine homolog. | 100 |
| Q5E9D1 | Small ubiquitin-related modifier 1 | *Ubiquitin-like protein that can function in a similar fashion as ubiquitin. SUMOylation has different effects depending on the location and the target of the modification. | *Similar function as with bovine homolog. Regulates KV2.1 voltage channel and pancreatic beta-cell excitability. (154) | 100 |
| Q5E9E7 | Glycosyltransferase 8 domain-contai... | *Transfer glycosyl groups between molecules. | *Similar function as with bovine homolog. | 92,2 |
| Q5E9F7 | Cofilin-1 | *Binds to F-actin. Regulates cytoskeleton dynamics. | Regulation of actin depolymerization along with ADF. (155) | 99,4 |
| Q5E9H8 | Probable G-protein coupled receptor... | *Receptor for SMIM20 derived peptides; Phoenixin-14 and Phoenixin-20. Mediates the release of GNRH in the hypothalamus and pituitary gland. | Proliferation of cells, acting through cAMP/PKA pathway. (156) | 99,5 |
| Q5E9I6 | ADP-ribosylation factor 3 | *GTP-binding protein that functions as an allosteric activator of the cholera toxin. | *Similar function as with bovine homolog. | 100 |
| Q5E9J7 | tRNA-specific adenosine deaminase 2 | *May participate in deamination of adenosine-34 to inosine. | *Similar function as with bovine homolog. | 89,5 |
| Q5E9Z7 | DNA-directed RNA polymerase III sub... | *Functions as intracellular DNA sensor for pathogenic response in innate immune response. Part of PolIII that produces | Part of RNAPOLIII, that functions as DNA-dependent RNA polymeras, but also an intracellular DNA sensor for pathogens. Induces Interferon I response through RIG-I pathway. (157) | 92,7 |
| Q5EA50 | Rab9 effector protein with kelch mo... | *Required for endosome to trans-Golgi network transport. | Required for endosome to trans-Golgi network transport. (158) | 85,0 |
| Q5EA79 | Aldose 1-epimerase | *Mutarotase that converts alpha-aldose to beta-anomer. | *Similar function as with bovine homolog. (159) | 90,1 |
| Q5EA88 | Glycerol-3-phosphate dehydrogenase ... | *Carbohydrate metabolism | *Similar function as with bovine homolog. | 93,1 |
| Q6VE48 | Membrane cofactor protein | *Cofactor for complement factor I. Cleavage of complement C3b and C4b. | CD46, costimulatory protein for T-cells that promotes proliferation. (160) | 46,6 |
| Q8MI01 | Mucin-15 | *Glycoprotein mainly expressed on apical surfaces of mammary epithelial cells. | *May play a role in cell adhesion to the extracellular matrix. | 68,0 |
| Q8WML4 | Mucin-1 | *Glycoprotein that may have protective properties on the extracellular side of epithelial cells. Modulates various intracellular signaling pathways. | *Glycoprotein with adhesion and anti-adhesion properties on extracellular surface of cells. Involved in various signaling pathways from it’s C-terminal domain. | 53,8 |
| Q95114 | Lactadherin | Promotes phagocytosis of apoptotic cells. High expression of lactadherin enhances bladder tumor development possible by depressing anti-tumor immune responses. (161-163) | Various cellular interactions such as removal of apoptotic cells in various tissues via phagocytosis. | 64,9 |
| Q95122 | Monocyte differentiation antigen CD... | *Coreceptor for bacterial LPS. | Receptor for LPS and LPS binding protein. (164) | 74,0 |
| Q9GLM3 | X-linked retinitis pigmentosa GTPas... | *Required for normal location of RPGR in photoreceptor cells and for survival of photoreceptor cells. | Similar function as with bovine homolog. (165) | 73,1 |
| Q9MZ06 | Fibroblast growth factor-binding pr... | *Acts as a carrier protein for fibroblast-binding factors, release enhances mitogenic activity. | *Similar function as with bovine homolog. (166) | 57,9 |
| Q9N0K1 | Leukocyte surface antigen CD47 | *Cell adhesion and modulation of integrins. | Interaction with integrins. (167) | 71,2 |
| Q9N1Q8 | Calcium-binding protein 5 | *May positively regulate neurotransmitter vesicle endocytosis and exocytosis in a salt-dependent matter. | *Inhibition of calcium-dependent inactivation of L-type calcium channel. | 94,8 |
| Q9TU25 | Ras-related C3 botulinum toxin subs... | *Plasma membrane-associated small GTPase which cycles between an active GTP-bound and inactive GDP bound state. | Regulates the formation of superoxide anion O^2-^ by a multicomponent oxidase. (168) | 98,4 |
| Q9TUM6 | Perilipin-2 | *May be involved in development and maintenance of adipose tissue. | Formation of lipid droplets. (169) | 88,1 |
| Q9XSA7 | Chloride intracellular channel prot... | *Can insert into membranes and form chloride ion channels. | *Can insert into membranes. Has multiple regulatory effects in cells, to induce differentiation and proliferation. (170) | 98,8 |
| Q9XSG3 | Isocitrate dehydrogenase [NADP] cyt... | Could function in corneal epithelium and uphold the tissue transparency. (171) | *Oxidation of isocitrate to 2-oxoglutarate. | 95,4 |
| Q9XSJ4 | Alpha-enolase | *Glycolytic enzyme that converses 2-phosphoglycerate to phosphoenolpyruvate.  Functions as a hypoxia induced stress protein. | *Glycolytic enzyme that has similar function as bovine homolog. Acts as a transcriptional repressor and could function as tumor repressor. | 95,6 |
| Q9XSK2 | CD63 antigen | *Multiple functions in cells such as activation of ITGB1, promotion of cell survival, adhesion and migration. Is commonly used as an exosome marker protein. | Multiple functions; interaction with metalloproteinase-1. Recruitment of leukocytes by interactions with P-selectins. Endosomal sorting. (172-174) | 84,9 |
| Q9YWQ0 | Non-structural protein 3 | Viral protein |  |  |
| P04896 | Guanine nucleotide-binding protein ... | *Interaction and downstream signalling with multiple different GPCRs. | *Similar function as bovine homolog. | 99,8 |

- *From Uniprot entry

References

References

1. Hu F, Zhu Q, Sun B, Cui C, Li C, Zhang L. Smad ubiquitylation regulatory factor 1 promotes LIM‐homeobox gene 9 degradation and represses testosterone production in Leydig cells. The FASEB Journal. 2018 Sep;32(9):4627-40.

2. Popow J, Jurkin J, Schleiffer A, Martinez J. Analysis of orthologous groups reveals archease and DDX1 as tRNA splicing factors. Nature. 2014 Jul 3,;511(7507):104-7.

3. Bryant DM, Datta A, Rodríguez-Fraticelli AE, Peränen J, Martín-Belmonte F, Mostov KE. A molecular network for de novo generation of the apical surface and lumen. Nature cell biology. 2010 Nov;12(11):1035-45.

4. Sellier C, Campanari M, Julie Corbier C, Gaucherot A, Kolb‐Cheynel I, Oulad‐Abdelghani M, et al. Loss of C9ORF72 impairs autophagy and synergizes with polyQ Ataxin‐2 to induce motor neuron dysfunction and cell death. The EMBO Journal. 2016 Jun 15,;35(12):1276-97.

5. Roland JT, Bryant DM, Datta A, Itzen A, Mostov KE, Goldenring JR. Rab GTPase-Myo5B complexes control membrane recycling and epithelial polarization. . 2011 Feb 15,.

6. Park I, Han C, Jin S, Lee B, Choi H, Kwon JT, et al. Myosin regulatory light chains are required to maintain the stability of myosin II and cellular integrity. Biochem J. 2011 /02/15;434(1):171-80.

7. Lou J, Chen H, Han J, He H, Huen MSY, Feng X, et al. AUNIP/C1orf135 directs DNA double-strand breaks towards the homologous recombination repair pathway. Nature communications. 2017 Oct 17,;8(1):985-14.

8. Ai J, Ai J, Wang Y, Wang Y, Tan K, Tan K, et al. A human homolog of mouse Lbh gene, hLBH, expresses in heart and activates SRE and AP-1 mediated MAPK signaling pathway. Mol Biol Rep. 2008 Jun;35(2):179-87.

9. Rubin A, Salzberg AC, Imamura Y, Grivitishvilli A, Tombran-Tink J. Identification of novel targets of diabetic nephropathy and PEDF peptide treatment using RNA-seq. BMC genomics. 2016 Nov 17,;17(1):936.

10. Bakulski KM, Lee H, Feinberg JI, Wells EM, Brown S, Herbstman JB, et al. Prenatal mercury concentration is associated with changes in DNA methylation atTCEANC2in newborns. International Journal of Epidemiology. 2015 Aug;44(4):1249-62.

11. Kanie T, Abbott KL, Mooney NA, Plowey ED, Demeter J, Jackson PK. The CEP19-RABL2 GTPase Complex Binds IFT-B to Initiate Intraflagellar Transport at the Ciliary Base. Developmental Cell. 2017 Jul 10,;42(1):22,36.e12.

12. Li C, Bademci G, Subasioglu A, Diaz-Horta O, Zhu Y, Liu J, et al. Dysfunction of GRAP , encoding the GRB2-related adaptor protein, is linked to sensorineural hearing loss. Proceedings of the National Academy of Sciences of the United States of America. 2019 Jan 22,;116(4):1347-52.

13. Braun DA, Schueler M, Halbritter J, Gee HY, Porath JD, Lawson JA, et al. Whole exome sequencing identifies causative mutations in the majority of consanguineous or familial cases with childhood-onset increased renal echogenicity. Kidney International. 2016 Feb;89(2):468-75.

14. Wanschers BFJ, van de Vorstenbosch R, Schlager MA, Splinter D, Akhmanova A, Hoogenraad CC, et al. A role for the Rab6B Bicaudal–D1 interaction in retrograde transport in neuronal cells. Experimental Cell Research. 2007;313(16):3408-20.

15. Adas F, Salaün JP, Berthou F, Picart D, Simon B, Amet Y. Requirement for omega and (omega;-1)-hydroxylations of fatty acids by human cytochromes P450 2E1 and 4A11. Journal of lipid research. 1999 Nov;40(11):1990.

16. Aron B. Fisher, Chandra Dodia, Yefim Manevich, Jin-Wen Chen, Sheldon I. Feinstein. Phospholipid Hydroperoxides Are Substrates for Non-selenium Glutathione Peroxidase. Journal of Biological Chemistry. 1999 Jul 23,;274(30):21326-34.

17. Sang Won Kang, Ivan C. Baines, Sue Goo Rhee. Characterization of a Mammalian Peroxiredoxin That Contains One Conserved Cysteine. Journal of Biological Chemistry. 1998 Mar 13,;273(11):6303-11.

18. Kinya Nishimura, Tadahiko Hazato. Isolation and identification of an endogenous inhubitor of enkephalin-degrading enzymes from bovine spinal cord. . 1993 June 17,.

19. Bhattacharya S, Large E, Heizmann CW, Hemmings BA, Chazin WJ. Structure of the Ca2+/S100B/NDR Kinase Peptide Complex:  Insights into S100 Target Specificity and Activation of the Kinase. Biochemistry. 2003 -12-01;42(49):14416-26.

20. Benoît Gilquin, Brian R. Cannon, Arnaud Hubstenberger, Boualem Moulouel, Elin Falk, Nicolas Merle, et al. The Calcium-Dependent Interaction between S100B and the Mitochondrial AAA ATPase ATAD3A and the Role of This Complex in the Cytoplasmic Processing of ATAD3A. Molecular and Cellular Biology. 2010 Jun 1,;30(11):2724-36.

21. Majava V, Polverini E, Mazzini A, Nanekar R, Knoll W, Peters J, et al. Structural and Functional Characterization of Human Peripheral Nervous System Myelin Protein P2. PloS one. 2010 Apr 22,;5(4):e10300.

22. Chen R, Runswick MJ, Carroll J, Fearnley IM, Walker JE. Association of two proteolipids of unknown function with ATP synthase from bovine heart mitochondria. FEBS Letters. 2007;581(17):3145-8.

23. Majorek KA, Porebski PJ, Dayal A, Zimmerman MD, Jablonska K, Stewart AJ, et al. Structural and immunologic characterization of bovine, horse, and rabbit serum albumins. Molecular Immunology. 2012 Oct;52(3-4):174-82.

24. Lu J, Stewart AJ, Sadler PJ, Pinheiro TJT, Blindauer CA. Albumin as a zinc carrier: properties of its high-affinity zinc-binding site. Biochemical Society transactions. 2008 Dec;36(Pt 6):1317-21.

25. Gaétan Mayer, Steve Poirier, Nabil G. Seidah. Annexin A2 Is a C-terminal PCSK9-binding Protein That Regulates Endogenous Low Density Lipoprotein Receptor Levels. Journal of Biological Chemistry. 2008 Nov 14,;283(46):31791-801.

26. Ramachandran S, Cerione RA. A Dominant-negative Gα Mutant That Traps a Stable Rhodopsin-Gα-GTP-βγ Complex. Journal of Biological Chemistry. 2011 Apr 8,;286(14):12702-11.

27. Jastrzebska B, Orban T, Golczak M, Engel A, Palczewski K. Asymmetry of the rhodopsin dimer in complex with transducin. The FASEB Journal. 2013 Apr;27(4):1572-84.

28. Naeem MA, Chavali VRM, Ali S, Iqbal M, Riazuddin S, Khan SN, et al. GNAT1 associated with autosomal recessive congenital stationary night blindness. Investigative ophthalmology & visual science. 2012 Mar;53(3):1353-61.

29. S E Navon, B K Fung. Characterization of transducin from bovine retinal rod outer segments. Mechanism and effects of cholera toxin-catalyzed ADP-ribosylation. Journal of Biological Chemistry. 1984 May 25,;259(10):6686.

30. van Aalten DMF, Milne KG, Zou JY, Kleywegt GJ, Bergfors T, Ferguson MAJ, et al. Binding site differences revealed by crystal structures of Plasmodium falciparum and bovine acyl-CoA binding protein. Journal of Molecular Biology. 2001;309(1):181-92.

31. Patrick W. Gray, Debra Glaister, Peter H. Seeburg, Alessandro Guidotti, Erminio Costa. Cloning and Expression of cDNA for Human Diazepam Binding Inhibitor, a Natural Ligand of an Allosteric Regulatory Site of the γ -aminobutyric Acid Type A Receptor. Proceedings of the National Academy of Sciences of the United States of America. 1986 Oct 1,;83(19):7547-51.

32. K Shirabe, T Yubisui, N Borgese, C Y Tang, D E Hultquist, M Takeshita. Enzymatic instability of NADH-cytochrome b5 reductase as a cause of hereditary methemoglobinemia type I (red cell type). Journal of Biological Chemistry. 1992 Oct 5,;267(28):20416.

33. Takesue S, Omura T. Purification and Properties of NADH-cytochrome b5 Reductase Solubilized by Lysosomes from Rat Liver Microsomes. Journal of biochemistry. 1970 Feb;67(2):267-76.

34. Udo Seedorf, Sigrid Scheek, Thomas Engel, Christian Steif, Hans-Jurgen Hinz, Gerd Assmann. Structure-Activity Studiesof Human Sterol Carrier Protein 2". The Journal of Biological Chemistry. 1993 August, 25,;269.

35. Tizzano M, Merigo F, Sbarbati A. Evidence of solitary chemosensory cells in a large mammal: the diffuse chemosensory system in Bos taurus airways. Journal of Anatomy. 2006 Sep;209(3):333-7.

36. Fehr J, Fehr J, Meyer D, Meyer D, Widmayer P, Widmayer P, et al. Expression of the G-protein α-subunit gustducin in mammalian spermatozoa. J Comp Physiol A. 2007 Jan;193(1):21-34.

37. Hyeung-Jin Jang, Zaza Kokrashvili, Michael J. Theodorakis, Olga D. Carlson, Byung-Joon Kim, Jie Zhou, et al. Gut-Expressed Gustducin and Taste Receptors Regulate Secretion of Glucagon-Like Peptide-1. Proceedings of the National Academy of Sciences of the United States of America. 2007 Sep 18,;104(38):15069-74.

38. Kikuchi K, Fukuda M, Ito T, Inoue M, Yokoi T, Chiku S, et al. Transcripts of unknown function in multiple-signaling pathways involved in human stem cell differentiation. Nucleic Acids Research. 2009 Aug;37(15):4987-5000.

39. Jürgen Radons. The human HSP70 family of chaperones: where do we stand? Cell Stress and Chaperones. 2016 May 1,;21(3):379-404.

40. Komander D. The emerging complexity of protein ubiquitination. Biochemical Society transactions. 2009 Oct;37(Pt 5):937-53.

41. Yanmin Yang, Eva Spitzer, Nicholas Kenney, Wolfgang Zschiesche, Minglin Li, Arno Kromminga, et al. Members of the Fatty Acid Binding Protein Family Are Differentiation Factors for the Mammary Gland. The Journal of Cell Biology. 1994 Nov 1,;127(4):1097-109.

42. Anne Mette Buhl, Shoji Osawa, Gary L. Johnson. Mitogen-activated Protein Kinase Activation Rquires Two Signal Inputs  from the Human Anaphylatoxin C5a Receptor. The Journal of Biological Chemistry. 1995.

43. Banfield MJ, Barker JJ, Perry AC, Brady RL. Function from structure? The crystal structure of human phosphatidylethanolamine-binding protein suggests a role in membrane signal transduction. Structure. 1998;6(10):1245-54.

44. Gorelik A, Randriamihaja A, Illes K, Nagar B. Structural basis for nucleotide recognition by the ectoenzyme CD203c. The FEBS Journal. 2018 Jul;285(13):2481-94.

45. Nofer J, Remaley AT, Feuerborn R, Wolinnska I, Engel T, von Eckardstein A, et al. Apolipoprotein A-I activates Cdc42 signaling through the ABCA1 transporter. Journal of Lipid Research. 2006 Apr 1,;47(4):794-803.

46. Chen Y, Liu F, Massagué J. Mechanism of TGFβ receptor inhibition by FKBP12. The EMBO Journal. 1997 July 1,;16(13):3866-76.

47. Stefferl A, Schubart A, Storch2 M, Amini A, Mather I, Lassmann H, et al. Butyrophilin, a Milk Protein, Modulates the Encephalitogenic T Cell Response to Myelin Oligodendrocyte Glycoprotein in Experimental Autoimmune Encephalomyelitis. The Journal of Immunology. 2000 Sep 1,;165(5):2859-65.

48. Sherry L. Ogg, Anne K. Weldon, Lorraine Dobbie, Andrew J. H. Smith, Ian H. Mather, Ransom L. Baldwin. Expression of Butyrophilin (Btn1a1) in Lactating Mammary Gland Is Essential for the Regulated Secretion of Milk-Lipid Droplets. Proceedings of the National Academy of Sciences of the United States of America. 2004 Jul 6,;101(27):10084-9.

49. Akhter S, Chakraborty S, Moutinho D, Álvarez-Coiradas E, Rosa I, Viñuela J, et al. The human VGF-derived bioactive peptide TLQP-21 binds heat shock 71 kDa protein 8 (HSPA8)on the surface of SH-SY5Y cells. PloS one. 2017;12(9):e0185176.

50. Robert G, Jacquel A, Auberger P. Chaperone-Mediated Autophagy and Its Emerging Role in Hematological Malignancies. Cells. 2019 Oct 16,;8(10):1260.

51. Nomanbhoy TK, Rosen MK, Gosser YQ, Aghazadeh B, Cerione RA, Manor D, et al. C-terminal binding domain of Rho GDP-dissociation inhibitor directs N-terminal inhibitory peptide to GTPases. Nature. 1997 Jun 19,;387(6635):814-9.

52. Boulter E, Garcia-Mata R, Guilluy C, Dubash A, Rossi G, Brennwald PJ, et al. Regulation of RhoGTPase crosstalk, degradation and activity by RhoGDI1. Nature cell biology. 2010 Apr 1,;12(5):477-83.

53. Ming Z, Guo C, Jiang M, Li W, Zhang Y, Fan N, et al. Bioinformatics analysis of Rab GDP dissociation inhibitor beta and its expression in non-small cell lung cancer. Diagnostic pathology. 2014 Nov 4,;9(1):201.

54. Carolina Villarroya-beltri, Francesc Baixauli, María Mittelbrunn, Irene Fernández-delgado, Daniel Torralba, Olga Moreno-gonzalo, et al. ISGylation controls exosome secretion by promoting lysosomal degradation of MVB proteins. Nature Communications. 2016 Nov 1,;7(1):13588.

55. Luan P, Heine A, Zeng K, Moyer B, Greasely SE, Kuhn P, et al. A New Functional Domain of Guanine Nucleotide Dissociation Inhibitor (α‐GDI) Involved in Rab Recycling. Traffic. 2000 Mar;1(3):270-81.

56. Kirsten ML, Baron RA, Seabra MC, Ces O. Rab1a and Rab5a preferentially bind to binary lipid compositions with higher stored curvature elastic energy. Molecular Membrane Biology. 2013 Jun;30(4):303-14.

57. An Y, Shao Y, Alory C, Matteson J, Sakisaka T, Chen W, et al. Geranylgeranyl Switching Regulates. Structure. 2003;11(3):347-57.

58. Massucci M, Giansanti F, Di Nino G, Turacchio M, Giardi M, Botti D, et al. Proteolytic activity of bovine lactoferrin. Biometals. 2004 Jun;17(3):249-55.

59. Magnus Abrahamson, Alan J. Barrett, Guy Salvesen, Anders Grubb. Isolation of Six Cysteine ProteinaseInhibitors from Human Urine. The Journal of Biological Chemistry. 1986 March.

60. Rath O, Park S, Tang H, Banfield MJ, Brady RL, Lee YC, et al. The RKIP (Raf-1 Kinase Inhibitor Protein) conserved pocket binds to the phosphorylated N-region of Raf-1 and inhibits the Raf-1-mediated activated phosphorylation of MEK. Cellular Signalling. 2008 May;20(5):935-41.

61. Ikeyama S, Koyama M, Yamaoko M, Sasada R, Miyake M. Suppression of cell motility and metastasis by transfection with human motility-related protein (MRP-1/CD9) DNA. The Journal of Experimental Medicine. 1993 May 1,;177(5):1231-7.

62. D'Angelo R, Aresta S, Blangy A, Del Maestro L, Louvard D, Arpin M. Interaction of Ezrin with the Novel Guanine Nucleotide Exchange Factor PLEKHG6 Promotes RhoG-dependent Apical Cytoskeleton Rearrangements in Epithelial Cells. Molecular Biology of the Cell. 2007 Dec 1,;18(12):4780-93.

63. Wald FA, Oriolo AS, Mashukova A, Fregien NL, Langshaw AH, Salas PJI. Atypical protein kinase C (iota) activates ezrin in the apical domain of intestinal epithelial cells. Journal of Cell Science. 2008 Mar 1,;121(5):644-54.

64. Ortega FJ, Pueyo N, Moreno-Navarrete JM, Sabater M, Rodriguez-Hermosa JI, Ricart W, et al. The lung innate immune gene surfactant protein-D is expressed in adipose tissue and linked to obesity status. International journal of obesity (2005). 2013 Dec;37(12):1532-8.

65. Alvarez-Curto E, Inoue A, Jenkins L, Raihan SZ, Prihandoko R, Tobin AB, et al. Targeted Elimination of G Proteins and Arrestins Defines Their Specific Contributions to Both Intensity and Duration of G Protein-coupled Receptor Signaling. The Journal of biological chemistry. 2016 Dec 30,;291(53):27147-59.

66. Tan N, Shaw NS, Vinckenbosch N, Liu P, Yasmin R, Desvergne B, et al. Selective Cooperation between Fatty Acid Binding Proteins and Peroxisome Proliferator-Activated Receptors in Regulating Transcription. Molecular and Cellular Biology. 2002 Sep 1,;22(17):6318.

67. Bzowska A, Luić M, Schröder W, Shugar D, Saenger W, Koellner G. Calf spleen purine nucleoside phosphorylase: purification, sequence and crystal structure of its complex with an N(7)-acycloguanosine inhibitor. FEBS Letters. 1995;367(3):214-8.

68. S E Ealick, S A Rule, D C Carter, T J Greenhough, Y S Babu, W J Cook, et al. Three-dimensional structure of human erythrocytic purine nucleoside phosphorylase at 3.2 A resolution. Journal of Biological Chemistry. 1990 Jan 25,;265(3):1812.

69. Willem-Jan Pannekoek, Jantine J.G. van Dijk, On Ying A. Chan, Stephan Huveneers, Jelena R. Linnemann, Emma Spanjaard, et al. Epac1 and PDZ-GEF cooperate in Rap1 mediated endothelial junction control. Cellular signalling. 2011(23):2056-64.

70. Quilliam LA, Lambert QT, Mickelson-Young LA, Westwick JK, Sparks AB, Kay BK, et al. Isolation of a NCK-associated kinase, PRK2, an SH3-binding protein and potential effector of Rho protein signaling. Journal of Biological Chemistry. 1996 Nov 1,;271(46):28772-6.

71. delCardayré SB, Ribó M, Yokel EM, Quirk DJ, Rutter WJ, Raines RT. Engineering ribonuclease A: production, purification and characterization of wild-type enzyme and mutants at Gln11. Protein engineering. 1995 Mar;8(3):261.

72. Johnson RJ, McCoy JG, Bingman CA, Phillips GN, Raines RT. Inhibition of Human Pancreatic Ribonuclease by the Human Ribonuclease Inhibitor Protein. Journal of Molecular Biology. 2007;368(2):434-49.

73. Tsang WY, Spektor A, Luciano DJ, Indjeian VB, Chen Z, Salisbury JL, et al. CP110 Cooperates with Two Calcium-binding Proteins to Regulate Cytokinesis and Genome Stability. Molecular Biology of the Cell. 2006 Aug;17(8):3423-34.

74. Tanji M, Horwitz R, Rosenfeld G, Waymire JC. Activation of Protein Kinase C by Purified Bovine Brain 14‐3‐3: Comparison with Tyrosine Hydroxylase Activation. Journal of Neurochemistry. 1994 Nov;63(5):1908-16.

75. Wang X, Grammatikakis N, Siganou A, Calderwood SK. Regulation of Molecular Chaperone Gene Transcription Involves the Serine Phosphorylation, 14-3-3ɛ Binding, and Cytoplasmic Sequestration of Heat Shock Factor 1. Molecular and Cellular Biology. 2003 Sep 1,;23(17):6013-26.

76. Béraud-Dufour S, Gautier R, Albiges-Rizo C, Chardin P, Faurobert E. Krit 1 interactions with microtubules and membranes are regulated by Rap1 and integrin cytoplasmic domain associated protein-1. FEBS Journal. 2007 Nov;274(21):5518-32.

77. Davis TL, Walker JR, Campagna-Slater V, Finerty PJ, Paramanathan R, Bernstein G, et al. Structural and Biochemical Characterization of the Human Cyclophilin Family of Peptidyl-Prolyl Isomerases. PLoS biology. 2010 Jul 27,;8(7):e1000439.

78. Ridley AJ, Paterson HF, Johnston CL, Diekmann D, Hall A. The small GTP-binding protein rac regulates growth factor-induced membrane ruffling. Cell. 1992 August 7,;70(3):401-10.

79. Meera Soundararajan, Francis S. Willard, Adam J. Kimple, Andrew P. Turnbull, Linda J. Ball, Guillaume A. Schoch, et al. Structural Diversity in the RGS Domain and Its Interaction with Heterotrimeric G Protein α-Subunits. Proceedings of the National Academy of Sciences of the United States of America. 2008 Apr 29,;105(17):6457-62.

80. Pierre-Olivier Angrand, Inmaculada Segura, Pamela VÃ¶lkel, Sonja Ghidelli, Rebecca Terry, Miro Brajenovic, et al. Transgenic Mouse Proteomics Identifies New 14-3-3-associated Proteins Involved in Cytoskeletal Rearrangements and Cell Signaling. Molecular & Cellular Proteomics. 2006 Dec 1,;5(12):2211-27.

81. Maruyama T, Nara K, Yoshikawa H, Suzuki N. Txk, a member of the non‐receptor tyrosine kinase of the Tec family, forms a complex with poly(ADP‐ribose) polymerase 1 and elongation factor 1α and regulates interferon‐γ gene transcription in Th1 cells. Clinical & Experimental Immunology. 2007 Jan;147(1):164-75.

82. Liu Y, Ross JF, Bodine PVN, Billiard J. Homodimerization of Ror2 Tyrosine Kinase Receptor Induces 14-3-3β Phosphorylation and Promotes Osteoblast Differentiation and Bone Formation. Molecular Endocrinology. 2007 Dec;21(12):3050-61.

83. Xu Y, Lu SZ, Zhao Y, Lu H, Jin Y, Dai M, et al. 14-3-3γ binds to MDMX that is phosphorylated by UV-activated Chk1, resulting in p53 activation. The EMBO Journal. 2006 Mar 22,;25(6):1207-18.

84. Saori Sato, Naoya Fujita, Takashi Tsuruo. Regulation of Kinase Activity of 3-Phosphoinositide-dependent Protein Kinase-1 by Binding to 14-3-3. Journal of Biological Chemistry. 2002 Oct 18,;277(42):39360-7.

85. Wijkstrom-Frei C, El-Chemaly S, Ali-Rachedi R, Gerson C, Cobas MA, Forteza R, et al. Lactoperoxidase and Human Airway Host Defense. American Journal of Respiratory Cell and Molecular Biology. 2003 Aug 1,;29(2):206-12.

86. Marieke Emonts, Fred C. G. J. Sweep, Nicolai Grebenchtchikov, Anneke Geurts-Moespot, Marlies Knaup, Anne Laure Chanson, et al. Association between High Levels of Blood Macrophage Migration Inhibitory Factor, Inappropriate Adrenal Response, and Early Death in Patients with Severe Sepsis. Clinical Infectious Diseases. 2007 May 15,;44(10):1321-8.

87. Maria Giulia Battelli, E. Lorenzoni, F. Stirpe. Milk Xanthine Oxidase Type D (Dehydrogenase) and Type 0 (Oxidase). Biochemical Journal. 1973;131.

88. Konno H, Minamiya Y, Saito H, Imai K, Kawaharada Y, Motoyama S, et al. Acquired xanthine dehydrogenase expression shortens survival in patients with resected adenocarcinoma of lung. Tumor Biol. 2012 Oct;33(5):1727-32.

89. Sidney W. Whiteheart, Irene C. Griff, Michael Brunner, Douglas O. Clary, Thomas Mayer, Susan A. Buhrow, et al. SNAP family of NSF attachment proteins includes a brain-specific isoform . Nature. 1993 March;362.

90. Andreeva AV, Kutuzov MA, Vaiskunaite R, Profirovic J, Meigs TE, Predescu S, et al. Gα12 Interaction with αSNAP Induces VE-cadherin Localization at Endothelial Junctions and Regulates Barrier Function. Journal of Biological Chemistry. 2005 Aug 26,;280(34):30376-83.

91. Kang TH, Park JH, Yang A, Park HJ, Lee SE, Kim YS, et al. Annexin A5 as an immune checkpoint inhibitor and tumor-homing molecule for cancer treatment. Nature communications. 2020 Feb 28,;11(1):1137.

92. Ooi CE, Dell'Angelica EC, Bonifacino JS. ADP-Ribosylation factor 1 (ARF1) regulates recruitment of the AP-3 adaptor complex to membranes. The Journal of cell biology. 1998 Jul 27,;142(2):391-402.

93. Sazanov LA, Peak-Chew SY, Fearnley IM, Walker JE. Resolution of the Membrane Domain of Bovine Complex I into Subcomplexes:  Implications for the Structural Organization of the Enzyme. Biochemistry. 2000 Jun 20,;39(24):7229-35.

94. James Murray, Bing Zhang, Steven W. Taylor, Devin Oglesbee, Eoin Fahy, Michael F. Marusich, et al. The Subunit Composition of the Human NADH Dehydrogenase Obtained by Rapid One-step Immunopurification. Journal of Biological Chemistry. 2003 Apr 18,;278(16):13619-22.

95. A. Martinez-Martinez, E. Munoz-Delgado, F.J. Campoy, C. Flores-Flores, J.N. Rodriguez-López, C. Fini, et al. The ecto-5P-nucleotidase subunits in dimers are not linked by disul¢de bridges but by non-covalent bonds. . 2000.

96. Arun Pal, Fedor Severin, Barbara Lommer, Anna Shevchenko, Marino Zerial. Huntingtin-HAP40 Complex Is a Novel Rab5 Effector That Regulates Early Endosome Motility and Is Up-Regulated in Huntington's Disease. The Journal of Cell Biology. 2006 February 13,;172(4):605-18.

97. Simon Hoffenberg, X. Liu, Lydia Nikolova, Hassan S. Hall, Wenping Dai, Robert E. Baughn, et al. A Novel Membrane-anchored Rab5 Interacting Protein Required for Homotypic Endosome Fusion. Journal of Biological Chemistry. 2000 Aug 11,;275(32):24661-9.

98. Xu D, Li Y, Wu L, Li Y, Zhao D, Yu J, et al. Rab18 promotes lipid droplet (LD) growth by tethering the ER to LDs through SNARE and NRZ interactions. The Journal of cell biology. 2018 Mar 5,;217(3):975-95.

99. Cunningham ML, Waldo GL, Hollinger S, Hepler JR, Harden TK. Protein Kinase C Phosphorylates RGS2 and Modulates Its Capacity for Negative Regulation of Gα11Signaling. Journal of Biological Chemistry. 2001 Feb 23,;276(8):5438-44.

100. Nigg EA, Silljé HH, Hanisch A. Timely anaphase onset requires a novel spindle and kinetochore complex comprising Ska1 and Ska2. The EMBO Journal. 2006 Nov 29,;25(23):5504-15.

101. Benny Björkblom, Artur Padzik, Hasan Mohammad, Nina Westerlund, Emilia Komulainen, Patrik Hollos, et al. c-Jun N-Terminal Kinase Phosphorylation of MARCKSL1 Determines Actin Stability and Migration in Neurons and in Cancer Cells. Molecular and Cellular Biology. 2012 Sep 1,;32(17):3513-26.

102. Yang W, Dicker DT, Chen J, El-Deiry WS. CARPs enhance p53 turnover by degrading 14-3-3σ and stabilizing MDM2. Cell Cycle. 2008 Mar 1,;7(5):670-82.

103. Kelly EE, Giordano F, Horgan CP, Jollivet F, Raposo G, McCaffrey MW. Rab30 is required for the morphological integrity of the Golgi apparatus. Biology of the Cell. 2012 Feb;104(2):84-101.

104. Armacki M, Joodi G, Nimmagadda SC, de Kimpe L, Pusapati GV, Vandoninck S, et al. A novel splice variant of calcium and integrin-binding protein 1 mediates protein kinase D2-stimulated tumour growth by regulating angiogenesis. Oncogene. 2014;33(9):1167-80.

105. Sellier C, Campanari M, Julie Corbier C, Gaucherot A, Kolb‐Cheynel I, Oulad‐Abdelghani M, et al. Loss of C9ORF72 impairs autophagy and synergizes with polyQ Ataxin‐2 to induce motor neuron dysfunction and cell death. The EMBO Journal. 2016 Jun 15,;35(12):1276-97.

106. Liu S, Leppla SH. Retroviral Insertional Mutagenesis Identifies a Small Protein Required for Synthesis of Diphthamide, the Target of Bacterial ADP-Ribosylating Toxins. Molecular Cell. 2003;12(3):603-13.

107. Kamijo K, Ohara N, Abe M, Uchimura T, Hosoya H, Lee J, et al. Dissecting the Role of Rho-mediated Signaling in Contractile Ring Formation. Molecular Biology of the Cell. 2006 Jan 1,;17(1):43-55.

108. Yin BWT, Kiyamova R, Chua R, Caballero OL, Gout I, Gryshkova V, et al. Monoclonal antibody MX35 detects the membrane transporter NaPi2b (SLC34A2) in human carcinomas. Cancer immunity. 2008 Feb 6,;8:3.

109. Kohama K, Okagaki T, Hayakawa K, Lin Y, Ishikawa R, Shimmen T, et al. A novel regulatory effect of myosin light chain kinase from smooth muscle on the ATP-dependent interaction between actin and myosin. Biochemical and Biophysical Research Communications. 1992 May 15,;184(3):1204-11.

110. Zhou X, Liu Y, You J, Zhang H, Zhang X, Ye L. Myosin light-chain kinase contributes to the proliferation and migration of breast cancer cells through cross-talk with activated ERK1/2. Cancer Letters. 2008;270(2):312-27.

111. Xiaolin Tian, Ramon U. Jin, Andrew J. Bredemeyer, Edward J. Oates, Katarzyna M. Błażewska, Charles E. McKenna, et al. RAB26 and RAB3D Are Direct Transcriptional Targets of MIST1 That Regulate Exocrine Granule Maturation. Molecular and Cellular Biology. 2010 Mar 1,;30(5):1269-84.

112. Huang L, Wong TY, Lin RC, Furthmayr H. Replacement of threonine 558, a critical site of phosphorylation of moesin in vivo, with aspartate activates F-actin binding of moesin. Regulation by conformational change. The Journal of biological chemistry. 1999 Apr 30,;274(18):12803.

113. Fu W, Cheng Y, Zhang Y, Mo X, Li T, Liu Y, et al. The Secreted Form of Transmembrane Protein 98 Promotes the Differentiation of T Helper 1 Cells. Journal of Interferon & Cytokine Research. 2015 Sep 1,;35(9):72-733.

114. Overmeyer JH, Wilson AL, Erdman RA, Maltese WA. The Putative “Switch 2” Domain of the Ras-related GTPase, Rab1B, Plays an Essential Role in the Interaction with Rab Escort Protein. Molecular biology of the cell. 1998 Jan;9(1):223-35.

115. Virginie F. Rhein, Joe Carroll, Shujing Ding, Ian M. Fearnley, John E. Walker. NDUFAF7 Methylates Arginine 85 in the NDUFS2 Subunit of Human Complex I*. 2013;288.

116. Gupta R, Somyajit K, Narita T, Maskey E, Stanlie A, Kremer M, et al. DNA Repair Network Analysis Reveals Shieldin as a Key Regulator of NHEJ and PARP Inhibitor Sensitivity. Cell. 2018 May 3,;173(4):972,988.e23.

117. Amir Porat, Yuval Sagiv, Zvulun Elazar. A 56-kDa Selenium-binding Protein Participates in Intra-Golgi Protein Transport. Journal of Biological Chemistry. 2000 May 12,;275(19):14457-65.

118. Pol A, Renkema GH, Tangerman A, Winkel EG, Engelke UF, Brouwer, Arjan P. M. de, et al. Mutations in SELENBP1 , encoding a novel human methanethiol oxidase, cause extraoral halitosis. Nature Genetics. 2018 -01;50(1):120-9.

119. Fabian Oceguera-Yanez, Kazuhiro Kimura, Shingo Yasuda, Chiharu Higashida, Toshio Kitamura, Yasushi Hiraoka, et al. Ect2 and MgcRacGAP Regulate the Activation and Function of Cdc42 in Mitosis. The Journal of Cell Biology. 2005 Jan 17,;168(2):221-32.

120. Katarzyna Modzelewska, Laura P. Newman, Radhika Desai, Patricia J. Keely. Ack1 Mediates Cdc42-dependent Cell Migration and Signaling to p130Cas. Journal of Biological Chemistry. 2006 Dec 8,;281(49):37527-35.

121. Bertram K, Agafonov DE, Liu W, Dybkov O, Will CL, Hartmuth K, et al. Cryo-EM structure of a human spliceosome activated for step 2 of splicing. Nature. 2017 Feb 16,;542(7641):318-23.

122. Yan ZJ, Delannoy M, Ling C, Daee D, Osman F, Muniandy PA, et al. A Histone-Fold Complex and FANCM Form a Conserved DNA-Remodeling Complex to Maintain Genome Stability. . 2010.

123. Singh TR, Saro D, Ali AM, Zheng X, Du C, Killen MW, et al. MHF1-MHF2, a Histone-Fold-Containing Protein Complex, Participates in the Fanconi Anemia Pathway via FANCM. Molecular Cell. 2010 Mar 26,;37(6):879-86.

124. Alpsoy A, Dykhuizen EC. Glioma tumor suppressor candidate region gene 1 (GLTSCR1) and its paralog GLTSCR1-like form SWI/SNF chromatin remodeling subcomplexes. The Journal of biological chemistry. 2018 Mar 16,;293(11):3892-903.

125. Wang W, Xue Y, Zhou S, Kuo A, Cairns BR, Crabtree GR. Diversity and specialization of mammalian SWI/SNF complexes. Genes & development. 1996 Sep 1,;10(17):2117-30.

126. Carlton JG, Bujny MV, Peter BJ, Oorschot VMJ, Rutherford A, Arkell RS, et al. Sorting nexin-2 is associated with tubular elements of the early endosome, but is not essential for retromer-mediated endosome-to-TGN transport. Journal of Cell Science. 2005 Oct 1,;118(19):4527-39.

127. Errabolu R, Sanders MA, Salisbury JL. Cloning of a cDNA encoding human centrin, an EF-hand protein of centrosomes and mitotic spindle poles. Journal of Cell Science. 1994 Jan 1,;107(1):9.

128. Jansen LET, Black BE, Yates JR, Cleveland DW, Foltz DR, Bailey AO. The human CENP-A centromeric nucleosome-associated complex. Nature Cell Biology. 2006 May;8(5):458-69.

129. Ma X, Ding W, Wang J, Wu G, Zhang H, Yin J, et al. LOC66273 Isoform 2, a Novel Protein Highly Expressed in White Adipose Tissue, Induces Adipogenesis in 3T3-L1 Cells. The Journal of nutrition. 2012 Mar;142(3):448-55.

130. Ha S, Lee S, Chung M, Choi Y. Mouse ING1 Homologue, a Protein Interacting with A1, Enhances Cell Death and Is Inhibited by A1 in Mammary Epithelial Cells. Cancer Research. 2002 Mar 1,;62(5):1275.

131. Khvotchev MV, Ren M, Takamori S, Jahn R, Sudhof TC. Divergent Functions of Neuronal Rab11b in Ca2+-Regulated versus Constitutive Exocytosis. Journal of Neuroscience. 2003 Nov 19,;23(33):10531-9.

132. Bernasconi R, Pertel T, Luban J, Molinari M. A dual task for the Xbp1-responsive OS-9 variants in the mammalian endoplasmic reticulum: inhibiting secretion of misfolded protein conformers and enhancing their disposal. The Journal of biological chemistry. 2008 Jun 13,;283(24):16446-54.

133. Yan Wang, Xiao Fu, Stephanie Gaiser, Michael KÃ¶ttgen, Albrecht Kramer-Zucker, Gerd Walz, et al. OS-9 Regulates the Transit and Polyubiquitination of TRPV4 in the Endoplasmic Reticulum. Journal of Biological Chemistry. 2007 Dec 14,;282(50):36561-70.

134. Sung-Woo Kim, Masaaki Hayashi, Jeng-Fan Lo, Young Yang, Jin-San Yoo, Jiing-Dwan Lee. ADP-ribosylation Factor 4 Small GTPase Mediates Epidermal Growth Factor Receptor-dependent Phospholipase D2 Activation. Journal of Biological Chemistry. 2003 Jan 24,;278(4):2661-8.

135. Anita Murthy, Charo Gonzalez-Agosti, Etchell Cordero, Denise Pinney, Cecilia Candia, Frank Solomon, et al. NHE-RF, a Regulatory Cofactor for Na+-H+Exchange, Is a Common Interactor for Merlin and ERM (MERM) Proteins. Journal of Biological Chemistry. 1998 Jan 16,;273(3):1273-6.

136. Yun CHC, Oh S, Zizak M, Steplock D. CAMP-mediated inhibition of the epithelial brush border Na+/H+ exchanger, NHE3, requires an associated regulatory protein. Proceedings of the National Academy of Sciences of the United States of America. 1997 Apr 1,;94(7):3010.

137. Cao TT, Deacon HW, Reczek D, Bretscher A, von Zastrow M. A kinase-regulated PDZ-domain interaction controls endocytic sorting of the beta2-adrenergic receptor. Nature. 1999 September 16,;401(6750):286.

138. James A. McNew, Morten SÃ¸gaard, Nina M. Lampen, Sachiko Machida, R. Ruby Ye, Lynne Lacomis, et al. Ykt6p, a Prenylated SNARE Essential for Endoplasmic Reticulum-Golgi Transport. Journal of Biological Chemistry. 1997 Jul 11,;272(28):17776-83.

139. Tai G, Lu L, Wang TL, Tang BL, Goud B, Johannes L, et al. Participation of the Syntaxin 5/Ykt6/GS28/GS15 SNARE Complex in Transport from the Early/Recycling Endosome to the Trans-Golgi Network. Molecular Biology of the Cell. 2004 Sep 1,;15(9):4011-22.

140. Watson P, Townley AK, Koka P, Palmer KJ, Stephens DJ. Sec16 Defines Endoplasmic Reticulum Exit Sites and is Required for Secretory Cargo Export in Mammalian Cells. Traffic. 2006 Dec;7(12):1678-87.

141. Bucci C, Bruni CB, Alifano P, Roberti V, Cantalupo G. Rab-interacting lysosomal protein (RILP): the Rab7 effector required for transport to lysosomes. The EMBO Journal. 2001 Feb 15,;20(4):683-93.

142. Hiroaki Konishi, Kyoko Tashiro, Yasunobu Murata, Hiromi Nabeshi, Emiko Yamauchi, Hisaaki Taniguchi. CFBP Is a Novel Tyrosine-phosphorylated Protein That Might Function as a Regulator of CIN85/CD2AP. Journal of Biological Chemistry. 2006 Sep 29,;281(39):28919-31.

143. Iwashita S, Fujii M, Mukai H, Ono Y, Miyamoto M. Lbc proto-oncogene product binds to and could be negatively regulated by metastasis suppressor nm23-H2. Biochemical and Biophysical Research Communications. 2004 Aug 6,;320(4):1063-8.

144. Chua K, Reed R. Human step II splicing factor hSlu7 functions in restructuring the spliceosome between the catalytic steps of splicing. Genes & development. 1999 Apr 1,;13(7):841-50.

145. Harry Mellor, Peter Flynn, Catherine D. Nobes, Alan Hall, Peter J. Parker. PRK1 Is Targeted to Endosomes by the Small GTPase, RhoB. Journal of Biological Chemistry. 1998 Feb 27,;273(9):4811-4.

146. Srougi MC, Burridge K. The Nuclear Guanine Nucleotide Exchange Factors Ect2 and Net1 Regulate RhoB-Mediated Cell Death after DNA Damage. PloS one. 2011 Feb 23,;6(2):e17108.

147. Zimmerman B, Kelly B, McMillan BJ, Seegar TCM, Dror RO, Kruse AC, et al. Crystal Structure of a Full-Length Human Tetraspanin Reveals a Cholesterol-Binding Pocket. Cell. 2016 Nov 3,;167(4):1041,1051.e11.

148. Building of the tetraspanin web: distinct structural domains of CD81 function in different cellular compartments. Molecular and cellular biology. 2006 Feb 1,;26(4):1373-85.

149. van Zelm MC, Reisli I, van der Burg M, Castano D, van Noesel, Carel J. M, van Tol, Maarten J. D, et al. An Antibody-Deficiency Syndrome Due to Mutations in the CD19 Gene. The New England Journal of Medicine. 2006 May 4,;354(18):1901-12.

150. Willment JA, Gordon S, Brown GD. Characterization of the Human β-Glucan Receptor and Its Alternatively Spliced Isoforms. The Journal of biological chemistry. 2001 Nov 23,;276(47):43818-23.

151. Shinya Morimoto, Noriyuki Nishimura, Tomoya Terai, Shinji Manabe, Yasuyo Yamamoto, Wakako Shinahara, et al. Rab13 Mediates the Continuous Endocytic Recycling of Occludin to the Cell Surface. Journal of Biological Chemistry. 2005 Jan 21,;280(3):2220-8.

152. Kang SW, Chae HZ, Seo MS, Kim K, Baines IC, Rhee SG. Mammalian Peroxiredoxin Isoforms Can Reduce Hydrogen Peroxide Generated in Response to Growth Factors and Tumor Necrosis Factor-α. The Journal of biological chemistry. 1998 Mar 13,;273(11):6297-302.

153. Yan Y, Sabharwal P, Rao M, Sockanathan S. The Antioxidant Enzyme Prdx1 Controls Neuronal Differentiation by Thiol-Redox-Dependent Activation of GDE2. Cell. 2009 Sep 18,;138(6):1209-21.

154. Dai X, Kolic J, Marchi P, Sipione S, Macdonald PE. SUMOylation regulates Kv2.1 and modulates pancreatic beta-cell excitability. Journal of cell science. 2009 Mar 15,;122(Pt 6):775-9.

155. Yeoh S, Pope B, Mannherz HG, Weeds A. Determining the Differences in Actin Binding by Human ADF and Cofilin. Journal of Molecular Biology. 2002 Jan;315(4):911-25.

156. Nguyen XP, Nakamura T, Osuka S, Bayasula B, Nakanishi N, Kasahara Y, et al. Effect of the neuropeptide phoenixin and its receptor GPR173 during folliculogenesis. Reproduction (Cambridge, England). 2019 Jul;158(1):25-34.

157. Chiu Y, MacMillan JB, Chen ZJ. RNA Polymerase III Detects Cytosolic DNA and Induces Type I Interferons through the RIG-I Pathway. Cell. 2009;138(3):576-91.

158. Díaz E, Schimmöller F, Pfeffer SR. A novel Rab9 effector required for endosome-to-TGN transport. The Journal of cell biology. 1997 Jul 28,;138(2):283-90.

159. Timson DJ, Reece RJ. Identification and characterisation of human aldose 1-epimerase. FEBS Letters. 2003;543(1):21-4.

160. Astier A, Trescol-Biemont M, Azocar O, Lamouille B, Rabourdin-Combe C. Cutting Edge: CD46, a New Costimulatory Molecule for T Cells, That Induces p120CBL and LAT Phosphorylation. The Journal of Immunology. 2000 Jun 15,;164(12):6091-5.

161. Tanaka M, Shinohara A, Miwa K, Iwamatsu A, Hanayama R, Nagata S. Identification of a factor that links apoptotic cells to phagocytes. Nature. 2002 May 9,;417(6885):182-7.

162. Sugano G, Bernard-Pierrot I, Laé M, Battail C, Allory Y, Stransky N, et al. Milk fat globule-epidermal growth factor-factor VIII (MFGE8) lactadherin promotes bladder tumor development. Oncogene. 2011 Feb 10,;30(6):642-53.

163. Raymond A, Ensslin MA, Shur BD. SED1/MFG‐E8: A Bi‐Motif protein that orchestrates diverse cellular interactions. Journal of Cellular Biochemistry. 2009 Apr 15,;106(6):957-66.

164. Wright SD, Ramos RA, Tobias PS, Ulevitch RJ, Mathison JC. CD14, a receptor for complexes of lipopolysaccharide (LPS) and LPS binding protein. Science. 1990 Sep 21,;249(4975):1431-3.

165. Roepman R, Bernoud-Hubac N, Schick D, Maugeri A, Berger W, Ropers HH, et al. The retinitis pigmentosa GTPase regulator (RPGR) interacts with novel transport-like proteins in the outer segments of rod photoreceptors. Human Molecular Genetics. 2000;9(14):2095-105.

166. (Received for publication, May, 1991). Characterization and Molecular Cloning aofPutative Binding Protein for Heparin-binding Growth Factors*. ;266.

167. Frederik P. Lindberg, Hattie D. Gresham, Elsa Schwarz, Eric J. Brown. Molecular Cloning of Integrin-Associated Protein: An Immunoglobulin Family Member with Multiple Membrane-Spanning Domains Implicated in α vβ 3-Dependent Ligand Binding. The Journal of Cell Biology. 1993 Oct 1,;123(2):485-96.

168. Knaus UG, Heyworth PG, Evans T, Curnutte JT, Bokoch GM. Regulation of phagocyte oxygen radical production by the GTP-binding protein Rac 2. Science. 1991 Dec 6,;254(5037):1512-5.

169. Conte M, Franceschi C, Sandri M, Salvioli S. Perilipin 2 and Age-Related Metabolic Diseases: A New Perspective. Trends in Endocrinology & Metabolism. 2016;27(12):893-903.

170. Rønnov-Jessen L, Villadsen R, Edwards JC, Petersen OW. Differential Expression of a Chloride Intracellular Channel Gene, CLIC4, in Transforming Growth Factor-β1-Mediated Conversion of Fibroblasts to Myofibroblasts. The American Journal of Pathology. 2002 Aug 1,;161(2):471-80.

171. Sun L, Sun TT, Lavker RM. Identification of a cytosolic NADP+-dependent isocitrate dehydrogenase that is preferentially expressed in bovine corneal epithelium. A corneal epithelial crystallin. The Journal of biological chemistry. 1999 Jun 11,;274(24):17334-41.

172. Chirco R, Jung K, Kim HC, Fridman R, Liu X. Identification of CD63 as a tissue inhibitor of metalloproteinase-1 interacting cell surface protein. The EMBO Journal. 2006 Sep 6,;25(17):3934-42.

173. Doyle EL, Ridger V, Ferraro F, Turmaine M, Saftig P, Cutler DF. CD63 is an essential cofactor to leukocyte recruitment by endothelial P-selectin. Blood. 2011 Oct 13,;118(15):4265-73.

174. van Niel G, Charrin S, Simoes S, Romao M, Rochin L, Saftig P, et al. The Tetraspanin CD63 Regulates ESCRT-Independent and -Dependent Endosomal Sorting during Melanogenesis. Developmental Cell. 2011 Oct 18,;21(4):708-21.
